# The Lifetime Impacts of the New Deal's Youth Employment Program

**DOI:** 10.1093/qje/qjae016

**Published:** 2024-06-08

**Authors:** Anna Aizer, Nancy Early, Shari Eli, Guido Imbens, Keyoung Lee, Adriana Lleras-Muney, Alexander Strand

**Affiliations:** Brown University, United States; Social Security Administration, United States; University of Toronto, Canada; Stanford University, United States; Federal Reserve Bank of Philadelphia, United States; University of California, Los Angeles, United States; Social Security Administration, United States

## Abstract

We study the lifetime effects of the first and largest American youth employment and training program in the United States—the Civilian Conservation Corps (CCC), 1933–1942. We match newly digitized enrollee records to census, World War II enlistment, Social Security, and death records. We find that longer service in the CCC led to improvements in height, health status, longevity, geographic mobility, and lifetime earnings but did not improve short-term labor market outcomes, including employment and wages. We address potential selection into CCC duration using several approaches, most importantly two newly developed control-function approaches that leverage unbiased estimates of the short-term effects of a randomized controlled trial of Job Corps (the modern version of the CCC). Our findings suggest that short- and medium-term evaluations of employment programs underestimate effects because they fail to capture lifetime effects and often ignore or underestimate health and longevity benefits that increase in magnitude at later ages.

## I. Introduction

The Great Recession of 2008 and the pandemic-induced recession of 2020 have renewed interest in understanding the effects of government programs implemented or expanded during economic crises. The Great Depression (1929–1941) was the largest economic downturn in modern history. In response, the federal government enacted a series of wide-scale poverty alleviation programs—the New Deal—which constituted the first and largest fiscal expansion during peacetime. These efforts were meant to provide temporary relief to individuals most affected by the Great Depression and spur economic activity. Yet many of these programs (e.g. Social Security, farm subsidies) remain in place today. Economists have long debated the effectiveness of these policies in the short run and particularly whether they helped or hindered economic recovery (Romer 1992). Substantially less is known about what New Deal programs did for individual beneficiaries, particularly over their lifetimes.

We conduct the first lifetime evaluation of the largest federal youth employment program in U.S. history created to address high youth unemployment during the Great Depression: the Civilian Conservation Corps (CCC). During the Great Depression, youth unemployment rates were estimated to be greater than 25% and as high as 60% (Rawick 1957; Salmond 1967). The CCC employed poor men aged 17 to 25 in unskilled, manual labor. Under the army's supervision, CCC enrollees were sent to work in camps in rural areas where they were also fed, housed, and given access to medical care. In addition to work experience, the CCC provided academic and vocational courses, helped recipients find employment, and provided cash transfers to participants’ families. Between 1933 and 1942, the CCC trained 3 million enrollees, representing one-third of all men aged 17–24, across 2,600 camps. Several programs in existence today, such as Job Corps, Youth Conservation Corps, and JobsFirstNYC, are modeled after the CCC (Levine 2010).

We create a new individual-level data set of CCC participants and their lifetime outcomes. We digitize administrative records for roughly 25,000 men from the CCC programs in Colorado and New Mexico, covering the population served by the program in those states between 1938 and 1943. Our data include information on their demographic characteristics, compensation, enlistment duration, and reasons for leaving the program. We match these enrollee records to the complete-count 1940 U.S. census, World War II enlistment records, Social Security Administration records, and individual death records. These data allow us to investigate the effects of the CCC on two related but independent determinants of well-being: lifetime earnings and longevity. We also collect data on several other short- and long-run measures that likely influence lifetime income and longevity, such as education, health, and geographic mobility.

To estimate the causal effects of the CCC, we take multiple approaches that exploit variation in the service duration of participants, similar to a dose-response analysis. First, we investigate how training duration affects outcomes in an ordinary least squares (OLS) setting. We use multiple methods to assess the bias in OLS estimates. These include estimating the stability of estimates when we vary the controls included, as in Chan, Gentzkow, and Yu (2022), bounding the effects based on Oster (2017), and an instrumental-variable technique that exploits the government's decision to ramp down or close camps as an exogenous source of variation in training duration. Finally, we use and extend new econometric techniques for causal inference that combine observational data from the CCC with data from a randomized controlled trial (RCT) of the Job Corps (JC), a modern-era job training program that was modeled after the CCC.

Training duration in the CCC varied from a few days to more than two years, with the average enrollee participating for nine months. We find that those who trained longer were not necessarily from higher or lower socioeconomic status (SES) backgrounds. Moreover, many enrollees with both short and long durations ended their training for arbitrary reasons. Thus, the direction of any omitted variable bias in OLS estimates is unclear. Controlling for extensive individual- and camp-level covariates has little effect on our estimates, which remain stable regardless of the set of controls included; moreover, Oster (2017) bounds remain tight. Placebo tests, in which we estimate whether duration predicts predetermined characteristics of the enrollees, also support our conclusion that omitted variables are not driving the relationship between training duration and long-term outcomes. Our IV estimates are similar to the OLS estimates. Though not definitive because of the lack of precision in the second stage, we interpret this as corroborating evidence that our OLS estimates likely suffer little bias.

Finally, in the spirit of seminal work by Lalonde (1986), we use the experimental data from the JC RCT to shed light on the internal validity of our research, which uses observational data. Although the JC data pertain to youth training that took place in the 1990s, the program was modeled after the CCC and shares many features. Moreover, we demonstrate that both JC and CCC participants were relatively disadvantaged compared with their contemporaries along many of the same margins.

Using the JC RCT, we implement two control-function approaches to correct for bias under different assumptions about bias stability. In the first approach, we follow Athey, Chetty, and Imbens (2020) and assume that the short-run treatment effect is the same across the JC and CCC. Any difference is interpreted as bias in our observational CCC estimates, which we correct for when we generate our long-run lifetime estimates. In the second approach, we extend Athey, Chetty, and Imbens (2020) and develop a new, more realistic approach for this setting in which we let the treatment effects differ but assume that the selection bias is similar in the JC and CCC. Our OLS estimates change very little when we add control functions from either approach, consistent with little bias in our observational CCC estimates.

We find no short- or medium-run labor market benefits associated with job training in the CCC. With respect to lifetime outcomes, however, we find significant benefits associated with longer training. Those who spend one year in the CCC have 5.2% higher lifetime earnings, live about a year longer, claim benefits (disability or pensions) 0.4 years later in life, and have 10% lower rates of Social Security Disability Insurance (SSDI) claims. These gains are consistent with and likely mediated by the improved education and health of the participants (measured by height and weight in young adulthood) as well as their increased geographic mobility toward healthier and richer areas. Interestingly, the gains in longevity are only apparent after age 55.

Overall, our results are consistent with the hypothesis that the program provided important in-kind goods and services to disadvantaged populations in a time of need, improving their long-term health, productivity, and longevity. However, while these gains were substantial, we estimate that they may not have been large enough to fully compensate individuals for large losses associated with the Great Depression.

This article makes several contributions to the existing literature. We provide the first estimates of the lifetime effects of a New Deal program on individual beneficiaries. Fishback (2017) provides a comprehensive survey of the microeconomic research on the short-run effects of New Deal programs based on local area (not individual-level) analyses. He reports that New Deal programs increased internal migration, lowered crime, and reduced mortality in the short run, although they tended not to improve employment (see also Fishback, Haines, and Kantor 2007; Arthi 2018). Our short-term results are consistent with this evidence.

Other work has investigated effects on the long-term economic path of regions receiving programs such as the Tennessee Valley Authority (Kline and Moretti 2014). Two recent papers examine the relationship between living in an area that received more New Deal programming and long-term individual outcomes. Modrek et al. (2022) investigate the effects of growing up in districts with more New Deal emergency employment on the long-term outcomes of children from Wisconsin and find mixed evidence regarding their benefits. Jou and Morgan (2023) evaluate the effect of New Deal transfers to counties on the longevity of their inhabitants and find benefits, particularly for young men. Neither study investigates the direct effects of a given program on its participants.

We also contribute to the extensive empirical literature investigating the effects of youth training programs. Our first contribution to this literature is to provide a more comprehensive evaluation of training program impacts by examining non–labor market outcomes over a participant's lifetime. In particular, we investigate effects on longevity, an important determinant of well-being (Jones and Klenow 2016), and on migration, another important determinant of health and economic outcomes. Recent work, including reviews of the job-training literature (Barnow and Smith 2015; Crépon and van den Berg 2016), has emphasized that training programs can have substantial benefits on non–labor market outcomes such as education (Cohen and Piquero 2009; Berk et al. 2020), crime (Heller 2014; Modestino 2019), or mortality (Gelber, Isen, and Kessler 2016), at least in the short to medium run. To our knowledge, no previous studies of youth training programs have investigated lifetime impacts, and no paper has tracked longevity. Longevity gains substantially increase the benefits of the programs measured by the marginal value of public funds (MVPF).

Our second contribution to the literature on job training is to study lifetime labor market outcomes. Our lifetime measures do not reflect periodic market fluctuations and incorporate gains from experience. Stochastic variation in labor market conditions causes the estimated effects of the returns to human capital investments to fluctuate (Rosenzweig and Udry 2020). As a result, point-in-time measures of labor market outcomes typically studied in job-training evaluations can only provide a partial and possibly biased assessment of training-program effects. This is particularly true if recipients are entering the labor market during periods of weak labor demand. Moreover, if the benefits of training are similar to the benefits of education, their return may only be observed over time. Indeed Card, Kluve, and Weber (2018) find that the short-term effects of more than 200 job-training programs on labor market outcomes are modest, but the treatment effects do appear to increase over time.^1^Kluve et al. (2019) arrive at similar conclusions in a meta-analysis of 113 youth training programs around the world. However, most evaluations track participants for only a few years—the longest evaluation we are aware of only tracks participants for 20 years.^2^

Our results confirm that the effects of the program are much larger over the lifetime than in the short term. As a result, the MVPF for the CCC that includes lifetime outcomes far exceeds one that includes only short- and medium-term impacts. We attribute this partly to the effects of the program on non–labor market outcomes, including improved health and increased geographic mobility, which can generate long-term but not necessarily short-term effects. These different short- and long-term results are consistent with a growing body of evidence suggesting that the short- and long-run effects of programs targeting health and human capital are often very different, with some programs showing fade-out of initial gains and others showing that benefits increase over time (Almond, Currie, and Duque 2018).

Our final contribution is methodological. We demonstrate how to combine observational data on lifetime outcomes with RCTs to make progress on estimating unbiased causal long-term program effects under different assumptions by extending the Athey, Chetty, and Imbens (2020) control-function approach. These methods can be applied to other settings where long-run observational and short-run experimental data are available.

## II. Background: The CCC Program

### II.A. Program Overview

The CCC was created to provide “relief of unemployment through the performance of useful public work and for other purposes.” The prevailing view at the time was that “relief through work,” would be more beneficial to the unemployed than the receipt of cash transfers (“direct relief”). Moreover, work would reduce the probability that youth would commit crimes and cause social disturbances (Brock 2005).

The untapped work capacity of idle youth would be used to create national parks, preserve forests, irrigate land, and address the damage of the Dust Bowl. Most camps had 200 enrollees at a time and were located close to work sites. The CCC program had 80% popular support, and many communities welcomed the camps and the resources they brought (Parham 1981; Paige 1985). As the program evolved, it added education components in 1934, which became mandatory in 1937, and military training in 1941. The program ended in 1942 due to the onset of World War II (Online Appendix Figure A.I).

### II.B. Eligibility

The CCC program was only open to male citizens who were unmarried, unemployed, and primarily between the ages of 17 and 25. Preference was given to those in greater need, and CCC enrollees were often selected from families already enrolled in relief programs. Government reports at the time confirm that enrollees were also poorly educated, with little work experience, as well as undernourished (McEntee 1942).^3^ Good physical condition, confirmed with an examination at enlistment, and no prior criminal activity were required. Camp assignment was based only on the needs of the camp, not enrollee preference.

### II.C. Compensation and Program Cost

Enrollees were required to work 40 hours a week and were paid $30 a month, $25 of which was sent home to a designated family member. The government paid for transportation to and from the camp and provided housing, uniforms, food, dental and medical care, and workers’ compensation insurance, costing an additional $36 a month. The estimated total annual cost per enrollee was $1,004 (Bureau of Labor Statistics 1941), equivalent to $18,000 in 2022, which is roughly half of per enrollee spending in Job Corps, the main federal youth training program today.

### II.D. Duration of Enrollment

Individuals initially enrolled for a six-month term and were allowed to re-enroll for a maximum of two years (four terms). CCC contracts could be terminated unilaterally by the government, based on governmental needs, at any point. Individuals also deserted, resigned, or were expelled prior to completing their contract. Enrollees could leave early if they had secured employment, were enrolled in a formal schooling program, or for “urgent and proper call” reasons, for instance, the death of a parent or some other personal emergency. They could be expelled for misconduct. Efforts were made to reduce enrollee turnover.

### II.E. The CCC in Colorado (CO) and New Mexico (NM)

CO and NM were relatively poor states at this time. Per capita annual personal income was $571 in CO and $329 in NM, while the nationwide average was $618 (U.S. Department of Commerce 1999). Due to the large number of parks and forests in these states, as well as the severe impact of the Dust Bowl, CO and NM had disproportionate participation in the CCC program. Enrollees in these states were disproportionately Hispanic.^4^

## III. Estimation Strategy and Identification Issues

We estimate the effect of the program on lifetime outcomes by comparing outcomes for those who served longer and shorter periods in a dose-response framework.^5^ This strategy of limiting comparisons to only those individuals who enrolled and served generates less potential selection bias than one that compares enrollees to non-enrollees. This strategy is similar to Flores et al. (2012), who estimate the returns to the number of courses taken in JC and to Lechner, Miquel, and Wunsch (2011) who evaluate effects of short and long training programs in Germany. We estimate the following specification:


(1) \begin{eqnarray*}
{Y_{icj}} = \alpha + \beta \times \left( {\textit{duration}\,\,of\,\,CCC\,\,\textit{servic}{e_{icj}}} \right) + {X_{icj}}\gamma + {e_{icj}},
\end{eqnarray*}

where ${Y_{icj}}$ is an outcome, such as employment or log of age at death for individual *i* born in year *c* training in CCC camp *j*, and ${X_{icj}}$ includes individual-level and camp-level covariates. The independent variable of interest is $duration\,\,of\,\,CCC\,\,\textit{servic}{e_{icj}},\,\,$measured in years. We cluster the standard errors at the application county-by-enrollment year-quarter (CQE) level, though the results are not sensitive to this choice.

The coefficient $\beta $ identifies the causal effect of duration on a given outcome only if duration is uncorrelated with other determinants of the outcome, conditional on the observables. The main threat to identification is the potential selection into duration, either positive or negative. Individuals with greater abilities may have trained longer because they benefited more from the program, were less financially constrained, and were able to better adapt to military life in camps (positive selection). Alternatively, poorer individuals may have had stronger incentives to train longer in the CCC because they were more in need of the monthly payment and had fewer outside options for employment (negative selection). In addition, although people could not select their camps, unobserved camp characteristics could affect duration and outcomes. For example, individuals might have stayed longer in camps with good weather, and good weather could improve long-term health (positive selection). Alternatively, demand for work might have been greater in places hardest hit by the Dust Bowl, leading enrollees to stay longer in unhealthy locations (negative selection).

To address these concerns, we take several approaches. First, we investigate the determinants of duration to assess possible selection. This includes examining the reasons enrollees leave before the end of term. Next, for a subset of the data for which we have extensive pre-enrollment information, we conduct placebo tests to estimate whether duration predicts pre-CCC enrollment outcomes (education, labor market experience, height and weight).

In addition, we use three standard methods to assess the potential for omitted variable bias: (i) following Chan, Gentzkow, and Yu (2022), we estimate multiple OLS regression specifications, varying the subset of covariates for which we control to examine the sensitivity of our coefficient estimates to the choice and order of covariates included; (ii) we estimate Oster (2017) bounds; and (iii) we instrument for training duration using exogenous variation in the reason for dismissal, which we discuss further in Section VI.

Finally, and most importantly, we implement a control-function approach to address any potential remaining selection bias. To do so, we rely on and extend methods developed by Athey, Chetty, and Imbens (2020), and use control functions generated from short-run estimates from the experimental JC data to adjust the estimates of the long-run effect of the program generated from the observational CCC data. We describe these control-function methods in greater detail later because they are more novel.

## IV. Data and Descriptive Statistics

### IV.A. Data Collection

#### 1. Colorado Enrollees

We digitized the original applications of all 18,644 individuals who applied to the CCC in Colorado between 1937 and 1942.^6^ The applications contain name, address, date of birth, place of birth, height, weight, race, Social Security number (SSN), marital status, whether the father or mother is living, number of brothers, number of sisters, number of family members in the household, rural status, farm ownership, occupation of main wage earner in household, educational details, employment status and history, name of designated allottee, and whether the individual was rejected. Except for information on height, weight, and race, which were collected upon medical examination, the information was self-reported. We observe the discharge information detailing the company and camp the person attended, reason for dismissal, date of dismissal, and whether the dismissal was honorable.

#### 2. New Mexico Enrollees

New Mexico CCC records include information on 9,699 individuals, covering the population of individuals that trained in state from 1938 to 1942. For each person, the records contain name, date of birth, address, family information (head of family, address of family, and relationship to enrollee), allottee information, enrollment date, assigned camp, date and reason for dismissal, and whether the dismissal was honorable. NM records contain substantially less information on participants than CO records because only discharge records are available.

#### 3. Camp-Level Data

For each camp we collect historical weather patterns (temperature and precipitation), the (Euclidean) distance of the camp to the closest towns and to each enrollee's hometown, the agency (and thus the type of work) that created the camp, and the average characteristics of enrollees at a given point in time, as well as census county-level characteristics, such as unemployment rates.

#### 4. Death Records

We link the CCC data with the Social Security Death Master File (DMF) and state-level death records to identify the date of death and SSN of each enrollee. We find death dates for 82% of recipients (Table I), representing much higher match rates than typically found in the literature.^7^ This match was done manually by trained genealogists at the BYU Record Linking Lab who found CCC enrollees in the collection of records kept by Ancestry.com and FamilySearch.org. A summary of this process is available in Online Appendix A4. We also match the data using automated methods as a robustness check.

**Table I:** Summary Statistics of the Civilian Conservation Corps Participants

	Mortality sample			Social Security sample		
	*N*	Mean	Std. dev.	*N*	Mean	Std. dev.
Service characteristics						
Duration of service (years)	17,086	0.836	0.707	12,455	0.816	0.701
First allottee amount (dollars per month)	16,566	21.69	3.687	12,097	21.70	3.683
Characteristics in enrollment application						
Age at enrollment	16,908	18.73	2.163	12,330	18.74	2.242
Enrollment year	17,086	1939	1.883	12,455	1939	1.889
Hispanic (imputed using Hispanic index)	17,086	0.450	0.498	12,455	0.432	0.495
Additional information in Colorado records						
Highest grade completed	10,828	8.685	2.071	8,225	8.700	2.055
Household size excluding applicant	6,090	4.772	2.590	4,730	4.725	2.575
Live on farm	6,259	0.255	0.436	4,846	0.252	0.434
Height (inches)	6,272	67.90	3.080	4,860	67.92	3.053
Weight (100 pounds)	6,356	1.390	0.171	4,922	1.391	0.171
Underweight	6,258	0.0684	0.252	4,849	0.068	0.253
Father living	6,146	0.805	0.396	4,765	0.806	0.396
Mother living	6,196	0.857	0.350	4,808	0.855	0.352
Match rates^a^						
Matched to death certificate data	23,722	0.817	0.387	12,455	0.991	0.0923
Matched to 1940 census	23,722	0.449	0.497	12,455	0.487	0.500
Matched to 1940 census among serving before 1940	9,890	0.433	0.496	5,151	0.483	0.500
Matched to WWII records	23,722	0.306	0.461	12,455	0.347	0.476
Post-CCC outcomes						
Age at death	17,086	73.62	12.03	12,348	74.76	9.245
Wage, conditional on working from 1940 census^b^	2,326	404.1	339.6	1,764	410.8	360.7

*Notes*. Basic sample includes records with duration (beginning and end date of enrollment), camp ID, and enrollment county. The analytical sample for the mortality analysis only includes those not missing death age, death age more than 45, and not missing peer and camp characteristics. Social Security sample are those we can match to the Master Beneficiary Record. When multiple records were found for a single person, we use the information in the first enrollment record.

^a^Match rates are calculated over the full analytical sample without death age restriction.

^b^Sample is enrollees serving before 1940.

#### 5. Social Security Records

Using SSNs, we match our data to the Master Beneficiary Record (MBR), maintained by the SSA, which contains information on individual lifetime earnings, disability (SSDI) claiming, and age at benefit claiming (Online Appendix A5) for those who apply for benefits.^8^ We match 53% of our records to the MBR records. For those who survived to age 65, we match 80%, indicating a high match rate for the targeted population. For individuals retiring after 1978, we observe the Average Indexed Monthly Earnings (AIME), which is computed as the average earnings of the highest 35 years of earnings after adjusting for inflation.

#### 6. 1940 and World War II Records

We match the enrollees to the 1940 federal census and to World War II enlistment records using the Abramitzky, Mill, and Perez (2020) expectation maximization (EM) algorithm (see Online Appendix B for details). The 1940 census includes education, location, and labor market information (employment, occupation, and wages). We successfully match 45% of all individuals and 43% of those who serve before 1940 to the census, and 31% to enlistment records. This lower match rate to enlistment records is expected because not all enlisted or served in World War II, even when they were eligible, and not all enlistee records survived.

### IV.B. Sample Selection

For our analysis, we restrict attention to individuals for whom we can observe duration of training, camp, and the outcome of interest. This results in a sample of 23,722 men out of 26,290 (Online Appendix Table A.I).

Our data suffer from some attrition but is unusually complete for the key long-term outcomes we examine. For the mortality analysis, we include the 17,086 men (72% of the original sample) who have information on age of death, who died after age 45 (this restriction avoids World War II deaths but does not affect our results), and for whom we can calculate relevant camp and peer characteristics. This estimation sample is generally representative of the complete data (Online Appendix Table A.II). For the lifetime outcomes from the SSA, our sample includes 12,455 individuals, again representative of the full sample in most cases, except for the age at death, which by construction is higher in the SSA sample because only those who survive to at least age 62 are eligible to apply for pensions, unless claiming for disability (Online Appendix Table A.IIa).

We investigate the extent of sample selection in our data and the effects of missing data on our estimates, using imputations in alternative specifications to generate a range of estimates, all of which are relatively tight. This is consistent with modest bias from nonrandom attrition (see Online Appendix Tables A.IIa and A.IIb for a full set of summary statistics).

### IV.C. Summary Statistics: CCC Training and Lifetime Outcomes

#### 1. Pre-CCC Characteristics

On average, enrollees were 18.7 years old, had completed 8.7 years of schooling, and came from a household with five people (Table I). One in four came from a farm, 19% had a deceased father, and 14% had a deceased mother. Despite height and weight examinations to exclude the unhealthy, 7% were underweight. Imputing the ethnic origin of the participants, we estimate that about 45% were Hispanic (see Online Appendix A9).

CCC enrollees came from poorer counties than the average men of the same age in CO and NM in the 1930 and 1940 census, consistent with being recruited from relief rolls (Online Appendix Table A.III). CO and NM enrollees were also more disadvantaged than the average CCC enrollee in the nation—they are substantially younger, are shorter, weigh less, have fewer dependents, and more of them have fewer than four years of schooling (Online Appendix Figure A.III).^9^ These findings are consistent with the fact that CO and NM were very poor states at that time. Not surprisingly, when we compare the CO and NM CCC enrollees with similarly aged men in the 1930 and 1940 censuses, we find that the CCC enrollees were relatively disadvantaged in terms of education, employment, and household income (Table II). We return to this in our control-function estimates where we explore and compare the extent of relative disadvantage in the CCC and JC samples.

**Table II:** Comparison of Jobs Corps and Civilian Conservation Corps Samples to Contemporary Censuses

	JC		CCC		
Sample	1990 census	JC participants	1940 census, national	1940 census, NM + CO	CCC participants
Education					
Mean	11.97	10.16	9.84	9.89	8.60
Standard deviation	2.23	1.54	2.66	2.62	2.11
Nonwhite share					
Mean	0.30	0.75	0.12	0.20	0.52
Standard deviation	0.46	0.43	0.32	0.40	0.50
Unemployed					
Mean	0.13	0.79	0.17	0.19	0.91
Standard deviation	0.33	0.41	0.38	0.39	0.29
Farm					
Mean	0.01	0.03	0.27	0.30	0.25
Standard deviation	0.12	0.16	0.44	0.46	0.43
Household size					
Mean	3.34	4.47	4.80	4.72	4.74
Standard deviation	1.92	2.04	2.56	2.71	2.60
Personal income (conditional on working)^a^					
Mean	9,711.78	2,078.08	582.84	522.58	189.74
Standard deviation	9,074.64	2,469.42	499.99	482.96	220.66
Household income share below national 41st percentile^b^					
Mean	0.41	0.81	0.41	0.49	0.63
Standard deviation	0.49	0.39	0.49	0.50	0.48

*Notes*. We compare characteristics of JC and CCC participants to men aged 16–24 in their contemporary censuses (1990 for JC and 1940 for CCC). The normalized difference is defined as (mean characteristic of JC or CCC – mean of contemporary census) / std. dev. of census. ^a^For CCC, the personal income comes from enrollees who serve after 1940 matched to the 1940 census. ^_b_^Household income is calculated by summing the wage income of all household members. This is done to ensure consistency across 1990 and 1940 censuses. For CCC, this is calculated for enrollees who serve after 1940 matched to the 1940 census. We use the 41st percentile as a meaningful comparison cutoff point since, in the JC baseline survey, around 81% responded that their household income was less than $18,000, which is the 41st percentile of household income in 1990 calculated using our measure of household income from the census.

#### 2. Post-CCC Outcomes

Consistent with CCC enrollees being disadvantaged at entry, they also had worse long-term outcomes than the cohort average. In particular, they died at younger ages and earned less in adulthood. The average enrollee eventually lived to age 73.6, one year less than male cohorts born in 1920 (who survived to age 45), according to SSA cohort life tables. They also had worse short-term outcomes: enrollees who served before 1940 earned $404 in annual wages in 1940, compared with $593 for men aged 18–32 in the 1940 census.

## V. Determinants of Training Duration

Duration in months spikes around 6, 12, 18, and 24 months, corresponding to one, two, three, and four terms (Figure I, Panel A), with 62% of individuals dropping out in the middle of their assignment. Among those who left before completing their term, 21% deserted, 14.5% were dismissed “for the convenience of the government” (e.g., the camp closed), 12% left for a job, and another 12% left because of an “urgent and proper call,” such as family sickness or death (Figure II, Panel A). Average training duration was 9.8 months.

**Figure I:**
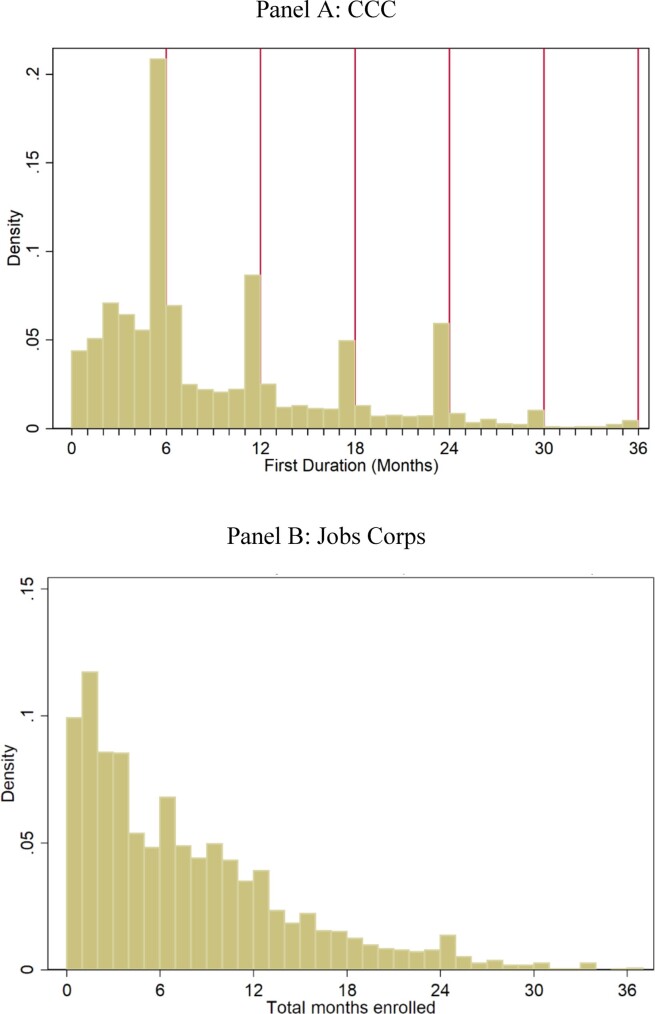
Distribution of Service Duration in the Civilian Conservation Corps Records and Jobs Corps We exclude durations greater than three years (less than 1% of the observations) in this figure. Mean duration in this sample is 9.44 months (std. dev. 7.47) for CCC and 5.8 months (std. dev. 6.6) for Jobs Corps. In Panel B, we exclude individuals who were not assigned to treatment and therefore have zero duration.

**Figure II:**
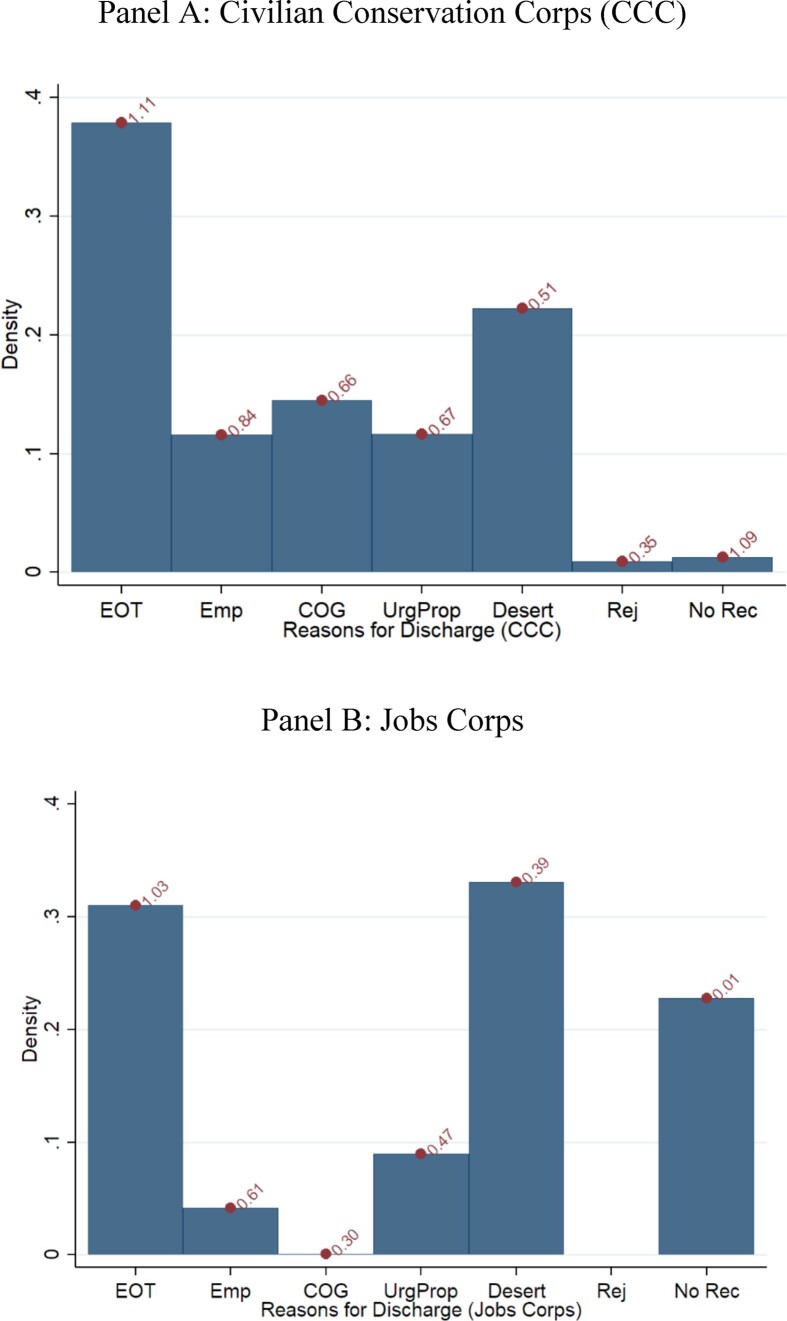
Distribution of Reason for Discharge Values on top of the bar graph are the mean duration (in years) for each category: EOT (end of term), Emp (employment outside the program), COG (convenience of the government), UrgProp (urgent and proper call), Desert, Rej (rejected), No Rec (no record). Reasons for Jobs Corps were harmonized to match with CCC's reasons for discharge.

To investigate the determinants of duration, we estimate OLS regressions of the duration of training as a function of individual, family, and camp characteristics. We include year-of-birth (YOB) fixed effects because different cohorts were eligible to train for different amounts of time (Online Appendix Figure A.IV). We also include application CQE fixed effects (e.g., one for those who enlisted in Denver, CO, in the first quarter for 1939). This addresses the fact that the number and types of camps that were opened varied over time and space, and the type of people who apply for training or other government benefits varies substantially with local economic conditions (Méndez and Sepúlveda 2012).

No clear relationship between personal characteristics and duration emerges in the data (Figure III and Online Appendix Table A.IV). There appear to be three groups of enrollees. One group served for longer because they were positively selected, such as those with more education or who were older. A second group serving longer are those coming from farms, larger households, or Hispanic households or who weighed less, indicating greater need and negative selection. A third group appears to have dropped out of the CCC for random reasons, cutting short their duration, such as unanticipated camp closures or emergencies at home.

**Figure III:**
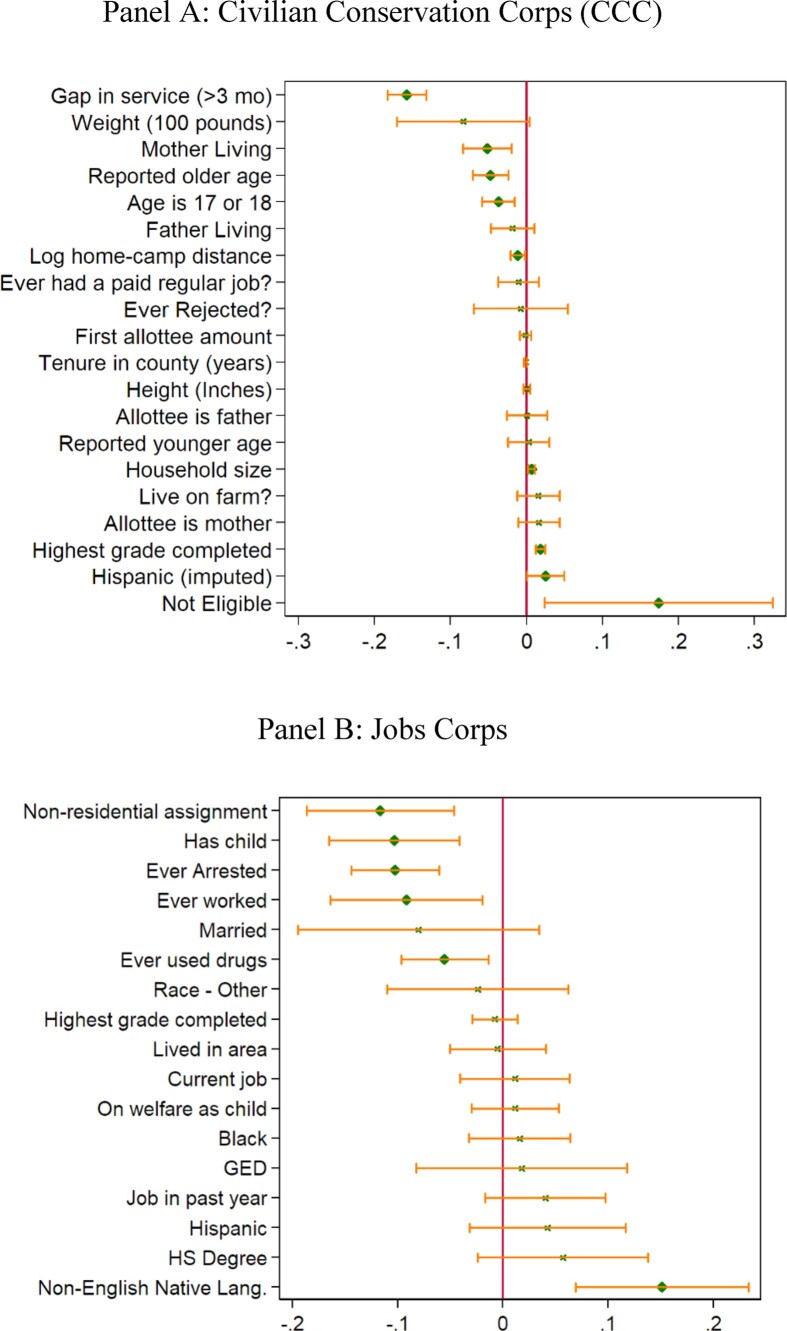
Determinants of Duration Estimates and 95% confidence intervals plotted for coefficient estimates of selected variables from regressing duration on various individual, camp, and peer characteristics. Coefficients in diamonds are statistically significant at the 95% level. Mean duration for the estimation sample is 0.84 years for CCC and 0.49 years for Jobs Corps. Full results of the regression estimates are shown in Online Appendix Table III.

The evidence also suggests that conditional on individual characteristics, place and time of enrollment, camp conditions were correlated with duration of training, but again, not in any consistent way (Online Appendix Table A.IV). Taken together, the primary evidence shows that desirable enrollee or camp traits did not necessarily lead to longer durations: there is no single narrative of selection.

## VI. The Long-Term Effect of CCC Training on Mortality, Lifetime Earnings, and Disability

We now investigate the effect of training duration on lifetime outcomes: mortality, earnings, disability claiming, and age at claiming disability or retirement.

### VI.A. Impact of CCC Duration on Longevity

Our graphical results indicate that the longer an enrollee trained, the longer he lived (Figure IV, Panel A). We present binscatter plots controlling for birth year using the methodology of Cattaneo et al. (2024). We fix the number of bins at 20 but allow polynomial approximation and smoothness to be chosen by the algorithm between 1 and 3.^10^ The results also indicate that we cannot reject a log-linear relationship between training duration and longevity.

**Figure IV:**
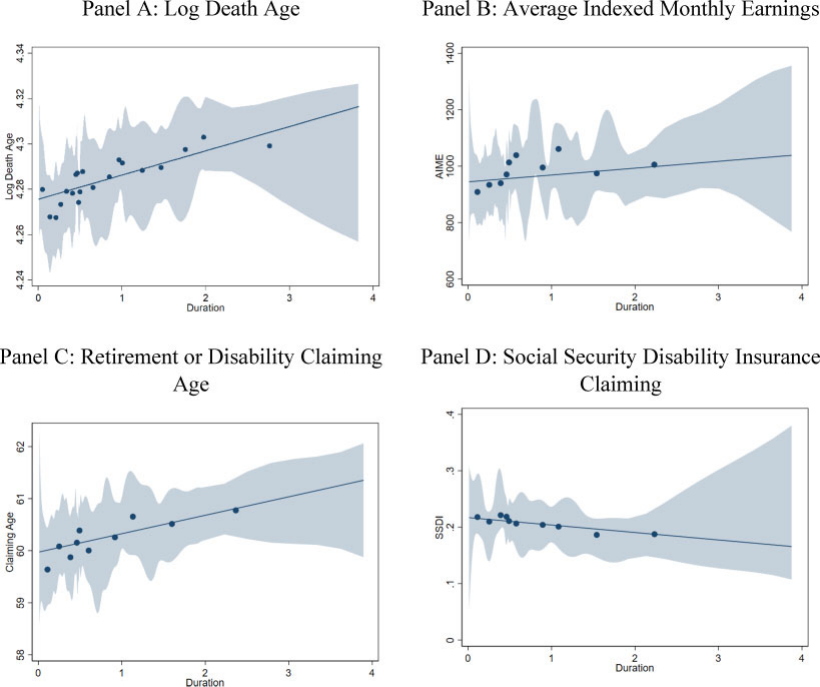
Binscatter Plots of Long-Term Outcomes Authors' computations based on death records (Panel A) and/or administrative program data matched to the Master Beneficiary Records (Panels B–D) and using the binscatter methodology of Cattaneo et al. (2024). It plots each variable controlling for birth year. We pick the number of bins (20 for Panel A, 10 for Panels B–D) and implement direct-plug-in data-driven choice of the optimal degree of polynomial and smoothness constraints for confidence band and bin means.

Given this relationship, we estimate an accelerated failure time model relating the log of the age at death on duration with added controls for the predetermined characteristics of the enrollees and the camps to examine whether and how our estimate of the effect of duration changes. Table III, Panel A, column (1) (with no controls) shows a very precise coefficient on duration of 0.013. Controlling for cohort and CQE fixed effects (column (2)) does not change the coefficient. Including family and individual characteristics lowers the coefficient from 0.013 to 0.011 (column (3)).^11^ The coefficient remains stable as we add more extensive controls. Adding camp characteristics in column (4) (mean precipitation in the camp, min and max temperature, type of camp, distance to closest city), peer characteristics in column (5) (average age, share Hispanic, average allottee amount, and gaps in service), or replacing camp characteristics by camp fixed effects in column (6) (or multiple other fixed effects as in Online Appendix Table A.VII) changes the coefficient very little. The magnitudes imply that one more year of training increased the age at death by one year (roughly 1.3% of 73.6 years of life). Given that the average duration was 9.85 months, the program increased age at death by 0.8 years for the average enrollee. The results are similar when we limit our sample to CO where the records contain more important baseline information for which we can control, such as education and height (column (7)).

**Table III:** Effect of CCC Service Duration on Longevity and Lifetime Labor Market Outcomes

Dependent variable	No controls	Add birth, county-qtr dummies	Add indiv. controls	Add camp chars.	Add peer chars.	Add camp FE	CO only
	(1)	(2)	(3)	(4)	(5)	(6)	(7)
Panel A: Longevity for the full sample (log death age)							
Duration of service (years)	0.013***	0.013***	0.011***	0.011***	0.013***	0.013***	0.013***
	(0.002)	(0.002)	(0.002)	(0.002)	(0.003)	(0.003)	(0.003)
Observations	17,086	17,086	17,086	17,086	17,086	17,086	10,944
* R*-squared	0.003	0.117	0.126	0.127	0.128	0.138	0.149
Mean death age	73.62	73.62	73.62	73.62	73.62	73.62	73.30
Oster bounds	[0.0127, 0.0136]						
Panel B: Average indexed monthly earnings (MBR sample claimed 1979 and later)							
Duration of service (years)	−0.083	67.048***	62.791***	62.450***	56.717***	50.134***	48.707***
	(10.181)	(12.186)	(12.501)	(12.616)	(13.723)	(14.690)	(17.236)
Observations	10,241	10,241	10,241	10,241	10,241	10,241	6,525
*R*-squared	0.000	0.188	0.204	0.205	0.206	0.222	0.236
Mean AIME	963.62	963.62	963.62	963.62	963.62	963.62	1,010.70
Oster bounds	[23.30, 143.34]						
Panel C: Retirement or Social Security Disability Insurance claiming age							
Duration of service (years)	0.506***	0.509***	0.452***	0.462***	0.427***	0.401***	0.554***
	(0.065)	(0.086)	(0.089)	(0.089)	(0.097)	(0.107)	(0.124)
Observations	11,712	11,712	11,712	11,712	11,712	11,712	7,768
*R*-squared	0.005	0.157	0.167	0.168	0.169	0.184	0.192
Mean claiming age	60.27	60.27	60.27	60.27	60.27	60.27	60.43
Oster bounds	[0.220, 0.462]						
Panel D: SSDI claiming (excluding unknowns)							
Duration of service (years)	−0.016**	−0.022***	−0.020**	−0.021**	−0.017*	−0.021**	−0.031**
	(0.006)	(0.008)	(0.009)	(0.009)	(0.009)	(0.010)	(0.012)
Observations	10,145	10,145	10,145	10,145	10,145	10,145	6,480
*R*-squared	0.001	0.154	0.161	0.163	0.164	0.181	0.205
Mean SSDI	0.21	0.21	0.21	0.21	0.21	0.21	0.20
Oster bounds	[−0.0304, −0.0181]						

*Notes*. Standard errors clustered at the level of county-by-year-quarter of enlistment are reported in parentheses. The sample is restricted only to those that died after age ≥ 45 and nonmissing peer and camp characteristics. Column (1) includes only duration of service as a regressor. Column (2) adds birth and county-year-quarter of enrollment fixed effects. Column (3) adds individual controls. Column (4) adds camp characteristics, such as distance from the nearest city and average temperature. Column (5) adds peer characteristics, where peers are defined as other enrollees serving in the same camp at the same time. Column (6) adds camp fixed effects and removes camp characteristics. Column (7) runs the regression specification in column (6) only for enrollees from our Colorado records. For a complete list of controls, refer to the text or Online Appendix Table V. MBR = Master Beneficiary Records. *** *p* < .01, ** *p* < .05, * *p* < .1.

#### 1. Assessing the Potential for Omitted Variable Bias

The coefficient stability across columns (1)–(7) suggests that selection bias may be small. Nevertheless, we conduct three additional exercises (described in Section III) to assess the potential for omitted variable bias.

#### i. Varying the set of controls

Following Chan, Gentzkow, and Yu (2022), we group the set of individual, family, and camp characteristics and the CQE FE into eight groups and estimate OLS regressions with all possible combinations of the covariate groups. We plot the minimum, average, and maximum coefficient estimates of training duration by the number of control groups included (Figure V). No minimum coefficient estimate includes zero, and the estimates appear relatively stable across the inclusion of additional control groups.

**Figure V:**
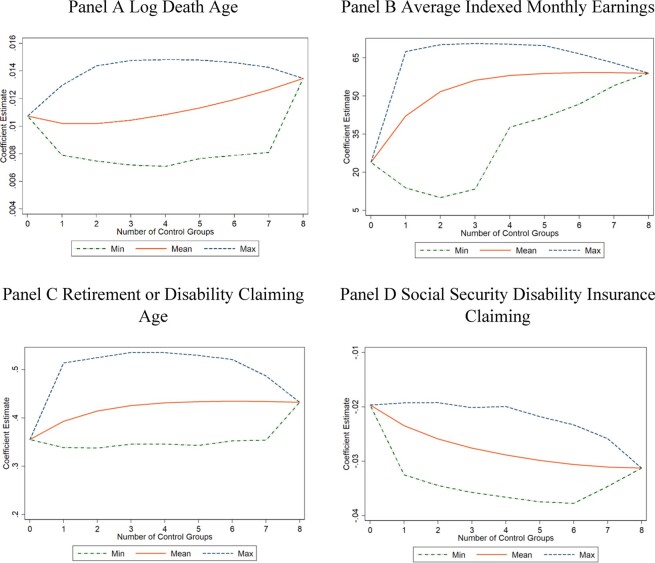
Visualizing Sensitivity to Controls We follow the methodology of Chan, Gentzkow, and Yu (2022) to visualize the sensitivity of our coefficient estimates on the choice and order of covariate inclusion. We split our covariates into eight groups and run the OLS regression using every combination of covariate groups, varying the number of groups included in total. We exclude cohort (birth year) fixed effects from the eight groups and include them in all specifications. The *x*-axis represents the number of covariate groups to be included, with 0 only including cohort fixed effects and 8 including all covariate groups. For example, for four control groups, the number of specifications would be 8 choose 4 = 70. The *y*-axis shows the average coefficient estimate (solid line) and minimum and maximum estimates (dotted/dashed lines).

#### ii. Calculating Oster bounds

We reestimate these coefficients under various assumptions about the unobservables following Oster (2017). If delta (the proportionality value) is assumed to be 1 (i.e., unobservables are as important as observables), then our coefficient would be 0.0136. Alternatively, if delta is assumed to be −1, we would estimate 0.0127 (Table III).^12^ Thus, one more year of training would increase the age at death by 0.96 to 1.02 years. Both exercises provide further evidence that our results are unlikely to be driven by omitted variables.

#### iii. IV estimates

For the IV approach, we take advantage of the fact that some people were dismissed “for the convenience of the government,” which typically occurred when a camp was winding down or closing upon project completion.^13^ We consider this timing to be exogenous in a camp. Since most enrollees who were discharged for the convenience of the government (COG) did so at the time they completed their term, we select those who also completed their terms and left voluntarily (the “end of term” or EOT group) as the appropriate comparison group. Our assumption is that had the COG group not been dismissed by the government, they would have either finished a longer enlistment term or reenlisted, making EOT enrollees a suitable counterfactual for COG enrollees. This is supported by COG and EOT enrollees having a similar distribution of training duration, with spikes at the completion of terms, but EOT enrollees serving more terms, as expected (Online Appendix Figure A.VI). We construct the instrument as an indicator equal to one if the enrollee was dismissed by COG and zero if EOT. Because the sample we use for this approach is smaller and the standard errors substantially larger, we view these results as complementary to the main analysis.

We present estimates from the first and second stages (along with the OLS counterparts for this smaller sample) based on the specification with peer characteristics in Table IV.^14^ The instrument is predictive of duration: being dismissed for the convenience of the government reduces average duration by 0.17 of a year or two months. The *F*-statistic is large, more than 40.^15^ The IV estimates for longevity show a second-stage estimate of 0.013, similar in magnitude to the OLS estimates for the IV sample (0.014), which is almost identical to our main 0.013 estimate in Table III. Although the IV estimates in this smaller sample are not significant at conventional levels, we cannot reject that they are the same as the OLS estimates.

**Table IV:** Instrumental-Variable Estimates of the Effect of CCC on Longevity and Lifetime Labor Market Outcomes

Outcome	Log age at death	Average indexed monthly earnings	Retirement or disability claiming age	Social Security Disability Insurance
IV	0.013	287.937*	1.189	0.018
	(0.038)	(169.308)	(1.574)	(0.114)
OLS	0.014***	42.574**	0.511***	−0.023*
	(0.004)	(20.402)	(0.143)	(0.013)
First stage	−0.165***	−0.167***	−0.162***	−0.170***
	(0.022)	(0.024)	(0.025)	(0.024)
*F*-statistic	55.11	47.42	42.08	48.67
Observations	9,049	5,529	6,169	5,474

*Notes*. Standard errors clustered at the level of county-by-year-quarter of enlistment are reported in parentheses. The sample is restricted only to those that died after age ≥ 45, have nonmissing peer and camp characteristics, and those who were dismissed after the end of term or for the convenience of the government. Our IV is whether the enrollee was dismissed for convenience of the government. We present the 2SLS instrumental-variable regression’s coefficient on duration, the OLS regression coefficient on duration, the first-stage coefficient on our instrument from regression of duration on the instrument, and the *F*-statistic on the instrument from the first stage. Controls include birth and county-year-quarter of enrollment fixed effects, individual controls, camp characteristics (such as distance from the nearest city and average temperature), and peer characteristics, where peers are defined as other enrollees serving in the same camp at the same time. We do not include camp fixed effects because they are highly predictive of camp closures—the results are similar with camp fixed effects but the standard errors are even larger. For a complete list of controls, refer to the text or Online Appendix Table V. *** *p* < .01, ** *p* < .05, * *p* < .1.

#### 2. Timing of Longevity Gains

To examine when over one's lifetime the longevity gains accrue, we present the results of the regression of probability of surviving to age *x* on duration, for every *x* between 45 and 90 (Figure VI). The coefficients are small and statistically insignificant at younger ages when survival is very high. They become positive and statistically significant starting at age 56, peak between ages 68 and 78, and decline thereafter. As a function of the baseline survival rate, which is declining throughout, the effects rise until age 67 and then decline. Thus, the benefits of the program in terms of mortality are not apparent until after individuals reach mature ages. This is consistent with the model of Lleras-Muney and Moreau (2022): health gains in childhood and young adulthood only manifest themselves in older ages when health starts to decline, and the least healthy individuals die.

**Figure VI:**
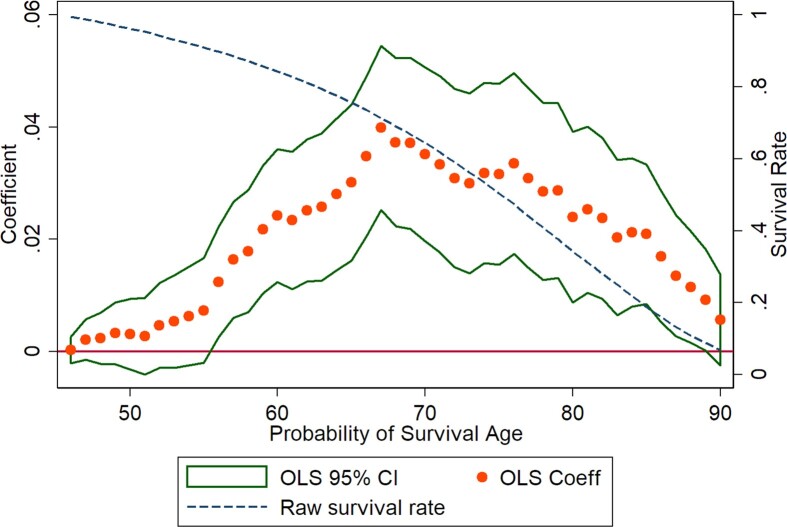
Effect of Service Duration on the Probability of Survival to Different Ages On the left *y*-axis, this figure reports the coefficients (and standard errors) from running linear regressions of the probability that the person survived to a given age on duration, where age ranges from age 45 to age 90. The regressions use the administrative data we collected and control for all observables at baseline (see Table III for details). On the right *y*-axis we plot the survival rate.

#### 3. Sample Attrition

About 18% of the original sample is missing information on age at death. We assess whether missing age at death is systematically related to training duration (with or without conditioning on covariates). Online Appendix Table A.IX, Panel A shows that without controls, the missing rates are not a function of training duration. But conditional on camp, family and individual characteristics, age at death is about 9% $\left( \frac{1.7}{18} \right)$ less likely to be missing for those who trained for an additional year. This suggests that differential attrition could bias our OLS estimates. To address this issue, we estimate survival models where we make different assumptions about the missing data. The effect of duration on longevity is consistently positive and statistically significant under various imputation approaches (Online Appendix Table A.X).

#### 4. Alternative Measures of Longevity

Our main results use the information found by trained genealogists from multiple sources to determine the age at death. However, we can replicate the results using death age retrieved from machine matching our records to the DMF. We continue to estimate a positive and statistically significant coefficient of duration on age at death that is similar in magnitude to our main estimates (Online Appendix Table A.XI).

### VI.B. Impact of CCC Duration on Lifetime Income

We estimate the impact of program duration on lifetime income as proxied by the AIME, which is the average of an individual's best 35 years of real earnings, used by the SSA to calculate pension amounts. The AIME is only available for those claiming after 1979, but a specification check suggests that the results would be similar if we were to extend to those claiming prior to 1979.^16^

We present graphical results using a binscatter plot in Figure IV (Panel B) and OLS regressions in Table III, Panel B. Both the binscatter plot and the OLS results show that, controlling for birth cohort, there is a significant relationship between training duration and lifetime earnings (column (2)). The estimates imply an increase of $67 additional monthly earnings for those who participated for one year, a 7% increase relative to average earnings.^17^ As we add more controls across the columns, the estimated coefficient declines to $50, or 5.2% of average monthly earnings (column (6)). This coefficient is less stable than the one for longevity. As a result, the Oster bounds are also wider (23–143) but still greater than zero. When we perform the previous sensitivity exercise, varying the number and types of controls (Figure V, Panel B), the estimates appear to reach stability if at least three of the eight sets of controls are included, after which the variance in estimates also declines. Again, the minimum values do not include zero for any number of control groups. The IV estimates (Table IV) suggest a positive impact that is greater than the OLS estimates and statistically significant at the 10% level. The IV estimates suggest increases of about $290 (a roughly 30% increase relative to the mean), although these estimates are not statistically different from OLS estimates.

These results do not appear to be driven by sample selection or attrition in the SSA data. Duration is uncorrelated with whether we match an enrollee to MBR. Nor does the effect of duration on longevity, an outcome available for enrollees not matched to MBR, change when we limit to the sample matched to the MBR (Online Appendix Table A.IX, Panels B and C).

We can compare our estimated returns from a year in the CCC to the returns from a year of schooling. OLS estimates of the returns to schooling during this period from other sources range from 5% (Goldin and Katz 2000) to 8% (Clay, Lingwall, and Stephens 2012). Thus, the returns to one year of CCC training (5.2%) are on the lower end of the returns to a year of schooling.

### VI.C. Impact of CCC Duration on Age at Benefit Claiming and SSDI Claiming Rate

We estimate the impact of CCC duration on the age at which individuals first claim benefits from the SSA (either disability or pensions), presenting the binscatter plots in Figure IV, Panel C and OLS results in Table III, Panel C. We find that one year of CCC enrollment increases the age at claiming benefits by almost half a year, with a mean age at claiming of 60 years, suggesting CCC men were in better health, retired later, and lived longer. Oster bounds are tight, ranging from 0.220 to 0.462, and the estimates are very stable regardless of control groups included (Figure V). This is consistent with existing work showing that early retirement is associated with worse health as proxied by death at younger ages (Waldron 2001; Fitzpatrick and Moore 2018). The IV estimates are positive, statistically insignificant, and larger than OLS estimates at 1.2 instead of 0.5 (Table IV). But again, they are not statistically significantly different from OLS estimates.

We can examine this health channel directly by looking at how duration affects SSDI claiming, another measure of health, in Figure IV, Panel D (binscatter), Table III (OLS), and Figure V (sensitivity analysis). When the full set of controls is included, we find that one year in the CCC reduces claiming by 2.1 percentage points, or 10% relative to the mean claiming rate in the sample of 21%. Oster bounds range from −0.03 to −0.018, and again the estimates are stable to the controls we include (Figure V). The IV estimates are positive, though again they are not statistically significant or different from OLS. The OLS estimate is comparable to the 20-year follow-up of the JC RCT that finds a 2.4 percentage point decrease in SSDI claiming for older participants (Schochet 2021).

Overall, we find that CCC participation improves income and health in the long run as measured by delayed benefit claiming, reduced SSDI claiming, and greater longevity. In Online Appendix Table A.XIII we explore heterogeneity. The only consistent finding along all long-term outcomes is that the young benefited more from the program, contrary to Card, Kluve, and Weber's (2018) finding that youths tend to display few gains.

## VII. Short-Term Outcomes: Evidence from the 1940 Census and WWII Records

We estimate the short-run effect of CCC enrollment to compare our estimates with existing work on the short-run impact of more recent job-training programs and to better understand the mechanisms behind the estimated long-run effects of the CCC. We first investigate the effects of CCC duration on employment and wages in 1940, the short-run outcomes usually assessed in evaluations of job-training programs. Then we investigate other mechanisms, such as formal education, health improvements, and geographic mobility, all of which have been associated with improved longevity and labor market outcomes in previous work.

### VII.A. Labor Market Outcomes: Evidence from the 1940 Census

For this analysis, we constrain our sample to the 9,518 men who participated in CCC before January 1, 1940, and have all relevant covariates, 43% of whom we find in the 1940 census. Duration is unrelated to whether we locate an enrollee's census record.^18^

CCC training duration has little effect on the short-run labor market outcomes of CCC participants (Table V and Online Appendix Table A.XIV). Most men (91%) are in the labor force, and longer CCC training had at best a very small effect on this outcome: a 2.0% increase relative to the mean. We observe no effect on employment (conditional on labor force participation) as reported in the 1940 census. There is a small, negative, and imprecise effect of duration on earnings in 1940. Our results suggest that the labor market effects of job training are much greater in the long run than in the short run, consistent with recent reviews.

**Table V:** Effect of CCC Service Duration on Labor Market Outcomes Observed in the 1940 Census and WWII Records

Data source	1940 census outcomes (individuals enrolling pre-1940)					WWII enlistment records			Combined
Outcome	Found in census records	In labor force	Weeks worked in 1939^a^	Total annual wage in 1939^a^	Ln total annual wage | working^a^	Found in WWII records: served	Height (inches)	Body Mass Index	Education (years)
	(1)	(2)	(3)	(4)	(5)	(6)	(7)	(8)	(9)
Duration of service (years)	0.012	0.018*	0.316	−14.977	−0.015	0.038***	1.143***	1.017***	0.169***
	(0.012)	(0.010)	(1.194)	(26.394)	(0.062)	(0.007)	(0.221)	(0.204)	(0.040)
Observations	9,518	4,052	2,361	2,149	1,750	22,964	5,770	5,287	9,586
Mean dependent variable	0.43	0.91	27.88	383.71	471.25	0.31	67.55	21.53	9.23

*Notes*. Standard errors clustered at the level of county-by-year-quarter of enlistment are reported in parentheses. This table only displays the specification in Table III, column (6) for different outcomes observed in the 1940 census. The sample includes enrollees who serve before 1940 and can be matched to a 1940 census record, and who have all relevant control variables across all specifications. For results on all specifications, refer to Online Appendix Tables XIV and XV. *** *p* < .01, ** *p* < .05, * *p* < .1. ^a^Sample are those whose first term in CCC is before 1939 and are not enrolled in 1939. Census asks labor force and work status on the week before the census enumeration, while wage information and weeks worked is asked for the year before the census 1939.

### VII.B. Health and Military Service: Evidence from WWII Enlistment Records

We estimate the short-run impact of the CCC on health measured by height and weight in war enlistment records. Unlike the 1940 census records, duration does predict their presence in the World War II records: an additional year of training leads to a robust and significant 3.8 percentage point increase in the probability we find the individual in the enlistment records, a 12% increase relative to the mean (Table V, column (6) and Online Appendix Table A.XV). This result is not surprising: the army organized and administered life in the camps, and CCC men who trained for a long time were well acquainted with military life. Two percent of men in our sample ended their CCC engagement to enlist in the military directly. Given that we have not found differential matching rates to census and SSA databases, we do not believe differential matching explains this result. Instead, we conclude that the program increased the likelihood of serving in the military. This could reflect greater familiarity with the military after serving in the CCC, or it could reflect the acquisition of additional noncognitive skills and better health that increased the likelihood of success in the military. Indeed, the final report of the CCC noted that “enrollees made splendid soldier material” (McEntee 1942).

We find that one more year of training translated into roughly one more inch of height among enlisted men. This effect is large by historical standards: for example, it took British men 100 years for their average height to increase by six inches (Fogel 1994). This result holds conditional on height at CCC enlistment, indicating additional growth after CCC enrollment rather than initial differences in height. There are multiple reasons we believe the program increased heights of CCC enrollees. First, national reports of the CCC program show that the average height gain in the CCC was half an inch (McEntee 1942). Second, these results are consistent with existing work showing that undernourished populations grow more slowly and achieve their final adult height at older ages (Steckel 1986). Indeed, estimates suggest that at the turn of the twentieth century men continued growing until age 26 (Sullivan 1971). Individuals in the CCC were poor, and they received food and medical care, including vaccinations, as part of their participation in the program, likely improving their nutritional status. Our estimates are likely larger than the CCC average gain in height because our population is more disadvantaged than the average CCC enrollee. Finally, we find that the gains mostly come from the youngest in our sample, as one would expect. For enrollees 20 years or older, the gains are about 0.822 inches and statistically insignificant compared to 1.143 inches for the whole sample (Online Appendix Table A.XV). Moreover, 9% of our sample of CCC enrollees were likely younger than they reported.

Consistent with this, we observe a 4.7% increase in BMI, a common indicator of short-term nutrition (Table V, column (8)). The final CCC report documents an average weight gain of enrollees during the program of 11 pounds, translating to an average 8% increase in BMI (McEntee 1942). Our results suggest that half of these gains persisted. These results showing improved health in the short run are consistent with our finding that CCC service lowered SSDI claiming and increased longevity in the long run. We conclude that the CCC improved overall health among participants in the short and the long run.

### VII.C. Effects on Education and Geographic Mobility

We also estimate the impact of CCC duration on formal years of schooling and geographic mobility, which are observed in both the census of 1940 and World War II enlistment records. For these outcomes, we combine information from the two sources to maximize sample size. We control for the time since discharge (or equivalently the year of observation) to account for the fact that the outcomes are not observed at the same time for all enrollees.

We estimate a positive and statistically significant effect of duration on years of schooling of about 0.17 years, controlling for education at baseline (Table V, column (9)).^19^ This represents one-tenth of the standard deviation in schooling in the WWII records and is larger than the effect of many education policies, such as child labor laws, during the early twentieth century (see Lleras-Muney 2002; Goldin and Katz 2008). This effect likely represents a combination of additional schooling completed as part of the CCC and schooling obtained after discharge from the CCC. CCC reports indicate that 8% of men obtained additional schooling during the program. Assuming 8% obtained one more year of school, this would result in a gain in years of schooling of 0.08. Given that 3.5% of enrollees in our data cited education as the reason for leaving the program, post-CCC education gains likely account for the rest.

Finally, we examine the relationship between duration and short- and long-term geographic mobility by comparing the county of individuals in their original CCC application with the county of residence indicated in the 1940 census records, the war records, and in the death certificates. Thirty-three percent of participants moved in the short term (by 1940 census or WWII enrollment), and one more year of training increased this probability of moving by 5.7 percentage points, or 17% relative to the mean (Table VI, column (2) and Online Appendix Table A.XVI for all specifications). This is substantial, particularly during a period characterized by historically low migration nationwide.^20^ Moreover, when CCC men moved, they moved to locations with higher paying weekly or annual wages (as of 1940) and lower mortality, measured by the median county level mortality from 1950 to 1968 (Table VI, columns (3) and (4)). Over the long run, however, most individuals moved and the effect of duration on mobility fades (Table VI, columns (5)–(7)).

**Table VI:** Effect of CCC Service Duration on Geographic Mobility over the Lifetime

	Short-term mobility (place in 1940 census or WWII enlistment differs from place of residence at enrollment in CCC)				Long-term mobility (place of death differs from place of enrollment in CCC)		
	Moved to a different state	Moved to a different county	New county has higher yearly wage than sending county	New county has above median mortality rate (1950–1968)	Died in a different state	Died in a different county	New county has above median mortality rate (1950–1968)
	(1)	(2)	(3)	(4)	(5)	(6)	(7)
Duration of service (years)	0.026***	0.057***	0.045**	−0.043*	−0.029**	0.003	0.006
	(0.007)	(0.011)	(0.020)	(0.023)	(0.015)	(0.012)	(0.015)
Observations	9,568	9,568	3,165	3,175	7,235	7,079	5,313
Mean dependent variable	0.09	0.33	0.65	0.38	0.5	0.79	0.25

*Notes*. Standard errors clustered at the level of county-by-year-quarter of enlistment are reported in parentheses. This table only displays the specification in Table III, column (6) for different outcomes observed in the 1940 census. The sample includes enrollees who served before 1940 and can be matched to a 1940 census record. For results on all specifications, refer to Online Appendix Table XVI. *** *p* < .01, ** *p* < .05, * *p* < .1.

In sum, in the short run, job training had no impact on labor market outcomes. It did improve health and increase schooling and geographic mobility. These short-run effects on health and human capital help explain the long-run/lifetime impacts on earnings, disability, age at retirement, and longevity. Overall, these findings are in line with the evidence from developing countries reported in Kluve et al.'s (2019) review: comprehensive programs like the CCC work better than programs that narrowly focus on a single skill, perhaps because they have benefits along many margins.

### VII.D. Falsification Tests

We conduct a final check of the validity of our OLS approach. For CO enrollees, we have baseline measures for several outcomes: height, weight, education, and prior labor market experience. In our main results we control for these, but we can also test if duration predicts these pre-intervention outcomes. The only pre-CCC outcome that duration predicts is education. Training duration does not predict height, weight, or labor market activity prior to enrollment (Online Appendix Table A.XVII).

These results suggest that by and large our approach produces unbiased estimates of the effects of the program, but some bias may remain. In the next section we estimate the long-run and lifetime effects of the CCC using a novel control-function approach that incorporates evidence from the JC RCT to address any remaining selection.

## VIII. Control-Function Approaches: Incorporating Evidence from the Job Corps RCT to Address Selection Bias in the CCC

To further corroborate our findings, we implement a recently developed approach to address selection that relies on a different set of assumptions and leverages information from an existing randomized experiment.

### VIII.A. Overview

In estimating the long-run and lifetime effects of the CCC, we build on the seminal work of Lalonde (1986), who used experimental evidence on the impact of job training to assess results based on observational data. We apply and extend methods developed by Athey, Chetty, and Imbens (2020) that enable researchers to exploit data from RCTs to address potential bias in results based on observational data in a control-function framework. In particular, we exploit the availability of data for a 1994 randomized evaluation of the modern equivalent of the CCC: the federal Job Corps.^21^ We address potential endogeneity of our CCC estimates using two approaches that make use of the JC RCT.

The approaches require the JC to be an externally valid experiment for the CCC, either for the treatment effect directly or, more realistically, for the degree of selection bias. We first present comparisons of the CCC and JC (limiting the JC sample to men only). Then we proceed to describe our two approaches to correct for bias in the CCC estimates in greater detail and present our bias-corrected estimates. Finally, we quantify how relaxing the assumptions changes our estimates and discuss other limitations of this approach.

#### 1. Comparing CCC and JC Enrollees

Overall, JC and CCC participants are similar along important dimensions (Online Appendix Table A.XVIII and Table A.XIX). Both are young (19 years old on average) and have relatively few years of schooling. JC participants have completed 10.2 years of schooling, compared with 8.6 for the CCC enrollees, and 19% have graduated from high school compared with 12% of the CCC enrollees. The CCC sample has considerably more Hispanic enrollees, due to the fact we concentrate on CO and NM, whereas the JC data are national.

Participants in the JC and CCC are also similar in terms of duration of enrollment and reasons for leaving. Mean duration is 9.8 months (std. dev. 7.47) for CCC and 5.8 months (std. dev. 6.6) for JC. The main reason for the lower duration of the JC participants is that 20% never trained at all. Conditional on training, the duration among the treated group in JC is 7.8 months, similar to the CCC. Reasons for leaving are also similar across the two programs (Figure II).^22^ Finally, and perhaps most important, when we try to predict duration in the JC, we also find evidence of both positive and negative selection into duration. Specifically, we find that on average in both samples (i) individuals who have ever worked previously serve shorter durations, (ii) Hispanic enrollees serve longer, and (iii) individuals with more years of schooling in CCC and with high school or GED degree in JC serve longer (Figure III).

Moreover, the JC and CCC enrollees are both disadvantaged relative to the general population at the time. For example, comparing the average education of the JC participants to men aged 16–24 in the 1990 census, JC participants have 1.8 fewer years of schooling, or 15% lower than the men 16–24 in the census (Table II). Comparing CCC enrollees to men in the 1940 census, they have completed 1.2 fewer years of schooling (13% lower). This pattern is true for other measures, such as household income and employment. In the JC sample, 81% come from households with income below the 41st percentile; in the CCC, 63% do. JC and CCC men are also significantly more likely to be unemployed than the population of young men at the time—for the JC, they are six times more likely to be unemployed, and for the CCC, they are nearly five times more likely.

Even though CCC and JC samples are relatively disadvantaged, to make the samples even more comparable, we construct a set of weights using the entropy balance approach in Hainmueller (2012) using the relative characteristics calculated for each sample. The results based on the reweighted JC sample using the calculated weights make the relative disadvantage between JC and CCC data even more similar, resulting in almost identical standard deviation differences in education, unemployment, and imputed Hispanic share (Online Appendix Table A.XIX). We use both the unweighted and reweighted JC sample in our subsequent analyses.

#### 2. Comparing Short-Term Effects

We reproduce the short-run JC randomized evaluation results in Schochet, Burghardt, and McConnell (2008) using the sample of males and examine additional outcomes. The results are very similar (Table VII). In the first column, we present estimates that compare the outcomes of those assigned to treatment to those of the control (the reduced-form or ITT effect). In the second column, we present the implied effects of training duration by estimating the 2SLS effect of duration using the randomized treatment status as an instrument. The 2SLS estimates represent a rescaling of the ITT estimates in the first column, obtained by dividing the ITT by the mean effect of the program on duration (0.487 years from Online Appendix Table A.XVIII). In the third column, we present 2SLS estimates using the reweighted JC sample, which is more comparable to the CCC sample in terms of relative disadvantage.

**Table VII:** Comparing the CCC Effects to the JC Effects

	Jobs Corps data					CCC
	Randomized controlled trial			OLS	OLS, weighted	OLS
	Coefficient on treatment dummy (ITT)	2SLS instrument duration with treatment	2SLS, weighted	Coefficient on duration (years)^a^	Coefficient on duration (years)^a^	Coefficient on duration (years)
	(1)	(2)	(3)	(4)	(5)	(6)
Years of schooling	0.184***	0.393***	0.320**	0.360***	0.333***	0.169***
	(0.039)	(0.084)	(0.130)	(0.041)	(0.075)	(0.040)
Observations	6,280	6,280	6,013	3,407	3,341	9,620
Employment (in week of the survey)^b^	0.026**	0.056**	0.088**	0.060***	0.085***	−0.015
	(0.013)	(0.027)	(0.040)	(0.015)	(0.020)	(0.022)
Observations	6,022	6,022	5,782	3,285	3,227	3,684
Weeks worked in previous year	1.615***	3.443***	3.635**	2.629***	3.025***	0.265
	(0.536)	(1.142)	(1.741)	(0.610)	(0.718)	(1.199)
Observations	6,235	6,235	5,971	3,382	3,316	2,360
Total annual earnings in previous year	969.765***	2,083.466***	1,999.766*	1,055.435***	509.052	−14.497
	(280.804)	(603.598)	(1,041.844)	(336.311)	(576.113)	(26.389)
Observations	6,081	6,081	5,835	3,317	3,257	2,148
ln(earnings) | weeks worked > 0	0.038	0.080	0.126	0.078**	0.027	−0.014
	(0.027)	(0.057)	(0.103)	(0.031)	(0.055)	(0.062)
Observations	5,009	5,009	4,805	2,753	2,698	1,749
Moved^c^	0.018*	0.038*	−0.017	0.060***	0.028	0.057***
	(0.011)	(0.023)	(0.036)	(0.014)	(0.021)	(0.011)
Observations	6,301	6,301	6,032	3,419	3,348	9,568
Self-reported health excellent or good	0.035***	0.073***		0.020*		
(12-month)^d^	(0.009)	(0.020)		(0.010)		
Observations	5,920	5,920		3,234		
Self-reported health excellent or good	0.016*	0.034*		0.013		
(48-month)^d^	(0.010)	(0.020)		(0.011)		
Observations	6,279	6,279		3,407		
Duration of training in months	7.8					
Individual controls?	No	No		Yes	Yes	Yes

*Notes*. Standard errors clustered at the level of county-by-year-quarter of enlistment for CCC and site-by-year-quarter for Jobs Corps are reported in parentheses. The JC sample includes only men. *** *p* < .01, ** *p* < .05, * *p* < .1.

^a^The sample includes all treated, including those with zero duration. Controls include year and quarter of baseline, year and quarter of 48-month follow-up survey, whether an individual was enrolled in nonresidential program, and baseline characteristics such as whether an individual had children, was ever arrested, had ever used drugs, had a job, had a job in the previous year, ever had a job, race, native language, was on welfare as a child, education, baseline marital status, and others.

^b^Employment is not conditional on labor force participation.

^c^For Job Corps, “moved” is defined as living more than 20 miles away from the baseline residence. For CCC it is defined as living in a different county than the county of residence at the time of enrollment. For JC, employment is defined as having a job during the 208th week after the baseline survey (four years). Earnings conditional on employment only includes the earnings of individuals employed during the 208th week after the baseline survey.

^d^Constructed variable that is equal to one if self-reported health status is 1 or 2 (excellent health or good health).

These estimates represent the causal effect of duration (under standard assumptions).^23^ Columns (4)–(6) show OLS estimates for duration for JC (unweighted), duration for JC (reweighted), and for CCC, respectively. Overall, short-term labor market outcomes in JC were more positive than in CCC where the effects were insignificant. In both the CCC and the JC data, education and mobility increased significantly. The table also shows that the JC increased self-reported health, consistent with our findings that CCC improved health, measured by height and weight.

#### 3. Comparing Longer-Term Effects

The estimated longer-run effects of the CCC on income and disability are also similar to estimates obtained from the JC RCT. Given that enrollees participated for 0.83 years on average, the effect of CCC on lifetime earnings is 4.3%. The latest evaluation of JC, which tracks individual tax records 20 years after the program (Schochet 2021), finds that participation in JC had a statistically insignificant increase in wages of 2.3%, with the CCC effects (4.6) well within their 95% confidence interval [−4.1%; 8.8%]. The evaluation also reports a 40% reduction in SSDI benefits among older JC participants, though not in the overall sample.^24^

In sum, participants in JC are similar in many dimensions to CCC participants and they experienced qualitatively similar long-run (20-year) improvements in income and health. They experienced similar improvements in their education, health, and mobility in the short run, but they differed in the short-term labor market outcomes. We now formally describe our method of using short-run, experimental estimates from JC to adjust lifetime, observational estimates from the CCC.

### VIII.B. Combining Experimental and Observational Samples: Set-Up

We pursue two approaches that exploit experimental data from the JC to address potential selection bias in the estimates of the long-run impact of the CCC. The first approach assumes that the true short-run causal treatment effect of the JC and the CCC is the same. The second assumes that selection into longer duration in the JC and the CCC is the same, an assumption for which we present supporting evidence.

The setting for both approaches is as follows. We assume the short-term (denoted *ST*) outcome is a linear function of the treatment and observed and unobserved covariates:


\begin{eqnarray*}
y_{iS}^{ST} = \tau _S^{ST}{W_{iS}} + \,\,{X_{iS}}\gamma _S^{ST} + \alpha _{iS}^{ST},
\end{eqnarray*}

where $y_{iS}^{ST}$ is the short-term outcome for individual *i* in sample $S \in \{ {CCC,JC} \}$; ${W_{iS}}$ is duration of training in program (either CCC or JC); $\tau _S^{ST}$ is the short-term treatment effect; ${X_{iS}}$ includes other controls; and $\alpha _{iS}^{ST}$ is the unobserved component (residual), which is possibly correlated with ${W_{is}}$ in the CCC sample and is the source of endogeneity of ${W_{\textit{iCCC}}}$. In JC only, we observe a binary treatment status dummy ${T_i}$, uncorrelated with $\alpha _{iS}^{ST}$ given the experimental nature of the data but correlated with training duration ${W_{iJC}}$. This allows us to correct for the endogeneity of duration using the random assignment as an instrument.

Similarly, for the long-term (LT) outcomes, we have:


\begin{eqnarray*}
y_{iS}^{LT} = \tau _S^{LT}{W_{iS}} + {X_{iS}}\gamma _S^{LT} + \alpha _{iS}^{LT}.
\end{eqnarray*}

Going forward we make the following two assumptions:

Assumption 1.
$\alpha _{iJC}^{ST} \bot {W_{iJC}}|{X_i},{T_i}$ in JC (duration is random given treatment status and *X*).

Assumption 2. LT and ST residuals are linearly related as:

\begin{eqnarray*}
\alpha _{\textit{iCCC}}^{LT} = \delta \alpha _{\textit{iCCC}}^{ST} + \varepsilon _{\textit{iCCC}}^{LT}\,\,{\mathrm{and}}\,\,\varepsilon _{\textit{iCCC}}^{LT} \bot {W_{\textit{iCCC}}}|{X_i},\alpha _{\textit{iCCC}}^{ST}.
\end{eqnarray*}

Assumption 2 is the key assumption. It requires that the source of the endogeneity on duration for the short-term and long-term outcomes in the CCC sample comes from a common, unobserved component. If this component can be recovered, we can simply control for it in our analysis of the long-term outcome and obtain unbiased estimates of the treatment effect. Although it is not observed directly, we can recover it from the short-term outcomes if we can obtain an unbiased estimate of the short-term treatment effect, $\tau _{CCC}^{ST}$. The two approaches differ in how we arrive at the unbiased estimate of $\tau _{CCC}^{ST}$.

### VIII.C. First Approach: Assuming Identical Short-Run Treatment Effects for JC and CCC

For our first approach, we follow Athey, Chetty, and Imbens (2020) in assuming that the experimental sample has external validity and therefore $\tau _{CCC}^{ST} = \tau _{JC}^{ST}$: the ST treatment effect obtained from the RCT of JC is an unbiased estimate of the ST effect in the CCC.

We make additional assumptions to account for the differences in settings. First, in Athey, Chetty, and Imbens (2020) the treatment is binary, whereas in our case it is continuous. This makes the assumption of exogeneity of treatment in the ST sample stronger here than in the binary treatment case. Secondly the experiment does not yield direct estimates of the effect of duration, so we use 2SLS to obtain them. As a result, we need to make additional assumptions. In particular, the standard IV assumptions (relevance and exclusion restriction) must hold. See Online Appendix C for details.

After obtaining an unbiased estimate of the JC's short-term effects ($\hat \tau _{JC}^{ST}$) using IV methods, we calculate the short-term CCC residuals, $\hat \alpha _{\textit{iCCC}}^{ST} = Y_{\textit{iCCC}}^{ST} - \hat \gamma {X_{\textit{iCCC}}} - \hat \tau _{JC}^{ST}{W_{\textit{iCCC}}}$, and include the residuals as control functions in the long-term regressions. If Assumption 2 holds and JC's short-term effect is an unbiased estimate of the CCC's short-term effect (so that $\hat \alpha _{\textit{iCCC}}^{ST}$ consistently estimates $\alpha _{\textit{iCCC}}^{ST}$), then the coefficient on duration estimated including the control functions gives us the LT causal effect of the CCC.

The control-function estimates for our long-run outcomes are presented in Table VIII.^25^ Note that these residuals can only be computed for the sample of participants for whom we observe both short-term and lifetime outcomes. For comparison, we present OLS results for the same sample in Panel A—reassuringly, despite the sample size reductions, these estimates are very similar to our main estimates. We report the control-function results using both the unweighted and reweighted JC sample in Panel B. The LT estimates of the effect of duration on our long-term outcomes are unaffected by the inclusion of control functions for all samples and specifications, including the specification using the reweighted JC sample to compute the control function. This again suggests that any bias in the OLS estimates is small.

**Table VIII:** Long-Term Estimates of the Effects of CCC Using Education Control Functions for Identification

Dependent variable	Log death age	AIME	Retirement or disability claiming age	SSDI
	(1)	(2)	(3)	(4)
Panel A: OLS without control functions				
Duration of service (years)	0.013***	47.882**	0.509***	−0.02
	(0.004)	(21.416)	(0.189)	(0.014)
Panel B: Control function approach 1 (Athey et al. 2020)				
Unweighted				
Duration of service (years)	0.013***	52.363**	0.418***	−0.022
	(0.004)	(21.640)	(0.142)	(0.014)
Bounds to account for assumption violations^a^	±7.22E-05	±1.152	±6.14E-03	±5.78E-04
Reweighted				
Duration of service (years)	0.013***	50.230**	0.407***	−0.021
	(0.004)	(21.512)	(0.141)	(0.014)
Bounds to account for assumption violations^a^	±1.34E-04	±2.13E+00	±1.14E-02	±1.07E-03
Panel C: Control function approach 2 (this article)				
Unweighted				
Duration of service (years)	0.013***	46.809**	0.388***	−0.019
	(0.004)	(21.391)	(0.142)	(0.014)
Bounds to account for assumption violations^a^	±5.75E-07	±0.000	±3.66E-07	±5.96E-07
Reweighted				
Duration of service (years)	0.013***	45.816**	0.382***	−0.019
	(0.004)	(21.377)	(0.142)	(0.014)
Bounds to account for assumption violations^a^	±9.31E-05	±1.39E+00	±8.34E-03	±7.32E-04
Observations	7,722	4,613	4,575	4,575

*Notes*. Standard errors clustered at the level of county-by-year-quarter of enlistment are reported in parentheses. This table reports the coeffcients on duration in a regression of log age at death, average indexed monthly earnings (AIME), retirement or disability claiming age, and SSDI claiming. The sample includes enrollees for which the control function using education can be computed using only common covariates between JC and CCC (enrollment age, age less than 18 indicator, highest grade level, Hispanic status, whether held a previous job, whether graduated high school, household size, from rural hosehold, whether father is living, whether mother is living). The reweighted sample uses weights calculated using Hainmueller (2012) with relative disadvantages as inputs. See the text for a description about how the control functions in Panels B and C are constructed. *** *p* < .01, ** *p* < .05, * *p* < .1. ^a^This term can be multiplied by desired percentage difference in treatment effect between JC and CCC (Panel B) or omitted variable bias between JC and CCC (Panel C) to calculate the final bounds.

This is not a result of the small treatment effects estimated in the JC trial—the bias correction is always based on the difference between JC RCT and the CCC, not on the treatment effect in JC alone.^26^ Instead, these results can reflect that (i) the LT unobservables are uncorrelated with duration, indicating little endogeneity; (ii) the LT unobservables are uncorrelated with LT outcomes; or (iii) the ST and LT unobservables are different and the controls do not capture the endogeneity. However, the results are similar if we use other ST outcomes to compute the control functions or include different sets of controls making (iii) unlikely (Online Appendix Table A.XXI).

We consider these results encouraging, but they rely on an assumption that ST treatment effects in JC would be identical in the CCC, after adjusting for some covariates. This assumption seems reasonable for education or geographic mobility, but less so for labor market outcomes. The returns to training, like the returns to other human capital investments, are likely to depend on post-investment market conditions. There is evidence that the returns to schooling are stochastic and vary considerably over time (Goldin and Katz 2008; Rosenzweig and Udry 2020). Labor demand for low-skilled laborers after World War II was stronger than it was in the economy of the early 2000s, which had stagnant wages for low-income groups (Piketty, Saez, and Zucman 2018) and low-skill workers (Autor, Katz, and Kearney 2008). This suggests that the assumption of constant treatment effects over time and place might be too restrictive for labor market outcomes. In the next section we consider a second approach that relies on what we argue is a more realistic assumption: that selection into longer job-training duration is similar across settings.

### VIII.D. Second Approach: Assuming Equivalent Selection into Longer Duration in JC and CCC

For our second control-function approach, we allow for the possibility that the short-term treatment effects are not the same in JC and CCC. Instead, we assume that the selection bias into longer training duration is the same in JC and CCC. Our starting point is the assumption that the two samples share key features, most importantly that the choice in the observational CCC sample to participate in the program depends on the same unobserved components as the decision to comply with the assignment to the program in the JC experiment. If we view this decision in both cases as motivated by individual-specific preferences, it is plausible that these unobserved components are closely related.

In our case and based on economic theory we can hypothesize that the more educated will have greater outside options (more offers in the labor market), which would lead them to stay in the training program for shorter periods of time. Poor individuals will instead likely benefit more from the income that the program provides, leading them to remain in the program for longer. Both of these considerations would apply in the CCC and in the RCT.

Put another way, the key assumption is that the bias in the OLS estimates in the JC sample, as estimated by the difference between the JC's OLS and 2SLS estimates, is the same as the bias in the OLS estimates in the CCC sample. Formally, this requires an additional linearity assumption, namely, that $\hat \mu = \hat \tau _{JC,OLS}^{ST} - \hat \tau _{JC,2SLS}^{ST}$ is a consistent estimator of the selection bias in both the CCC and JC.

We argue that those who stayed in the program for extended periods of time likely had similar motives. The following set of facts support this assumption: (i) the mean duration and the distribution of reasons for dismissal are similar, (ii) the observables predict duration in a similar fashion, and (iii) the enrollees in the JC and CCC samples exhibit similar patterns in observable characteristics and relative disadvantage compared to their contemporary peers.

We estimate the selection bias by taking the difference between the OLS and IV estimates from JC: $\hat \mu = \hat \tau _{JC,OLS}^{ST} - \hat \tau _{JC,2SLS}^{ST}$. The estimate represents the omitted variable or selection bias in JC, which in turn is an estimate of the CCC selection bias under the above assumption. We use our adjusted OLS estimate of CCC short-term outcomes ($\hat \tau _{CCC}^{ST} = \hat \tau _{CCC,OLS}^{ST} - \hat \mu $) to calculate the control functions as before. That is, we use this adjusted estimate of the short-run treatment effect of the CCC to calculate a residual in regressions of short-run outcomes. See Online Appendix C for the detailed procedure.

We present the results for the long-run outcomes using the control function calculated from our short-run education outcome (Table VIII, Panel C). Again, we find that regardless of the control functions we include and whether we reweight to make samples comparable in terms of relative disadvantage, the estimates are similar to the original OLS estimates, suggesting little bias in our LT estimates of the effects of training (Online Appendix Table A.XXII).

### VIII.E. Limitations

Athey, Chetty, and Imbens (2020) present nonparametric identification results, with key assumptions including a restriction on the dimension of the unobserved selection component and monotonicity of the outcome in these unobserved components. In our application, we extend this by estimating effects of interest using IV, and in doing so we augment their assumptions with conventional IV assumptions, which include some parametric components. In addition, in the second approach, we assume that the bias resulting from selection is additively linear, a standard formulation of the omitted variable bias.^27^

We can quantify how violations of some of the assumptions in each approach change the estimated coefficient of the long-run effect. Both approaches rely on obtaining an unbiased estimate of the short-run treatment effect in the CCC. In both cases, define $\phi = $$( {\hat \tau _{CCC}^{ST} - \tau _{CCC}^{ST}} )$, the difference between our “recovered” short-run estimate and the true treatment effect. Any $\phi \ne 0$ leads to a bias of $- \delta \times \phi$ in the estimate of the long-run treatment effect with control functions generated from our approaches. For the first approach, $\phi $ is the difference between JC and CCC short-run treatment effects. In the second approach, $\phi $ stems from the difference in the estimate of the selection bias in the short-run regression for JC and CCC. In Online Appendix C, we fully quantify how the bias relates to percentage differences (i) in the treatment effects between JC and CCC in the first approach, or (ii) in the correlation between duration and the unobserved component in the short-run regression in the second approach. This allows us to examine how much our estimate of the long-run effect would differ if we were to assume various degrees of violation in our key assumptions.

We present the “bias” terms as symmetric bounds around the estimate in the last rows of Table VIII, Panels B and C. These bias terms can be multiplied by a desired amount of the relevant percentage difference in either approach. We see that because the bias terms are extremely small, only very large differences in either the short-run treatment effects between JC and CCC or selection bias between JC and CCC would make a meaningful difference in the estimates. We take this as additional evidence that our long-run estimates are robust to different kinds of adjustments for omitted variable bias.

Finally, we have so far assumed that the long-run treatment effects are homogeneous. However, we can allow for some forms of treatment effect heterogeneity (see Online Appendix C for more details).^28^ To investigate the presence of heterogeneity in the long-term outcomes, we estimated models where the control function is interacted with the duration. We report the average treatment effect (ATE) from these specifications in Online Appendix Tables A.XXIII and A.XXIV. We find that the ATEs that allow for heterogeneity are very similar to our main effects.

We can also account for unobserved heterogeneity in the short-term outcomes by exploiting the presence of multiple short-term outcomes so that we can recover two (or more) residuals that affect the primary outcome. We can then simultaneously control for and/or interact these multiple control functions with the duration in the long-term equation. These results are shown in Panels C and D of Online Appendix Tables A.XXI and A.XXII (not interacted) and Tables A.XXIII and A.XXIV (interacted), and again our estimate of the ATE remains unchanged. Adding control functions based on multiple short-term outcomes strengthens confidence that our estimates are not biased because they are more likely to capture different dimensions of the unobservables in the error term. We conclude that our estimates of the long-term effects of the program are largely unbiased.

## IX. Discussion

The CCC was a comprehensive paid employment and training program for unemployed youth during the deepest economic recession in U.S. history. While existing work has examined the effect of New Deal programs on aggregate economic outcomes, this represents the first effort to estimate lifetime impacts on program participants. We find that the program benefited participants over their entire lifetimes.

What broader lessons does this historical evaluation offer? We conclude by discussing two ways of thinking about the value of this program and other lessons that are generalizable to the present.

First, we consider whether the program fully compensated the participants for the losses they suffered as a result of the Great Depression. Similar to other New Deal Programs, the CCC was intended to help recipients during a severe economic crisis. Did the program succeed? To answer this, we make use of the findings from Schwandt and von Wachter (2020), who investigated the impact of the 1982 recession on longevity and earnings to estimate the cost of recessions over a lifetime. They report that a change in the unemployment rate of 3.9 percentage points lowered longevity of young adults entering the labor market by 6–9 months and find that a 1% increase in the unemployment rate at the time of entry into the labor market lowers middle age incomes by 1.3%.

Our results suggest that those who participated in the program for 1 more year lived 0.96 years longer (CI 0.5–1.4) and their earnings were 5.2% higher (CI 2.3–8.1). While these benefits are substantial, they suggest that the program may not in fact have fully compensated affected youth. Unemployment rates during the Great Depression have been estimated to have risen to a high of 25 percentage points, from a pre-Depression baseline of around 5 or less.^29^ If the Great Depression increased unemployment by 20 percentage points, we would predict an average loss of lifetime income of 26% and a decline in longevity of 3.4 years.^30^ Assuming linearity and using the upper bounds for our estimates, only those that participated in CCC for at least 3.5 years, far greater than what was allowed, would have been fully compensated.

However, modern recessions have resulted in much smaller increases in unemployment, suggesting that such a program would compensate enrollees. For example, these gains would completely offset the losses of youth entering the labor market in the 1982 recession. Thus, paid employment programs may serve as a way to protect youth who do not qualify for countercyclical programs like Unemployment Insurance.

An alternative way to assess the value of this program is to investigate if it was worth the cost, and in particular, to determine how our ability to estimate long-term gains changes our assessment of a program's value. To answer this question, we calculate the MVPF following the approach by Hendren and Sprung-Keyser (2020).^31^ It's worth noting that the MVPF we compute based on our results alone likely misses additional benefits and certain costs. The program likely benefited not just enrollees but also their families—parents, siblings, and potentially extended family (Kugler et al. 2015) and children—and the communities and the landscape where the CCC operated. Our calculations also miss potential negative spillover and general equilibrium effects of such a large labor-mobilization program. Though we think spillovers are likely minimal in the context of the CCC because of the unusually high unemployment at the time and the placement of CCC camps far from existing labor markets, one should still consider them (Crépon et al. 2013).

We estimate an MVPF of 6.0 when we include the willingness to pay for increases in longevity, disability reductions, and increases in claiming ages, well above 1. If we only include earnings gains and exclude mortality reductions, the MVPF is 2.5, still significantly greater than 1, but below 6.^32^ If we used the short-term effects only, the MVPF would be below 1. This contrasts with the MVPF of less than 1 calculated for JC participants in Hendren and Sprung-Keyser (2020).^33^ The key difference is our ability to look at lifetime effects on multiple outcomes, particularly health and longevity.

These findings suggest that it is essential to evaluate multiple mechanisms and indicators of well-being when assessing the effects of various interventions in the short and the long term. In our case, the ability to look at multiple outcomes over the long run, particularly our ability to consider health effects, vastly increases the assessed value of the program. However, these benefits are hard to assess in the short term—we do not find any economically or statistically significant impacts on survival until after age 55 and only find effects on income over the long run. Overall, our findings confirm Krueger's view in Heckman and Krueger (2005) that the benefits of training are similar to the benefits of education, and like education, the resulting benefits may only manifest over the long term. An important question for future research is whether there exist short-term markers that can be used to predict these long-term gains. If so, this would provide policy makers, who are often constrained by short-term budget and other considerations, with better evidence regarding likely lifetime benefits.

## Supplementary Material

qjae016_Online_Appendix

## Data Availability

The data underlying this article are available in the Harvard Dataverse, https://doi.org/10.7910/DVN/A9YQTL (Aizer et al. 2024).

## Footnotes

1. Recent work shows some youth programs can have sustained benefits over the short and medium term. For example, Katz et al. (2022) document gains in earnings of sectoral employment programs.

2. Although Schochet (2021) analyzed medium-term outcomes in the 20-year follow-up of Job Corps, he did not measure lifetime impacts. Our study is the longest follow-up of a training program of which we are aware.

3. For example, in 1939 and 1940, about 52% of CCC enrollees had eight years of schooling or less (Federal Security Agency 1940).

4. New Mexico also had a large share of Native Americans. Native Americans had their own CCC programs, which operated separately within reservations and were administered by the Bureau of Indian Affairs. See Parman (1971) for details. We have no data from this program.

5. We could not construct a suitable control group of individuals that did not serve in the CCC. In assessing the effects of training programs using observational data, Lalonde (1986) and Smith and Todd (2005) emphasized the need for “a rich set of variables related to program participation and labor market outcomes,” to create suitable control groups, and also that “the nonexperimental comparison group be drawn from the same local labor markets as the participants.” In the absence of these rich controls, observational estimates tend to deliver negative treatment effects.

6. We established based on published reports from the CCC that the records account for the complete population of records starting in 1937 (see Online Appendix Figure A.II).

7. Our match rates are higher than those typically found in the literature (which range from 20% to 50%, see Bailey et al. 2020, Abramitzky et al. 2021) for two reasons. First, administrative records contain information not just on individuals but also on their family members. Second, the death records come from various sources, not just the DMF, including state vital registration sources, deaths during World War II, and gravestones. However, mortality information is still missing for some individuals for several reasons. First, some died before 1975, which is the first year of complete death records in the DMF (Hill and Rosenwaike 2001), though we can still find a death record for them in state vital records or gravestone databases. Second, based on SSA life tables we compute that about 1.1% of people born in 1920 (our median birth year) would be expected to be alive by 2017.

8. We only observe an SSN if the person reported it in the application in CO, or if it is available in the death certificate. However, SSNs are not available for anyone who died after 2008 (these are masked for privacy reasons) or for those who died young and never applied for a Social Security card.

9. We compare the means in our estimation sample to the published national means. These were published in Federal Security Agency (1937), Appendix H: “Census of Civilian Conservation Corps Enrollees.”

10. Alternatively, we can fix the polynomial approximation and smoothness at one and let the Cattaneo et al. (2024) algorithm choose the optimal bandwidth (Online Appendix Figure A.V). The results are robust between these two specifications.

11. These characteristics include: ever rejected from the CCC, disabled, nonjunior member, age, dollars per month allotted, gap in service, distance from the camp to home, whether Hispanic; for those in CO only, highest grade completed, household size, live on a farm, height and weight at enlistment, whether mother or father was deceased, tenure in the county prior to CCC enrollment, and reason for discharge. For missing data, we impute using the mean and include a series of indicators to denote imputation. The full regression with coefficients for controls is in Online Appendix Table A.V. We also report alternative standard errors in Online Appendix Table A.VI from clustering on county or year-quarter of enlistment instead of CQE, which make no material difference.

12.
Table III reports Oster bounds for the specification used in column (6), with individual and peer characteristics and camp fixed effects.

13. Consistent with this, we observe in the data that this reason for dismissal is much more common toward the end of the program in 1942 (27%) than in 1938 (1.8%). We also find that the fraction dismissed for COG is 21% among those who are sent to a camp that closes within a year, compared with only 12% of those assigned to camps that close later.

14. We present the specification that includes camp peer characteristics (as in Table III, column (5)) but not camp × enrollment fixed effects due to little variation in COG within camp and enrollment year-quarter. The IV estimates for all specifications are presented in Online Appendix Table A.VIII, and the results are mostly consistent across specifications.

15. Recent work by Lee et al. (2023) shows that if the *F*-statistics are lower than 104.5, then 95% CI need to be adjusted. This adjustment is very small in our case and does not materially change our conclusions.

16. We do have another, slightly noisier, proxy of earnings: the primary insurance amount (PIA), which is based on the AIME, that is available prior to 1979 as well. We find similar results for the PIA pre- and post-1979. See Online Appendix Table A.XII for results.

17. Note that when we exclude controls for birth cohort, there is no relationship between training duration and lifetime earnings. This is because more recently born cohorts had shorter durations due to the end of the program but higher incomes, due to secular trends in earnings, generating a spurious negative correlation.

18. Duration does not predict whether we find an enrollee in the 1940 census once we include birth cohort and county-quarter fixed effects (Table V, column (1)).

19. When we restrict our analysis to those with nonmissing baseline education, the estimate declines to 0.12 and remains significant at the 1% level (Online Appendix Table A.XV, column (7)).

20. In the 1940 census, 12% of people report living in a different county than in 1935. See https://www.census.gov/dataviz/visualizations/010/.

21. The current website (https://www.doleta.gov/job_corps/) states that “the program helps eligible young people ages 16 through 24 complete their high school education, trains them for meaningful careers, and assists them with obtaining employment... Students can earn a high school diploma or the equivalent, and college credits. Job Corps also offers tuition-free housing, meals, basic health care, a living allowance, and career transition assistance.”

22. About 30% of JC enrollees complete the program, compared with 38% of the CCC. Of those who leave before completing, 30% in the JC and 22% in the CCC “deserted,” while 12% and 4%, respectively, left because of employment opportunities.

23. The key assumption is that there is no direct effect of the assignment to treatment on the outcome beyond its effect on the duration of the training. Online Appendix Table A.XX presents balance tests of baseline characteristics of the JC applicants.

24. We calculate the numbers using the treatment-on-the-treated (TOT) effect of W2 earnings at 2015 (Schochet 2021, tables 2 and A2). The JC evaluation only uses 15 years of labor market outcomes, whereas we use 35 years. The shorter length of the evaluation may lower the estimated returns.

25. We present the specification based on education as our ST outcome, which maximizes our sample size, in the main tables. However, we consider multiple short-run residuals (since we have multiple short-run outcomes), and our results are robust to their inclusion.

26. The bias correction is also based on the relationship of two different sets of outcomes (ST and LT) in CCC, not on the treatment effect in JC alone. As long as ST and LT selection are related within CCC, the process should correct any bias of our estimate. If the ST treatment effect is zero in the RCT, then the control function is the ST outcome, which can still generate a change in the estimated coefficients.

27. Although making such assumptions is unavoidable, we can change some of the assumptions in the implementation of the approach: for example, instead of selection bias being additive, we could assume it is proportional.

28. We cannot allow for completely unrestricted heterogeneity at the individual level. For example, if we allow for an *i* subscript on $\tau _s^{ST}$, treatment-effect heterogeneity will lead the residual from a projection of $Y_{iS}^{ST}$ on ${W_{iS}}$ to exhibit a variance that depends positively on ${( {{W_{iS}} - {{\bar W}_S}} )^2}$. In this case the estimator would no longer be consistent for the average effect. Endogenous heteroskedasticity of this nature is a common problem in many empirical applications. If the heterogeneity is limited, one would expect the biases to be modest.

29. Unemployment rates are not well measured prior to 1940; see Card (2011).

30. The computation for income assumes that middle age income is a good proxy for lifetime income. Prior literature finds support for this assumption (Chetty et al. 2014).

31. The main approach consists of estimating the present discounted value of cost and benefits of the program, including their effects on government revenues (for details see Online Appendix D). The CCC program costs include the up-front cost of the program estimated by Levine (2010) to be $1,004 per enrollee per year in 1939 dollars ($14,384 in 2017 dollars) and increases in Social Security payouts from enrollees both living longer and having increased PIA. These costs are mitigated by (i) the tax increases of $6,965, resulting from the increased earnings, which we calculate by applying a tax rate of 33.6% to the AIME; (ii) a decrease in SSDI payout of $911 resulting from lower claiming rates and the increase in claiming age; and (iii) a decrease in Social Security payout of $733 resulting from the increase in retirement age. In addition, the program benefits include (iv) the willingness to pay for the increase in survival rates, which we value using a statistical value of a year of life of $150,000, a middle-range estimate across studies (Keller et al. 2021); (v) an increase in after-tax earnings of $13,642, which is simply the posttax portion of pretax income gains; (vi) the monthly real wage of $66.25 recipients obtained while enrolled (BLS 1941), which includes benefits such as room and board, multiplied by average duration and computed in 2017 dollars, $11,390; and finally, (vii) the decrease in benefit from loss of SSDI income of $911.

32. Some of the increases in life expectancy could lead to greater government spending through Medicare, potentially lowering the marginal value of public funds (Hendren and Sprung-Keyser 2020)—we ignore these. Families received transfers, which could have benefited them but also potentially distorted their behaviors.

33. They compute an MVFP of 0.18 for JC. This computation does not incorporate the lower SSDI claims or the potential life extensions we compute here.

## Notes

We thank Joe Price and the BYU Record Linking Lab for helping us collect the data for this project. Barbara Smith at the Social Security Administration provided invaluable contributions. We thank many research assistants that worked on this project, especially Ryan Boone, Taehoon Kang, and Kyle Sherman. We have benefited from comments from participants in the various conferences. We particularly thank Rodrigo Pinto for many valuable contributions. We also thank Nathaniel Hendren for his careful look at the marginal value of public funds computations. Finally, we thank Carlos Flores and Alfonso Flores-Lagunes for their extensive help with Job Corps data and related materials. This research was funded by the Social Science and Humanities Research Council of Canada and by the Social Security Administration Grant no. NB17-16. This research was also supported by the U.S. Social Security Administration through grant no. 5-RRC08098400-10 to the National Bureau of Economic Research as part of the SSA Retirement Research Consortium. This project was additionally supported by the California Center for Population Research at UCLA, which receives core support (P2C-HD041022) from the Eunice Kennedy Shriver National Institute of Child Health and Human Development. Finally, this material is based on work supported by the National Science Foundation Graduate Research Fellowship under grant no. DGE-1650604. Any opinions, findings, conclusions, or recommendations expressed in this material are those of the authors and do not necessarily reflect the views of the National Science Foundation, the SSA, any agency of the federal government, the NBER, the Federal Reserve Bank of Philadelphia, or the Federal Reserve System. All errors are our own.
