# Supplementary material for: The Lifetime Impacts of the New Deal's Youth Employment Program
Source: Q J Econ. 2024 Jun 8;139(4):2579–635. doi: 10.1093/qje/qjae016 (PMC11461809; doi:10.1093/qje/qjae016)
Supplement: qjae016_Online_Appendix [file qjae016_online_appendix.pdf]

Online Appendix for

THE LIFETIME IMPACTS OF THE NEW DEAL'S YOUTH EMPLOYMENT PROGRAM

Anna Aizer
Nancy Early
Shari Eli
Guido Imbens
Keyoung Lee
Adriana Lleras-Muney
Alexander Strand

For online publication only

Online Appendix
Table of Contents

A. Overview of data sources and processes
1. CCC Archives
2. CCC Camps
3. Colorado Name Index
4. FamilySearch (BYU Record Linking Lab)
5. Social Security Administration
6. Assignment of individual ids for multiple records
7. Imputing camp numbers for CO data
8. Construction of camp location and characteristics from historical records
9. Imputing Hispanic Origin
10. Imputing Probability of Survival

B. Matching Individuals to 1940 Census and WWII Enlistment Records
1. Introduction to Matching Approach
2. Overview of Matching Procedure
3. Implementation
4. Census Matching Results
5. WW2 Matching Results

C. Control Function Approach
1. Athey Chetty Imbens (2020)
2. ACI Linear Setting and Our Approach
3. Quantifying the Effect of Violations of Assumptions
4. Heterogenous Treatment Effects

D. Calculation of Marginal Value of Public Funds

E. CCC Regulations

F. Special Acknowledgements

G. Appendix Figures

H. Appendix Tables

A. Overview of data sources and processes

Data used for CCC is assembled from various sources. The major sources of data are:

1) **Archival documents** that include application and discharge forms newly digitized by us and various information about CCC camps of primarily New Mexico and Colorado.
2) **FamilySearch / Ancestry.com** data that links the individuals found in the archival files to various historical sources available online from familysearch.com and ancestry.com, assembled by the BYU Record Linking Lab
3) **Social Security Administration Death Master File** data where we use the SSN, death date, and birth dates found in (2) to link people to correct identifiers

These sources are combined to create the final record-level data. Because some records in the archive belong to the same individual, the record-level data contain more observations than the number of individuals. We tag records so that records belonging to the same individual are assigned the same *PersonID*. We detail the procedure in Section 2.

We use the person-level data and add in additional sources of data to complete the Analysis Sample. The records we link are:

1) **1940 Census** that we machine-match for demographic and family characteristic variables
2) **WWII enlistment records** that we machine-match for demographic variables

The individuals in the Analysis Sample are uniquely identified by variables *state* (of enrollment) and *PersonID*. This is the final dataset used for analysis.

More details on each section:

1. CCC Archives

Colorado (CO)
The Colorado data is from transcriptions of following records: (i) *Certificate of Selection for the Civilian Conservation Corps*, (ii) *Application for the Enrollment*, (iii) *Discharge Form* (Unofficial name). The records are found in the Colorado State Archive under the title "Civilian Conservation Corps Enrollments (Statewide) 1936-1942."

New Mexico (NM)
The New Mexico data is from transcriptions of *Civilian Conservation Corps, New Mexico District records.* (Citation number: collection 1959-030)

2. CCC Camps

The opening and closing dates of CO camps come from Robert W. Audretsch, who supplied us with a list of camps, their associated companies, and the beginning and start dates of the company numbers within the camps.

The CO camp location comes from various historical records that we hand-coded.

The camp type code information comes from http://www.ccclegacy.org/CCC_Camp_Lists.html.

3. Colorado Name Index

Colorado Name Index contains information on a subset of enrollees and their camp assignment that was retrieved from searching through mentions of enrollees' names in contemporary local newspaper articles. Local newspapers often announced young men in their area who enrolled in the CCC and contained basic information about their enrollment. We have used this information to impute camp numbers in cases we were missing them. The procedure is detailed in Section 4.

The *Colorado Name Index* is from the following book:

A Colorado Civilian Conservation Corps Enrollee Name Index
by Robert W. Audretsch
Publisher: CreateSpace Independent Publishing Platform; 1 edition (April 5, 2017)
ISBN-10: 1545102910
ISBN-13: 978-1545102916
Amazon link: https://www.amazon.com/Colorado-Civilian-Conservation-Corps-Enrollee/dp/1545102910

4. FamilySearch (BYU Record Linking Lab)

After the records from the state archives were transcribed and cleaned, individuals in the data were sent to the BYU Record Linking Lab to be found in various historical genealogy websites including Ancestry.com and FamilySearch.org. Their date of death and social security numbers were collected. The individuals' names, date of birth, place of birth, allottee (usually a family member) names were used to find these individuals. The match is performed by trained historians, using records from multiple data sources and information from CCC.

The BYU Record Linking Lab found two major variables:

i. **SSN**
Social security numbers were mostly found on Ancestry.com. The sources of the SSNs on Ancestry are:

1) Ancestry.com. *U.S., Social Security Death Index, 1935-2014* [database on-line]. Provo, UT, USA: Ancestry.com Operations Inc, 2011. Original data: Social Security Administration. *Social Security Death Index, Master File*. Social Security Administration.

2) Ancestry.com. *U.S., Social Security Applications and Claims Index, 1936-2007* [database on-line]. Provo, UT, USA: Ancestry.com Operations, Inc., 2015. Original data: Social Security Applications and Claims, 1936-2007.

Note: SSN is only available for those who have been dead for 10 years. Therefore, we cannot find SSN for those who died before 2005/2006.

For reference, see:
SSDI: http://search.ancestry.com/search/db.aspx?dbid=3693
SSACI: http://search.ancestry.com/search/db.aspx?dbid=60901

ii. **Death Dates**

Death dates were found using various sources including the aforementioned social security administration data, Find A Grave Index, and other sources.

1) Ancestry.com. *U.S., Social Security Death Index, 1935-2014* [database on-line]. Provo, UT, USA: Ancestry.com Operations Inc, 2011. Original data: Social Security Administration. *Social Security Death Index, Master File*. Social Security Administration.

2) Ancestry.com. *U.S., Find A Grave Index, 1600s-Current* [database on-line]. Provo, UT, USA: Ancestry.com Operations, Inc., 2012. Original data: *Find A Grave*. Find A Grave. http://www.findagrave.com/cgi-bin/fg.cgi.

5. Social Security Administration

Finally, we get information on individual's Average Indexed Monthly Earnings (AIME), retirement age, and SSDI claiming behavior by matching our individuals to Social Security Administration's (SSA) Master Beneficiary Record File (MBR).

In order to find our individuals in SSA's MBR, we need the individuals' SSN, first and last names. As described above, for some of our individuals, we have SSN information directly found by BYU Record Linking Lab from various historical sources. For others whose SSNs were not found by the Lab, we use the combination of date of death, date of birth, place of death, first and last names to locate them on the Social Security Death Master File to retrieve their SSNs. The combination of the SSNs and first and last names were used to match these individuals to the SSA's MBR.

6. Assignment of individual ids for multiple records

Individuals can generate multiple records in the CCC record-keeping system. For example, a person who enrolled twice could generate two records: one enrollment form for each time he enrolled. Because our raw data consists of records of enrollment and discharge, our raw data is in the record-level, not in the individual-level. We convert the record-level raw data into an individual-level data by using the information in the records to assign records to unique individuals.

We use the following information in each record to determine whether records belong to the same individual: enrollee's first and last names, birth dates, CCC serial number, social security

number (if available in the original records for CO), allottee's first and last names, and allottee's relation to the participant. All of these fields in each record are subject to transcription and record-keeping errors. In addition, SSN data is only sparsely available for CO enrollees. Therefore, we first use a "fuzzy" matching algorithm for each record to group records with similar field values. Then, we verify the matches manually. Additional information from the BYU Record Linking Lab allowed them to tag more records as coming from the same individuals.

Records vs Individuals Statistics

	CO	NM
Number of Records	21,538	10,713
Number of Individuals	18,644	9,699
Number of Individuals with…		
- 1 record	16,082	8,746
- 2 records	2,263	894
- 3 records	269	57
- 4 records	27	2
- 5 records	3	0

7. Imputing camp numbers for CO data

We have used various sources to impute camp numbers for individuals that do not have camp information in the CO data.

1) **Company Numbers**: For some enrollees, we have company numbers but not camp numbers. The correspondence between company and camp numbers were obtained from Robert W. Audretsch, who documented the company number assigned to specific camps over time.
2) **CCC Serial Numbers**: Each enrollee was assigned a serial number when they first enrolled. The serial number contains the area of enrollment (as described in Section 2) and the company number they were assigned to. The company numbers were then used to impute the camp of assignment.
3) **Colorado Name Index:** For enrollees with enrollment date information but no camp information (either directly from the records or that could be imputed from the serial numbers), we supplemented the camp information through the *Colorado Name Index*. As described in Section 1, the Index contains information from local newspapers on enrollees and their camps at a point in time (when the article was published). We used enrollees' first and last names, place of birth or place of enrollment application, and their enrollment and discharge date to manually match the enrollee to a newspaper record in the Index. Then, we assigned the camp information from the Index as the enrollee's first camp of assignment.

8. Construction of camp location and characteristics from historical records

Camp ID in administrative records merged with camp-information from multiple sources. Dates of operation of camp were obtained from Robert W. Audretsch. Camp location was approximated by location descriptions in historical documents.

a. Map of Colorado's CCC camps

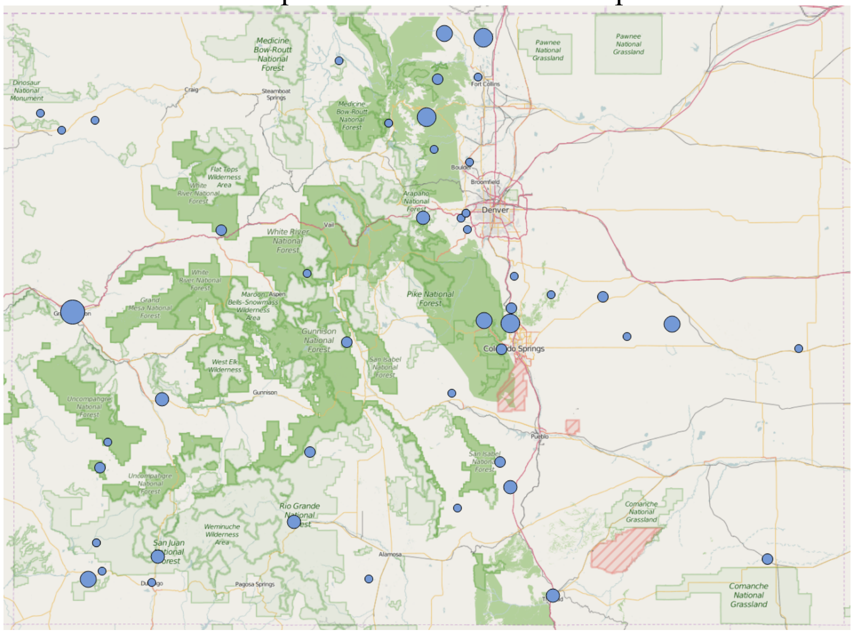

b. Map of New Mexico's CCC camps

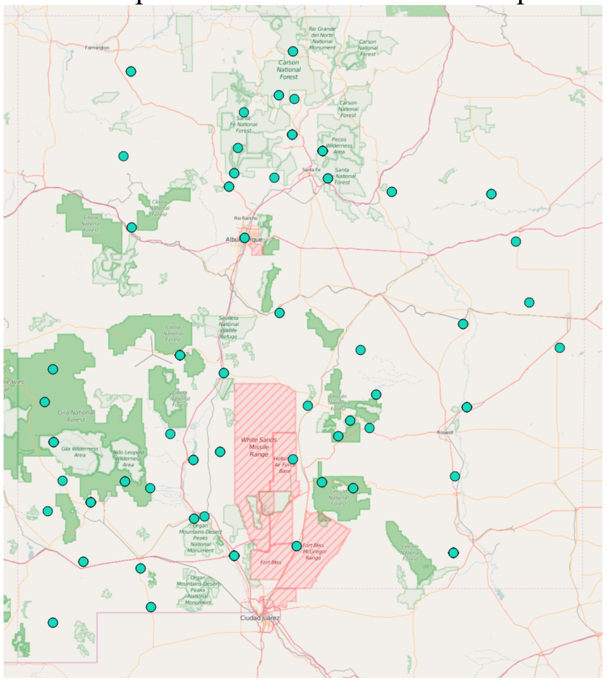

Distance to closest town was computed taking the list of Colorado and New Mexico towns and their latitude and longitude from United States Geological Survey's Geographic Names

Information System (USGS GNIS). Pairwise distances from each camp to each city was calculated, then for each camp, the town with the smallest distance value was selected as the distance to closest town.

Camp weather information was obtained from historical weather data at the PRISM Climate Group at Oregon State University. The data contains minimum and maximum temperature and precipitation at the monthly level and covers the entire United States from 1985-1980 at the spatial scale of 4km x 4km. It is a climatologically aided interpolation and takes as first guess the long-term averages in the area. For more information, visit the PRISM website at http://prism.oregonstate.edu/historical/. We obtain the historical monthly weather data for each camp from the GIS raster files using camp location (longitude and latitude).

Camp peer characteristics are computed using information of individuals at each point in time in our dataset. The peer characteristics for enrollee i is the weighted average of demographic characteristics of other enrollees in our data who served in the same camp overlapped in service duration with i, where the days of overlap are used as the weights. Thus, enrollees that overlapped for a longer period of time get higher weights in the peer characteristics calculation.

In other words, the peer characteristics PX_i of enrollee i is calculated by,

$$PX_i = \sum_{j \in K_i} \frac{d_{ij}}{\sum_{j \in K_i} d_{ij}} x_j$$

where K_i is the set of enrollees that overlap with i, d_{ij} is the days of overlap between i and j, x_j is the demographic characteristic of j.

9. Imputing Hispanic Origin

We follow the approach of Fryer and Levitt (2004) to construct a Hispanic name index for any first or last name using the 1940 Census. The name index is constructed using the Hispanic indicator variable in the 1940 Census. Each first and last name is given a value (0-1) based on:

$$HispIndex_i = \frac{\#\ of\ indiviuals\ with\ name\ who\ are\ Hispanic}{\#\ of\ individuals\ with\ name}$$

Individuals were not directly asked whether they are Hispanic during the Census until 1980 so an algorithm was used to classify individuals in prior Censuses retroactively. Eight rules were used, but at their most basic they are:

1) Individual or their parents/grandparents were born in a Hispanic area
2) Individual has a Spanish surname and was born in the US
3) Individual is a relative or spouse of someone who qualifies by (1) or (2)

Once the indexes are created, they are matched to CCC participants. There is an index for first name, last name, and a combined index created by combining them. Individuals above certain thresholds are classified as likely Hispanic.

10. Imputing Probability of Survival

Probability of survival of individuals are imputed in two ways. First, we can impute the probability as 0 for those with missing age of death (presuming that they are dead). Second, we

can take a more sophisticated approach using the fact that the person was at least alive at the time of discharge and using the conditional probably of survival after having survived to age a_d at the time of discharge. This probability of survival uses information of survival probabilities from age a_d to a desired age threshold, e.g. $\bar{a}$ = 70. These rates can be obtained from the corresponding cohort life tables put out by the SSA (Bell and Miller 2005) for each enrollee's birth cohort, b.

We estimate survival models where we make various assumptions about the missing data. We concentrate on survival to age 70, which is slightly below the median age at death (73). Because the number 70 is a round multiple of ten, it avoids issues of age heaping. Appendix Table 4 shows the results. We start by estimating survival models using only the sample without missing data for reference (Panel A). Panel A shows the same basic patterns we found in Table 2: those who trained longer were more likely to survive and the estimates are very stable. In the last specification, the results imply that one more year of training increased the probability of survival to age 70 by about 4.6% relative to the mean. Panel B shows the results when we impute the probability of survival using life tables and information on the age at the time of training. Here, we find that the effect of training duration (once we add all controls) is somewhat lower (2.3. instead of 3 percentage points) but still statistically significant.

In Panel C, we impute all missing as zero (we assume that all the men for whom survival is missing died before age 70). The rationale for doing this is that the DMF and other sources of death tend to be complete starting in the 1970s (Hill and Rosenwaike, 2001). If most of the missing data is missing because of death certificates are not available to researchers (rather than due to errors in matching) then all the missing deaths occurred between the CCC training and 1970, much before our CCC men turned 70 (recall most of the men were born around 1920). When we do this, we find that one more year of training is associated with about a 5% increase in survival relative to the mean.

B. Matching Individuals to 1940 Census and WWII Enlistment Records

This appendix overviews the matching approach used to match CCC participants to Census and WW2 Army enlistment records. We rely on the Expectation Maximization approach to match records. Overall, the match rates are consistent with standard literature and the matches seem consistent. There seems to be some selection in terms of who is matched.

1. Introduction to Matching Approach

The matching approach follows "Linking Individuals Across Historical Sources: a Fully Automated Approach" by Ran Abramitzky, Roy Mill, and Santiago Perez (2018).[1] Any matching approach has to balance three competing goals:

1. Minimize false negatives (Type II errors)
2. Minimize false positives (Type I errors)
3. Create a representative sample

Ideally records would be identified by a unique administrative identifier that is stable across datasets (e.g., social security number). In most historical cases, we are forced to rely on a combination of less definitive information, such as year of birth, name, place of birth, and place of residence to match records. Therefore, choosing how to match on these characteristics is a major decision. There are three major sources of variation in variables across records for a given individual. First, the respondent introduces variation. They could state the wrong age or change their name (e.g., ``Nick" instead of ``Nicholas"). This issue is especially prevalent in historical Censuses due to lower literacy and education levels. Secondly, the interviewer can make transcription errors (e.g., write the name as ``Brian" instead of ``Ryan"). Finally, additional errors are introduced during the digitization of physical Census rolls.

We choose to rely on the Expectation Maximization (EM) approach outlined in Abramitzky et al. (2018). Individuals are matched to 1940 Census and WWII enlistment records primarily using automated methods. One alternative approach would be to rely on exact matches but relying solely on exact matches would significantly lower match rates and increase Type II errors. There are significant transcription errors in these records and the EM approach allows some flexibility when dealing with errors.

The EM approach falls under the umbrella of automated methods. The advantages of automated methods include the fact that they are reproducible, rule-based, can compare all records, and are cheaper. The disadvantages are that they do not have the same contextual information that humans do (e.g., "Bill" is short for "William") and humans are better able to incorporate additional information in a flexible manner.

Bailey et al. (2018) raised substantive concerns about using automated methods as opposed to linking by hand. They find that automated linking algorithms produce high rates of incorrect matches ranging from 13 to 69 percent when assuming hand-linked sample is the ground truth.

[1] Please see this article for a more detailed description of the approach

Match rates are especially poor when automated methods are combined with phonetic name cleaning. They tested three automated methods, Ferrie (1996), iterative method of Abramitzky et al. (2012 and 2014), and the regression prediction approach of Feigenbaum (2016), though not the EM approach. These results are an issue because poor matches can significantly attenuate estimates.

Abramitzky et al. (2018) find much better results for more modern automated methods, such as the EM approach, than the approaches tested in Bailey et al. (2018). Additionally, they find that automated methods perform similarly to hand-linking methods when the same information is used. Conservative EM methods tested by Abramitzky et al. had <10% false match rate, which was lower than hand-linking methods with the same information, though hand-linking methods also made significantly more matches. Moreover, when both methods made a match then there was greater than 90% agreement.

In order to address concerns of false matches we rely on conservative matching criteria and do not conduct phonetic cleaning or significant name standardization. Finally, we validate a subset of matches against hand matches provided by Family Search (FS).

2. Overview of Matching Procedure

There are several decisions to make before beginning any estimation. The first decision is which variables to match on. The standard approach is to match on pre-determined characteristics. Typically, this means birth year, place of birth, first name and last name.

The second decision is which variables to block on. The approach will only compute distance between individuals who are exact matches on certain characteristics. Fundamentally, blocking is used to reduce computational complexity by avoiding computing distances between every potential pair of individuals. For example, it is common to block on the first letter of the first name.

The third decision is how to measure string distance. Some approaches effectively use an indicator for whether names are an exact match or they combine this approach with a phonetic cleaning algorithm, such as the NYSIIS. Phonetic cleaning is especially useful if most errors are due to translating a heard name to a written one. Continuous string distance measures can also be used and are most useful when errors are due to transcription mistakes during digitization.

Now, we present the basic concept behind the Expectation Maximization algorithm (Dempster, Laird, and Rubin 1977; Winkler 1989). For any observation, there are many match candidate pairs, *i*. For each candidate pair we observe distances γ_i. Assume each of these candidates are drawn from one of two distributions. Each candidate pair has two associated probabilities: one for true matches, $P(\gamma_i|Match_i)$, and one for false matches, $P(\gamma_i|NotMatch_i)$. Using Bayes Rule, the probability that our candidate pair *i*, with distance γ_i, is a true match is given by:

$$P(Match|\gamma_i) = \frac{P(\gamma_i|Match_i)}{P(\gamma_i|Match_i)p_{m+}\, P(\gamma_i|NotMatch_i)(1-p_m)}$$

Using these expressions, we can take the following approach to estimate match probabilities:

1. Define distribution families for each of the distance variables to get $P(\gamma_i|Match_i)$ and $P(\gamma_i|NotMatch_i)$. Assume distances for each variable are independently distributed conditional on match status
2. Guess initial parameter values $\theta_m^{(t)}, \theta_{nm}^{(t)}$ for each distribution and the probability of a true match, $p_m^{(t)}$
3. Loop over the following two steps until convergence:
   A. Calculate for each pair the probability of a match, $w_i^{(t)} = P(Match|\gamma_i)$ for a given $(\theta_m^{(t)}, \theta_{nm}^{(t)}, p_m^{(t)})$
   B. Get updated parameter estimates $(\hat{\theta}_m^{(t+1)}, \hat{\theta}_{nm}^{(t+1)}, \hat{p}_m^{(t+1)})$ by maximizing:

$$\log L(\gamma, \theta, p_m) = \sum_{i=1}^{n} w_i^{(t)} \log p_m P(\gamma_i|\theta_m) + \left(1 - w_i^{(t)}\right) \log(1 - p_m) P(\gamma_i|\theta_{nm})$$

Once we have the converged estimates then we can compute $P(\gamma_i|Match_i)$ for any candidate pair. The final major choice is choosing what qualifies as a match. There are two components to this decision:

1. The minimum threshold in order to qualify as a match
2. The maximum threshold for the second closest match

(1) means that if there are no ``good" matches then it is better not to declare any a match. (2) means that if there are at least two ``good" candidates then there is a high Type II error rate when selecting one over the other. For the primary analysis, we take a conservative approach, setting a high threshold for (1) and (2).[2]

3. Implementation

One significant issue is that New Mexico CCC records do not contain data on the birthplace of participants. When matching to the 1940 Census and WW2 records we rely on a two step procedure to create matches:

- **First stage:** Colorado and New Mexico CCC participants are matched to 1940 Census and WW2 enlistment records
  - *Blocking variables:* State of residence, first letter of first name and first letter of last name
  - *Matching variables:* Year of birth and name distances
- **Second stage:** Next, we remove matched individuals and for unmatched individuals in the Colorado CCC we conduct a second round of matching
  - *Blocking variables:* Place of birth, first letter of first name and last name
  - *Matching variables:* Year of birth and name distances

In the first stage we look only within the current state of residence (e.g., only look at residents of Colorado in the 1940 Census for CO CCC participants). In the second stage, we use the additional information on place of birth for CO CCC participants to search across the United States.

[2] The threshold for (1) is 0.8 and the minimum distance for the second best match (2) is 0.3

The primary concern with using the state of residence is that we will miss migrants. There are two reasons that this should not be a major issue in our case. First, the 1940 Census, most CCC enlistment, and most WW2 enlistment take place in a relatively short time frame. Secondly, we can check the number of migrants in the Family Search hand-links. For both CO (91.4%) and NM (96.8%) most of the CCC participants are still in the same state during the 1940 Census. For New Mexico it seems very reasonable to only look within the state. The percentage is somewhat lower for Colorado, which is why we conduct the second stage and also match on place of birth.

Next, we decide to use the Jaro-Winkler string distance (Jaro 1989, Winkler 2006). The Jaro-Winkler string distance calculates the number of transpositions required to match two strings, weighting errors in the early part of the string more heavily. The distance is measured from 0 (no matching characters) to 1 (exact match). We invert this scale so that 0 is exact match and 1 is no matching characters so our measure is increasing in distance. In our case the largest concern is transcription errors during digitization so it makes sense to use a string distance measure.

The next choice is the creation of distributions for distance variables. We follow Abramitizky et al. (2018) and specify multinomial distributions for year of birth and name distances. Year of birth distances are segmented into groupings of 0, 1, or 2 years distance.[3] Name distances are segmented based on Jaro-Winkler scores into groupings: [0,0.067], (0.067,0.120],(0.120,0.250],(0.250,1]. These groups run from closest to farthest distance.

We also add in the hand-matches from Family Search. If the Family Search matches conflict with the automated methods then we use the Family Search match. Finally, we also conduct a tie-breaking procedure using additional information in cases where the best match clears the minimum threshold but the second best match is too close. If the first best match passes the tiebreak criteria and second best match fails then we count it as a match. Middle initial is used as a tiebreaker in both stages, while place of birth is used as a tiebreaker in the first stage for Colorado. For example, if the CCC record has middle initial "F", the first best match also has the middle initial "F" but the second best match has the middle initial "M" then it is counted as a match.

4. Census Matching Results

Matching Appendix Table 7-1: Match rates between CCC records and 1940 Census

[3] Matches with larger distances are not considered

Census Match Rates by Type	CO	NM	Overall
EM and FS	0.08	0.06	0.07
Only EM	0.34	0.22	0.30
Only FS	0.05	0.09	0.07
No match	0.53	0.63	0.56
Observations	18644	9699	28343

Note: Values represent match rates as percentages of column totals. Match rates are for CCC participants to 1940 Census. EM stands for Expectation Maximization approach, FS stands for hand matches by FamilySearch team

False Negatives (Type II error): Matching Appendix Table 7-1 shows that 44% of CCC participants have been matched to 1940 Census records. 30% of participants have been matched through EM only, 7% through FS only, and 7% through both methodologies. This match rate for the EM approach is in line with the literature. Additionally, there is an upper bound on potential matches. In order to find this upper bound for matches to the 1940 Census, Abramitzky et al. (2018) linked a copy of the 1940 Census digitized by Family Search and one digitized by Ancestry.com. Even in this case they can only link up to 67% of the Census due to individuals with similar attributes and ``brutally bad transcriptions'' due to difficulties reading cursive.

Matching Appendix Table 7-2: Match consistency between EM and FS for CCC-1940 Census matches

Census Match Consistency	CO	NM	Overall
% of participants matched by both EM and FS	0.08	0.06	0.07
% of overlap matched to same individual	0.95	0.91	0.94
Observations	18644	9699	28343

Note: Values represent percentages of column totals. Match rates are for CCC participants to 1940 Census. EM stands for Expectation Maximization approach, FS stands for hand matches by Family-Search team. Consistent FS and EM match measures whether EM and FS approaches matched CCC participant to the same Census individual in cases when both approaches make a match

False Positives (Type I error): While we do not have an absolute ``ground truth'' sample, one way of examining Type I errors is to see if the EM and FS approaches match the same individual when they overlap. As seen in Matching Appendix Table 7-2, there is a high degree of consistency when both methods made a match - 94% of the time they matched the same CCC participant to the same Census record. We can go a step further and examine the discrepancies to understand if there is a reason to prefer the EM approach or FS hand matches. We use additional information (e.g., county of residence) and classify the discrepancies. In about 1/3 of cases the EM match is preferred, in 1/3 of cases the FS match is preferred, and the remaining cases are indeterminate. Therefore, there does not seem to be a clear reason to prefer either method.

Representativeness: Finally, we check which individuals are matched by regressing an indicator of whether matched on CCC participant characteristics at the time of their first enrollment. If matches are at random then there should be no clear pattern.

Matching Appendix Table 7-3: Predictors of CCC-1940 Census matches by type of match for CO

	EM match	FS match	EM and FS	FS or EM match
Age at enrollment	0.00	-0.00**	-0.00	-0.00
	(1.00)	(0.01)	(0.19)	(0.29)
Age of death	0.00	-0.00*	-0.00**	0.00
	(0.15)	(0.04)	(0.01)	(0.13)
Enroll year	-0.00	-0.00	-0.00	-0.00
	(0.80)	(0.27)	(0.46)	(0.54)
Dist. to camp (mi)	-0.00***	-0.00***	-0.00***	-0.00***
	(0.00)	(0.00)	(0.00)	(0.00)
Born in CO	-0.01	0.00	-0.01	-0.00
	(0.14)	(1.00)	(0.09)	(0.60)
Height (in)	0.02*	0.01	0.01**	0.01*
	(0.01)	(0.08)	(0.01)	(0.03)
Weight (lb)	-0.00	-0.00	-0.00	-0.00
	(0.49)	(0.41)	(0.10)	(0.74)
BMI	0.01	0.01	0.01*	0.00
	(0.35)	(0.30)	(0.04)	(0.61)
Missing parent	-0.06***	-0.07***	-0.04***	-0.08***
	(0.00)	(0.00)	(0.00)	(0.00)
Farm	0.01	0.05***	0.03***	0.03
	(0.45)	(0.00)	(0.00)	(0.07)
Urban	-0.00	-0.02	-0.01	-0.01
	(0.76)	(0.18)	(0.15)	(0.65)
Years educ	0.01***	0.00*	0.00***	0.01***
	(0.00)	(0.02)	(0.00)	(0.00)
Unemployed	-0.02	-0.01	-0.02	-0.02
	(0.29)	(0.25)	(0.08)	(0.38)
Constant	1.17	5.37	2.47	4.07
	(0.87)	(0.29)	(0.54)	(0.58)
Observations	18644	18644	18644	18644
R^2	0.044	0.018	0.018	0.049

* $p < 0.05$, ** $p < 0.01$, *** $p < 0.001$

Note: p-values in parentheses. Match rates are for CO CCC participants to 1940 Census. EM stands for Expectation Maximization approach, FS stands for hand matches by FamilySearch team

Matching Appendix Table 7-3 shows the results for Colorado CCC participants broken out by match type. In general, CCC participants who were matched seem slightly better off. For example, matched individuals have higher education levels, less likely to be missing parents, and are taller on average. These differences do not seem to be large in absolute magnitude though, so it seems as though the matches are reasonably well representative.

Matching Appendix Table 7-4: Predictors of CCC-1940 Census matches by type of match for NM

	EM match	FS match	EM and FS	FS or EM match
Age at enrollment	-0.00	-0.00**	-0.00*	-0.00**
	(0.07)	(0.01)	(0.05)	(0.01)
Age of death	0.00	-0.00	-0.00	0.00
	(0.74)	(0.15)	(0.05)	(0.86)
Enroll year	-0.00	0.00	-0.00	-0.00
	(0.42)	(0.92)	(0.66)	(0.65)
Dist. to camp (mi)	-0.00	0.00	0.00	-0.00
	(0.20)	(0.35)	(0.48)	(0.40)
Constant	4.98	-0.24	1.47	3.27
	(0.39)	(0.96)	(0.63)	(0.60)
Observations	9699	9699	9699	9699
R^2	0.019	0.003	0.003	0.016

* $p < 0.05$, ** $p < 0.01$, *** $p < 0.001$

Note: *p*-values in parentheses. Match rates are for NM CCC participants to 1940 Census. EM stands for Expectation Maximization approach, FS stands for hand matches by FamilySearch team

Matching Appendix Table 7-4 shows the results for New Mexico matches. For New Mexico, we have significantly fewer indicators of participant characteristics; however, there again seems to not be large differences in terms of the type of individual matched.

5. WW2 Matching Results

Matching Appendix Table 7-5: Match rates between CCC records and WWII Enlistment records

WW2 Match Rates	CO	NM	Overall
EM match	0.31	0.24	0.29
EM match (Adj)	0.78	0.59	0.72
Observations	18644	9699	28343

Note: Values represent match rates percentages of column totals. Adjusted values are scaled by state-age cohort enlistment percentages

<u>False Negatives (Type II error):</u> Matching Appendix Table 7-5 shows that 29% of CCC participants are matched to WW2 army enlistment records. There are two primary reasons that this match rate is lower than the 1940 Census. First, there is no supplementary source of matches to augment the EM approach with (FS matches). Secondly, the Census has universal coverage while only a subset of men will be in the WW2 army enlistment records. We can compute an adjusted match rate by estimating the percentage of men in each state-year of birth cell that are in the records.[4] This procedure assumes CCC participants are no more likely to enlist than other of the same age in the same state. Based on these calculations we would expect 40% of the Colorado CCC participants and 41% of the New Mexico CCC participants to have be in the WW2 army enlistment records. The adjusted match rates (match percentage of those we expect to find) and is 78% for Colorado and 59% for New Mexico. Note that these adjusted match rates seem high but cannot account for whether CCC individuals were more likely to serve in the

[4] Using state-year of birth-years of education cells does not substantively alter the results

Army. For example, CCC camps typically involved significant Army administration which could increase the likelihood to serve due to familiarity with the military.

False Positives (Type I error): Without another source of matches for the WWII data it is difficult to conduct any sort of consistency analysis. Therefore, we rely on the findings of high consistency in the CCC to 1940 Census matches in order to support the EM approach in this case.

Representativeness: We repeat the regression of match status on characteristics, but the interpretation is slightly complicated in this case. There are two forms of selection: first, selection into who is drafted (and meets minimum standards) or enrolled in the Army, and secondly there is selection through who is matched.

Matching Appendix Table 7-6: Predictors of CCC-WWII enlistment matches by type of match for CO

	EM match
Age at enrollment	-0.01***
	(0.00)
Age of death	0.00
	(0.94)
Enroll year	0.01***
	(0.00)
Dist. to camp (mi)	-0.00*
	(0.01)
Born in CO	0.02**
	(0.00)
Height (in)	-0.01
	(0.15)
Weight (lb)	0.00
	(0.14)
BMI	-0.01
	(0.12)
Missing parent	-0.01
	(0.63)
Farm	0.02
	(0.22)
Urban	-0.02
	(0.19)
Years educ	0.01***
	(0.00)
Unemployed	0.01
	(0.68)
Constant	-26.86***
	(0.00)
Observations	18644
R^2	0.041

* $p < 0.05$, ** $p < 0.01$, *** $p < 0.001$

Note: p-values in parentheses. Match rates are for CO CCC participants to WW2 enlistment records. EM stands for Expectation Maximization approach

Matching Appendix Table 7-6 shows the results for Colorado CCC participants. Matched individuals are again better educated, but most indicators are not statistically significant. Matching Appendix Table 7-7 shows the results for New Mexico CCC participants.

Matching Appendix Table 7-7: Predictors of CCC-WWII enlistment matches by type of match for CO

	EM match
Age at enrollment	0.00**
	(0.00)
Age of death	-0.00
	(0.79)
Enroll year	-0.02***
	(0.00)
Dist. to camp (mi)	-0.00
	(0.06)
Constant	40.74***
	(0.00)
Observations	9699
R^2	0.035

* $p < 0.05$, ** $p < 0.01$, *** $p < 0.001$
Note: p-values in parentheses. Match rates are for NM CCC participants to WW2 enlistment records. EM stands for Expectation Maximization approach

C. Control Function Approach

In this section we explore the control function approach in detail, beginning with the original approach in Athey, Chetty, and Imbens (2020) then discussing our extension.

1. Athey Chetty Imbens (2020)

In Athey Chetty Imbens (2020) (henceforth ACI) the set-up is an experimental sample with only the secondary (short-term) outcome and observational sample with both the secondary and primary (long-term) outcomes. The question they address is how the experimental sample can be used to obtain the treatment effect on the long-term outcome that is observed only in the observational sample.

ACI has four assumptions that allows us to recover τ_O^P, reproduced here:

Assumption 1. (External Validity of the Observational Study) *The observational sample is a random sample of the population of interest.*

This assumption exists to set the baseline of the analysis to the observational sample, and is essentially definitional.

Assumption 2. (Internal Validity of the Experimental Sample) *For* $w = 0, 1$,

$$W_i \perp\!\!\!\perp \left(Y_i^P(w), Y_i^S(w)\right) | X_i, G_i = E \tag{A1}$$

This assumption allows us to estimate treatment effects in the experimental sample without bias.

Assumption 3. (Conditional External Validity) *The experimental study has conditional external validity if*

$$G_i \perp\!\!\!\perp \left(Y_i^P(0), Y_i^P(1), Y_i^S(0), Y_i^S(1)\right) | X_i \tag{A2}$$

Assumption 3 implies that the conditional average treatment effect in both samples is the

same as $E[Y_i^S(1) - Y_i^S(0)|X_i, G_i = O] = E[Y_i^S(1) - Y_i^S(0)|X_i, G_i = E]$. Assumption 3 also implies that $\tau_O^S = \tau_E^S$ and $\sigma_O^S = \sigma_E^S$.

Finally, the last assumption relates the secondary (short-term) outcomes to primary (long-term) outcomes:

Assumption 5. (Latent Unconfoundedness) *For* $w = 0,1$,

$$W_i \perp\!\!\!\perp Y_i^P(w)|X_i, Y_i^S(w), G_i = O \tag{A3}$$

This allows ACI to identify τ_O^P by inferring the bias in the observational sample from the estimated treatment effects on the secondary outcome in the two samples, and transfer that to the primary (long term) outcome.

2. ACI Linear Setting and Our Approach

In ACI linear setting, the short-term outcomes have the following formulation,

$$\begin{aligned} Y_i(0) &= X_i^T\gamma + \alpha_i \\ Y_i(1) &= Y_i(0) + \tau_g \\ Y_i &= \tau_g W_i + X_i^T\gamma + \alpha_i \end{aligned}$$

Furthermore, they assume a stronger version of A5,

Assumption 5' Linear Latent Unconfoundedness

$$\begin{aligned} \alpha_i^P &= \delta\alpha_i^S + \varepsilon_i^P \\ W_i &\perp\!\!\!\perp \varepsilon_i^P|X_i, \alpha_i^S, G_i = O \end{aligned}$$

In our approach, we differ with ACI's linear setting in two ways. First, we use continuous treatment, which makes Assumption 5 into a stronger one. Second, instead of the ACI Assumption 3 that the experimental sample is externally valid for the observational sample,

we consider two different approaches: first, we assume that the short-term treatment effect are the same between the two samples, and second, we assume that the short-term bias is the same and utilize the instrument in the JC sample. In the most favorable case both lead to the same results because the observational study has internal validity from the outset.

Our first approach takes the assumption that the short-term treatment effect between CCC and JC samples are the same, or in our notation, $\tau_E^S = \tau_O^S$. Using the IV approach in the JC sample, we can obtain an unbiased estimate of τ_E^S, which in turn gives us an unbiased estimate of τ_O^S. Finally, we can construct the control function as in ACI

$$\hat{\alpha}_i^S = Y_i^S - W_i \hat{\tau}_O^S - X_i^T \hat{\gamma}^S \tag{A4}$$

and include the control function in the long-term regression of the observational sample.

Our second approach assumes that the (linear) selection bias is the same between CCC and JC. In this approach, we exploit the fact that we have an instrument for duration in the JC sample. Therefore, we can think of the difference between the IV estimate and the OLS estimate gives us an estimate of the bias in the JC sample,

$$\hat{\sigma} = \hat{\tau}_{E,OLS}^S - \hat{\tau}_{E,2SLS}^S \tag{A5}$$

Then, adjusting the OLS estimate of the short-term treatment effect from the CCC sample $\hat{\tau}_O^S - \hat{\mu}$ gives us an unbiased estimate of the short-term treatment effect of the CCC sample. Finally, we construct the control function as before and include in the long-term regression of the observational sample.

We present a complete step-by-step description here. To make notations easier to interpret in the description of the approaches, we replace the experimental sample subscript E by JC for Jobs Corps and observational sample subscript O by CCC for CCC. Additionally, we replace secondary outcome sample superscript S by ST for short-term and the primary outcome sample superscript P by LT for long-term.

Approach 1: Assuming treatment effect is the same

1. Using the (experimental) JC data we estimate the short-term treatment effects for outcomes available in both the CCC and JC data. These include schooling, employment, earnings and geographic mobility. Using the JC sample, we instrument for the duration W using the random assignment T. This procedure gives us an unbiased estimate of the short-run treatment effect in the JC, as well as in the CCC (by assumption).

2. Estimate the residual in the CCC data using the estimated ST treatment effect from the JC RCT ($\hat{\tau}_{JC}^{ST}$)

$$\hat{\alpha}_{iCCC}^{ST} = Y_{iCCC}^{ST} - \hat{\gamma} X_{iCCC} - \hat{\tau}_{JC}^{ST} W_{iCCC} \tag{A6}$$

3. Include the ST residuals calculated in step 2 ($\hat{\alpha}_{iCCC}^{ST}$) as controls in the LT CCC regressions:

$$Y_{iCCC}^{LT} = X_{iCCC} \gamma_{CCC}^{LT} + \tau_{CCC}^{LT} W_{iCCC} + \delta \alpha_{iCCC}^{ST} + \varepsilon_{iCCC}^{ST} \tag{A7}$$

Approach 2: Assuming selection bias is the same

1. Estimate ST treatment effect from RCT using both OLS and 2SLS. We use random assignment to treatment as instrument for duration to construct the 2SLS estimates. We construct the OLS estimates using the treated arm of the experiment only.

2. Estimate the selection or omitted variable bias term ($\hat{\mu}$) by subtracting JC's 2SLS estimate from JC's OLS estimate of ST treatment

$$\hat{\mu} = \hat{\tau}_{JC,OLS}^{ST} - \hat{\tau}_{JC,2SLS}^{ST} \tag{A8}$$

3. Estimate ST treatment effect in the CCC sample and adjust it by the estimated selection or OVB term ($\hat{\mu}$)

$$\hat{\tau}_{CCC}^{ST} = \hat{\tau}_{CCC,OLS}^{ST} - \hat{\mu} \tag{A9}$$

4. Estimate the residual of the ST treatment effect using our adjusted estimate of the short-term treatment effect ($\hat{\tau}^{ST}_{CCC}$)

$$\hat{\alpha}^{ST}_{iCCC} = Y^{ST}_{iCCC} - \hat{\gamma} X_{iCCC} - \hat{\tau}^{ST}_{CCC} W_{iCCC} \tag{A10}$$

5. Include estimated residual in LT treatment effect regression in order to generate an unbiased estimate of the long-term impact of the CCC on outcomes.

$$Y^{LT}_{iCCC} = X_{iCCC} \gamma^{LT}_{CCC} + \tau^{LT}_{CCC} W_{iCCC} + \delta \alpha^{ST}_{iCCC} + \varepsilon^{ST}_{iCCC} \tag{A11}$$

3. Quantifying the Effect of Violations of Assumptions

In each of our two approaches, we make an assumption that allows us to recover τ^S_O without bias in large samples. In the first approach, we assume that $\tau^S_{CCC} = \tau^S_{JC}$, and in the second approach, we assume that $\sigma^S_{CCC} = \sigma^S_{JC}$.

In practice it is plausible that neither assumption holds exactly. So, let us suppose that both these assumptions are violated, and we estimate short-term TE in the observational sample with bias. let the bias be denoted by so $\phi = \hat{\tau}^S_O - \tau^S_O$. In our first approach, ϕ is the difference between JC and CCC short-term treatment effects, In our second approach, ϕ is the difference in the short-term bias between JC and CCC. We can characterize the biases for the two approaches. In general if the short term effects are similar, even if not identical, the first approach is preferable, whereas if the biases are similar, but not identical, the second approach is preferable.

Then,

$$\begin{aligned}
\hat{\alpha}^S_i &= Y^S_i - W_i \hat{\tau}^S_O - X^T_i \hat{\gamma}^S \\
&= \alpha^S_i - W_i * \phi \\
\hat{\alpha}^P_i &= \alpha^P_i - (\delta * \phi) W_i
\end{aligned}$$

and so regressing primary outcomes on duration, X, and control function will be mis-specified

$$Y_i^P = (\tau_P - \delta * \phi)W_i + X_i^T\gamma + \delta\alpha_i^S + \varepsilon_i^P \tag{A12}$$

which yields a final bias of $-\delta * \phi$.

In our first approach, where we assume that short-term treatment effects are identical, ϕ term is the difference between JC and CCC short-term treatment effects, so $bias = -\delta * (\tau_{CCC}^S - \tau_{JC}^S)$. Expressing this in terms of percentage difference in short-term treatment effects,

$$bias_1 = -\delta * \tau_{JC}^S * \%\Delta\tau^S \tag{A13}$$

where $\%\Delta\tau^S = \frac{\tau_{CCC}^S - \tau_{JC}^S}{\tau_{JC}^S}$.

In our second approach, ϕ is the difference in the short-term bias between JC and CCC, so the bias is $bias = -\delta * (\Delta(\text{short-term bias}))$ or

$$bias_2 = -\delta * \left(\Delta\frac{1}{sd(W_i)^S} * \beta_2^S corr(W_i, U_i)_{JC}^S + \frac{1}{sd(W_i)_{CCC}^S} * \Delta[\beta_2^S corr(W_i, U_i)^S]\right) \tag{A14}$$

where $\beta_2^S corr(W_i, U_i)_{JC}^S$ is a component of the omitted variable bias in short-run regression of JC and $\Delta[\beta_2^S corr(W_i, U_i)^S]$ is the difference in the components between CCC and JC.[1] Everything except $\Delta[\beta_2^S corr(W_i, U_i)^S]$ is observed.

So after the estimate of the long-term treatment effect is first adjusted by, $-\delta * \Delta\frac{1}{sd(W_i)^S} * \beta_2^S corr(W_i, U_i)_{JC}^S$, the remaining bias for the long term effect, expressed in terms of percentage difference of the short-term bias term is,

$$-\delta * \frac{\beta_2^S corr(W_i, U_i)_{JC}^S}{sd(W_i)_{CCC}^S} * \%\Delta[\beta_2^S corr(W_i, U_i)^S] \tag{A15}$$

[1]In a regression setting $Y_i = \beta_0 + \beta_1 W_i + \beta_2 U_i + \eta_i$, the omitted variable bias when U_i is omitted can be expressed as $\beta_2 corr(W_i, U_i)\frac{sd(U_i)}{sd(W_i)}$

4. Heterogeneous Treatment Effects

We cannot allow for unrestricted heterogeneity at the individual level. For example, if we allow for an i subscript on τ_S, treatment effect heterogeneity will lead the residual from a projection of Y_{iS}^{ST} on W_{iS} to exhibit a variance that depends positively on $(W_{iS} - \bar{W}_S)^2$. In this case the estimator would no longer be consistent for the average effect. Endogenous heteroskedasticity of this nature is a common problem in many empirical applications. If the heterogeneity is limited one would expect the biases to be modest

We can however make some progress on this issue. For heterogeneity in long-term outcomes, we can interact the residuals with the treatment variable. For heterogeneity in the short-term outcomes, one way to address this problem is to make use of the fact that we have more than one short-term outcome. For every short-term outcome, we can calculate a residual, that can be used to weaken the assumption that the treatment effect is additive in the unobserved selection component. We can therefore use the multiple short-term outcomes to allow for some treatment effect heterogeneity.

Suppose we have the following for the long-term outcome:

$$Y_{iS}^{LT} = \tau_{iS}^{LT} W_i + \alpha_{iS}^{LT}$$

As before, we are concerned about the endogeneity of W_i. We use residuals extracted from short-term outcome regression, $Y_{iS}^{ST} = \tau_S^{ST} W_{iS} + \alpha_{iS}^{ST}$ to address this, by including them in the long-term regression. However, there is nothing stopping us from using those residuals in a more complex way. A natural way to do so would be by interacting them with the treatment:

$$Y_{iS}^{LT} = \tau^{LT} W_{iS} + \gamma^{LT} \hat{\alpha}_{iS}^{ST} W_i + \hat{\alpha}_{iS}^{ST} + \varepsilon_i$$

This works, whether there is a single short-term outcome or multiple ones.

One might instead be concerned with treatment effect heterogeneity in the control function approach for the short-term outcomes. Now the proposed method does not work the

exact way we used it before, because there are essentially two residuals, as we can write it as

$$Y_{iS}^{ST} = \bar{\tau}_S W_{iS} + \alpha_{iS}^{ST} + \eta_{iS}^{ST} W_{iS} \tag{A16}$$

where $\eta_{iS}^{ST} = \tau_{iS}^{ST} - \bar{\tau} S^{ST}$

What we need is a second short-term outcome so that $Y_{iS}^{ST,2} = g(W_{iS}, \alpha_{iS}^{ST}, \eta_{iS}^{ST})$ so that based on the short-term data we can recover both residuals α_{iS}^{ST} and η_{iS}^{ST}. Once we have both we can control for both in the long-run regression. So the two residuals would capture differences in the level of the outcome (α) and the slope (η). Assuming it is the same two residuals affecting both short term outcomes is of course a strong assumption, but it does address the concern that we cannot deal with heterogeneity at all.

D. Calculation of Marginal Value of Public Funds

We first calculate the cost of the program. The cost measure of MVPF incorporates both the direct cost to the government and various mitigation of cost. In particular, the CCC cost measure includes the following:

1. Upfront cost of the program
2. Increased social security payout from both the increase in pension amount and increase in longevity of enrollees
3. Cost mitigation from increased tax revenue from increased earnings of the enrollees
4. Cost mitigation from decrease in social security disability (SSDI) payout from decrease in claiming rate
5. Cost mitigation from decrease in social security payout from increase in retirement age
6. Cost mitigation from decrease in SSDI payout from increase in claiming age
7. Goods produced during the program, namely conservation work

We get information on (1) from Levine 2010, who estimated the annual cost per enrollee to be $1,004. Assuming the figure is in 1939 dollars, using Consumer Price Index All Urban Consumers (CPI-U) January-to-January growth, that amounts to $14,384.81 in 2017 dollars for our average enrollee who served around 0.8 years (9.6 months).

For (2), we use the mortality profile from our regression results illustrated in Figure 5. We assume that enrollees survive to age 45 with probability 1. For each age $x > 45$, we take the average survival rate to age x of our regression sample to be the baseline survival rate, and the estimate of the coefficient on duration to be the increase in survival rate for an enrollee that served one year. Multiplying the estimate by the average duration gives us the increase in the rate of survival for our average enrollee to age x, for each age x from 46-90. We assume after age 90, the survival rate declines to 0 evenly until age 95.

The average person in our sample receives the average PIA amount of $437.70 per month, assumed to be in 1982 dollars, as 1982 is the year on which our average enrollee turns 62 when SSA starts calculating PIA using AIME. Converting that to an annual benefit amount in 2017 dollars gives us an annual benefit of $13,525.85. We assume that 65 is the claiming age for social security benefit. Multiplying i) the PIA with ii) the probability of survival to age x for each $x \geq 65$, iii) by the discount factor, and finally iv) summing the yearly amounts gives us the present value of the baseline social security benefit.

The average enrollee receives an extra $14.11 of PIA (Appendix Table 10 Panel A, Column 6 multiplied by average duration), which is an annual increase of $436.05 in 2017 dollars. Taking into account the increased survival rates to age x, multiplying the baseline benefit by the total increased survival rate and by the discount rate gives the increase in the PV of benefits from increased rate of survival. Multiplying the total increased survival rate by the discount rate and the additional PIA amount gives the increase in the PV of benefits from increased PIA amount. Summing these two and subtracting it from the baseline PV of social security benefit gives us the final cost increase from increase in social security benefit over the lifetime. The final measure amounts to $2,514.17.

Calculating (3) is similar to the above, but instead of multiplying the PIA amount for ages above 65, we multiply AIME for ages 30-65. The average enrollee in our sample has an AIME of $963.62, and an increase of $44.10 (Table 2 Panel B Column 6 multiplied by average duration), both assumed to be in 1982 dollars. We impose an additional assumption that the earnings increase does not kick in until age 30—this is to incorporate our null result of service

duration on short-term labor market outcomes as well as uniformly applying the earnings increase over the last 35 years of earnings, to mimic SSA's rule of using 35 years of highest earnings. This gives us the total PV of earnings and PV of earnings increase. We calculate the tax portion of this by assuming a tax rate of 33.6%, which is the CBO estimated average tax rate for FPL 100-149% provided in Appendix G of Hendren and Spruce-Keyser (2019). The final measure comes out to be $6,965.46 in 2017 dollars.

We calculate (4) by first calculating the baseline cost of SSDI. We assume that the average claiming age is 50 and the average SSDI amount is $1,171.80 in 2017 dollars, which is the average benefit in current payment status at the end of June 2017 from SSA's Selected Data From Social Security's Disability Program (https://www.ssa.gov/oact/STATS/dib-g3.html). Like how we calculated (2), we multiply i) this amount, ii) the average claiming rate of our sample (0.21), iii) the probability of survival at each age, iv) the discount factor, then v) sum the amounts over all years. This gives us the baseline value of SSDI claiming. We compare this with the change in SSDI amounts by taking into account the decrease of SSDI claiming probability for our average enrollee of about -0.017 (Table 2 Panel D Column 6 multiplied by average duration) as well as the increase in the probability of survival. Here we assume that the decrease in SSDI claiming probability applies uniformly across all ages. This gives us the final value of $910.61.

For (5) and (6), we use our average enrollee's increase in claiming age by 0.33 (Table 2 Panel C Column 6 multiplied by average duration). We assume that this age increase applies to retirement with probability (1 - 0.21) and to disability with probability 0.21, which is the average rate of disability claiming in our sample. For retirement, we multiply 0.33 by (1 - 0.21) and the amount the average enrollee would receive at age 65 calculated in (2), giving us $732.51. For disability, we multiply 0.33 by 0.21 and the amount average enrollee would receive at age 51 calculated in (4), which gives us $72.61.

We abstract from (7), as we have no good estimate of the total value of conservation work provided by the program. Thus, our estimate could be thought of as an upper bound of the cost.

Now, on to the WTP (or value) of the program. CCC provided the following short- and long-term benefits to enrollees:

1. Willingness to pay (WTP) for increase in longevity
2. Increase in earnings
3. Monthly real wage of $66.25 while enrolled, which includes the benefits enrollees received during the program (BLS 1941)
4. Decrease in benefit from loss of SSDI income as the enrollee claims at lower rate

Calculating (1) is again similar to the above cost calculation on increased social security payment. Instead of multiplying the PIA amount, we multiply the statistical value of life, assumed to be $150,000 in 2017 dollars (based on a reasonable midpoint from estimates surveyed in Keller et al. 2021), for ages 45 to 95. We obtain an estimate of $25,456.40. For (2), since we already obtained the PV of earnings increase and the subsequent tax increase in calculating the cost, it is simply the after-tax portion of the PV of earnings calculated there. Therefore, we have $13,642.41 for the post-tax earnings benefit. (3) is straightforward, where we take the average amount enrollees received ($66.25 multiplied by average duration), which is $11,390.36 in 2017 dollars. For (4), this value is identical to what is calculated in (4) in the cost side, $910.61.

The final measures of cost and benefit are \$8,217.78 and \$49,578.56, respectively. Finally, MVPF is equal to the ratio of WTP to Cost, which is estimated to be 6.03. Without the WTP for increase in longevity, the MVPF comes out to be 2.52.

E. CCC Regulations

The rules and regulations regarding the operation of CCC camps as well as allotment of funds to CCC employees changed from the program's inception in 1933 to its closure in 1942. Below is a compilation of CCC regulations that are pertinent to our research.

1. Each employee of the CCC was given a serial number which was composed in the following:
   a. Serial numbers started with the letters "CC" to denote the Civilian Conservation Corps as opposed to other emergency relief programs. The letters "CC" were followed by the number of the area corps number. In the case of Colorado and New Mexico, the area number was 8. See the map below (source: National Parks Service. https://www.nps.gov/parkhistory/online_books/ccc/ccc/chap2.htm).

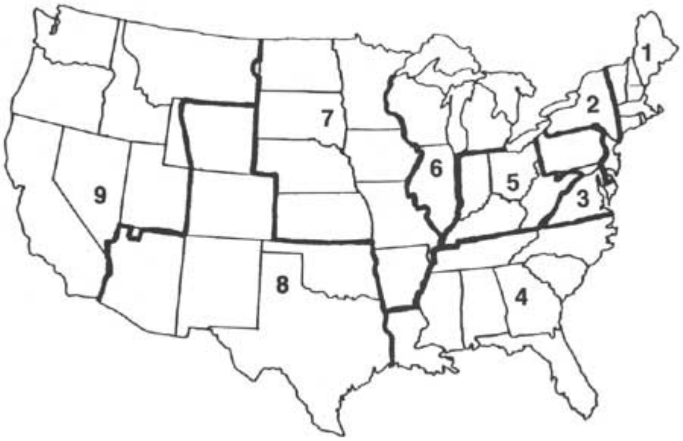

   b. Serial numbers then contain information on the company number. "In order that the numerical designation of the company may indicate its origin by corps area, blocks of numbers are assigned in accordance with the following system: 100-199 to First Corps Area, 201-299 to Second Corps Area, 901-999 to Ninth Corps Area.. When this series becomes exhausted, 1,000 will be added to each block of numbers; e.g. 1101-1199 to First Corps Area, 1201-1299 to Second Corps Area, and so on" (quote found here: https://babel.hathitrust.org/cgi/pt?id=mdp.39015020215433;view=1up;seq=17).
2. Allocation of funds received by CCC employees:
   a. Our data show that there was variation in the amounts received by CCC employees. This is consistent with the regulations found here: https://babel.hathitrust.org/cgi/pt?id=mdp.39015020215433;view=1up;seq=25 In particular, enrollees without special status (such as leaders or assistant leaders) were paid $30 per month. Of the $30 received, enrollees were required to pay at least $22 to their families.
3. Enrollment over time:
   a. CCC enrollment: Our data mostly contain information for those who enrolled after 1937. The most likely reason for this is that the CCC changed from being a program that was part of the Emergency Conservation Work program to its own entity known as the Civilian Conservation Corps in 1937. See quote here: "There are hereby transferred to the Corps all enrolled personnel, records, papers, property, funds, and obligations of the Emergency Conservation Work established

under the Act of March 31, 1933 (48 Stat. 22), as amended; and the Corps shall take over the institution of the camp exchange heretofore established and maintained, under supervision of the War Department, in connection with and aiding in administration of Civilian Conservation Corps work camps conducted under the authority of said Act as amended: Provided, That such camp exchange shall not sell to persons not connected with the operation of the Civilian Conservation Corps" (source here: https://www.nps.gov/parkhistory/online_books/ccc/cccaa.htm)

F. Special Acknowledgements

A. Dirk Van Hart provided us with dates for which New Mexico camps were open for enrollment.

B. Robert W. Audretsch provided us with dates for which Colorado camps were open for enrollment.

G. Appendix Figures

Figure A.I

History of CCC Program[5]

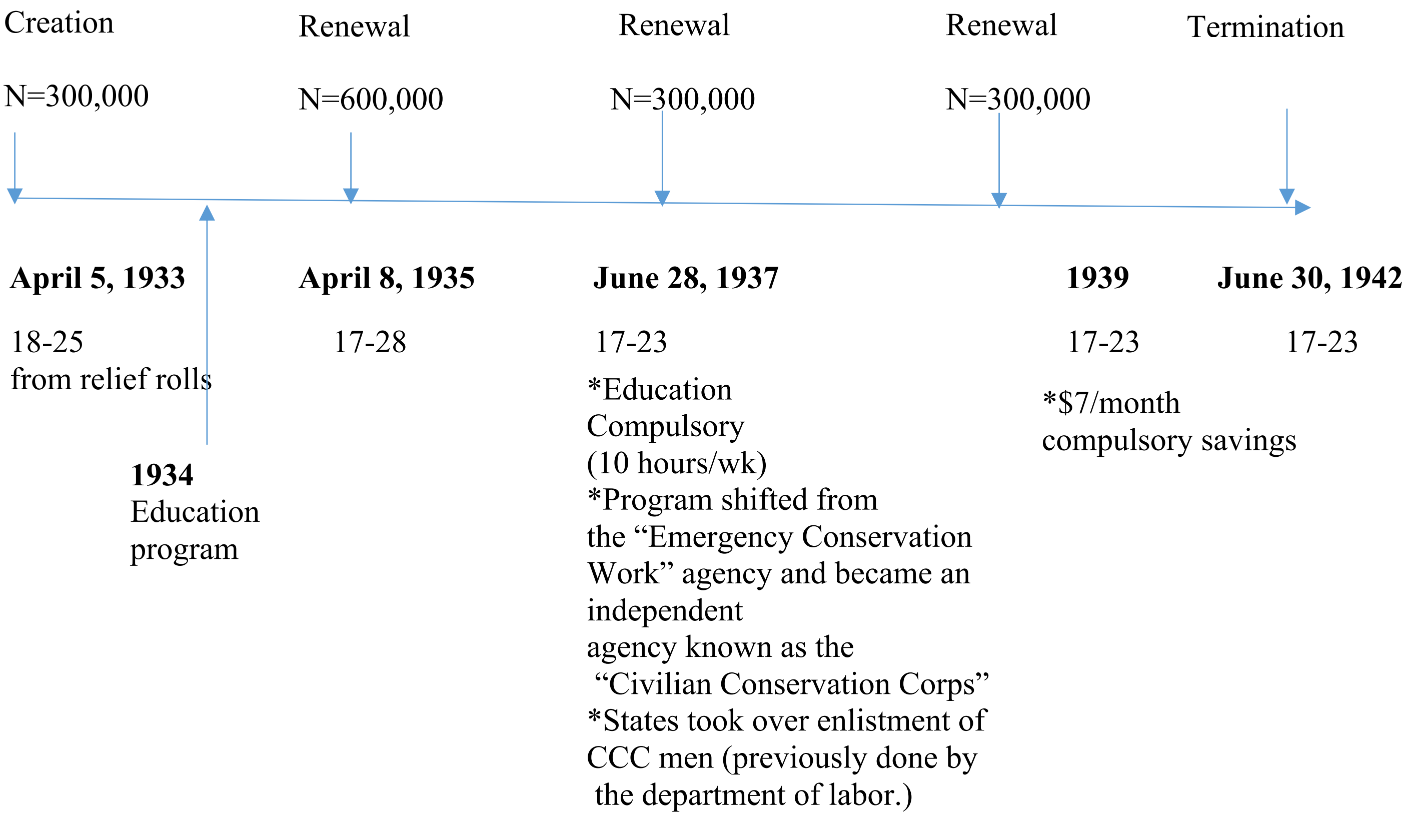

[5] Information on the history of the CCC program used in Appendix Figure 1 come from the following sources: https://babel.hathitrust.org/cgi/pt?id=mdp.39015004052794;view=1up;seq=13 On June 28, 1937, the CCC was once again renewed with funding for three additional years according to Public Law No. 163 (effective on July 1, 1937) see here: https://www.nps.gov/parkhistory/online_books/ccc/cccaa.htm

Figure A.II
Colorado and New Mexico Data Completeness
Panel A: Archival data coverage in Colorado

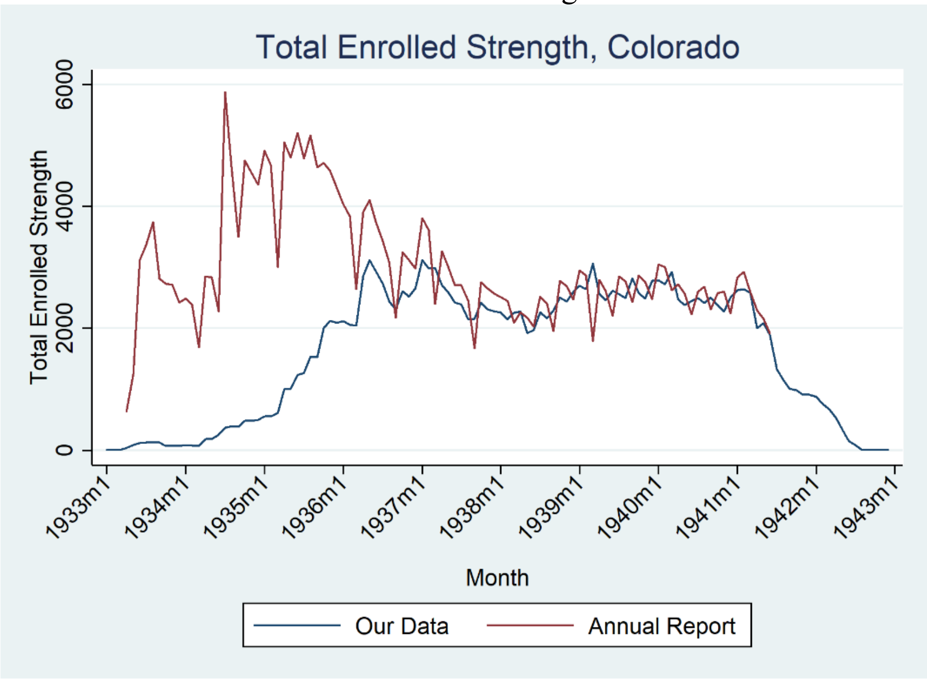

Panel B: Archival data coverage in New Mexico

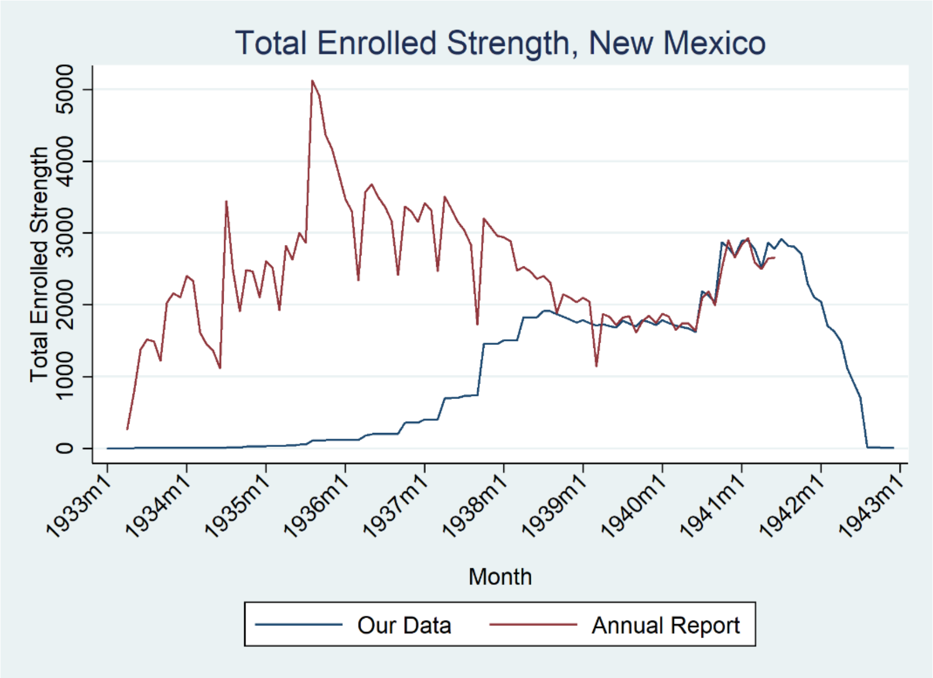

Note: Total enrolled strength is the number of enrollees at each month. Data from the Annual Report come from the following sources:

- Summary Report of the Director of Emergency Conservation Work on the Operations of Emergency Conservation Work: For the period extending from April 1933 to June 30, 1935, Appendix E
- Annual Report of the Director of Emergency Conservation Work: Fiscal Year Ending June 30, 1936, Appendix E
- Annual Report of the Director of Emergency Conservation Work: Fiscal Year Ending June 30 1937, Appendix D
- Annual Report of the Director of the Civilian Conservation Corps: Fiscal Year Ended June 30 1938, Appendix E
- Annual Report of the Director of the Civilian Conservation Corps: Fiscal Year Ended June 30 1939, Appendix I
- Annual Report of the Director of the Civilian Conservation Corps: Fiscal Year Ended June 30 1940, Appendix E
- Annual Report of the Director of the Civilian Conservation Corps: Fiscal Year Ended June 30 1941, Appendix E

Figure A.III

CCC Enrollees in CO and NM Are More Disadvantaged Than Enrollees Nationwide

Panel A: Age at Enrollment, NM

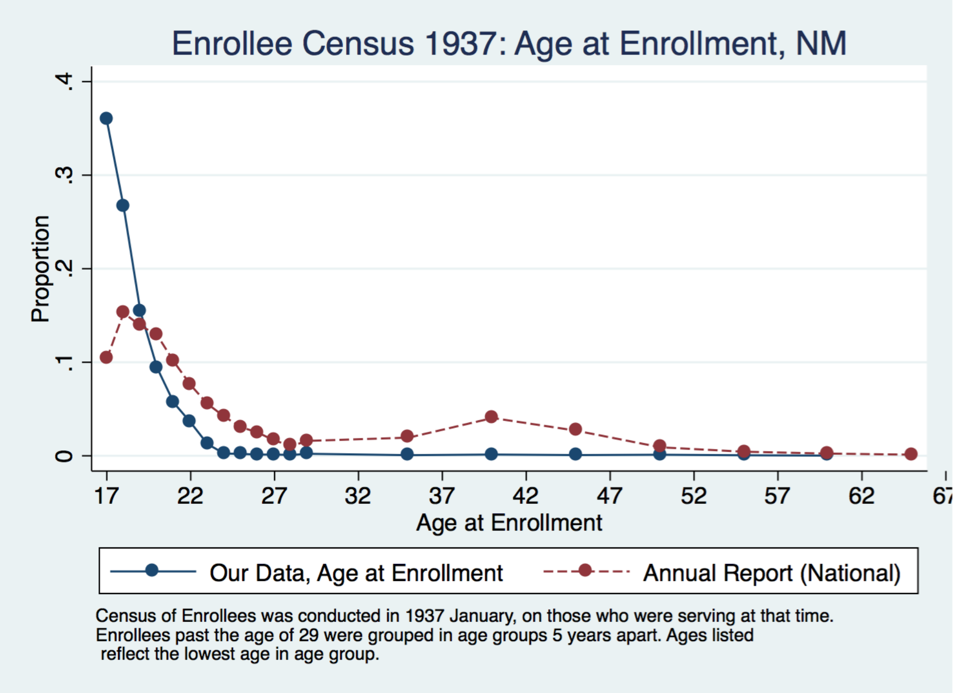

Panel B: Age at Enrollment, CO

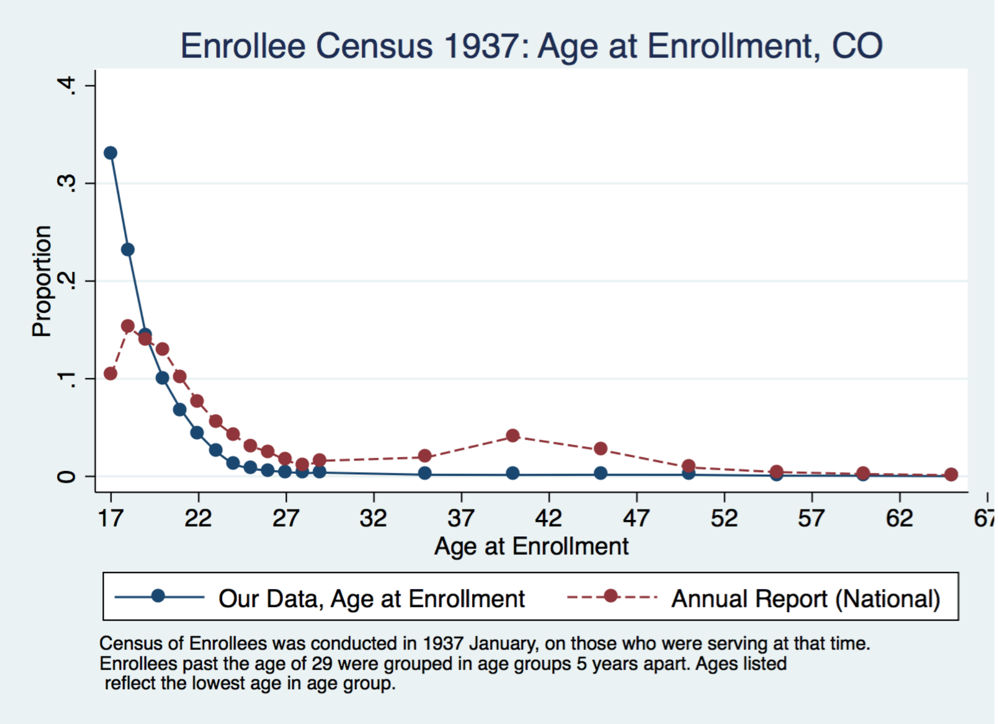

Panel C: Duration, CO Present in 1937 January

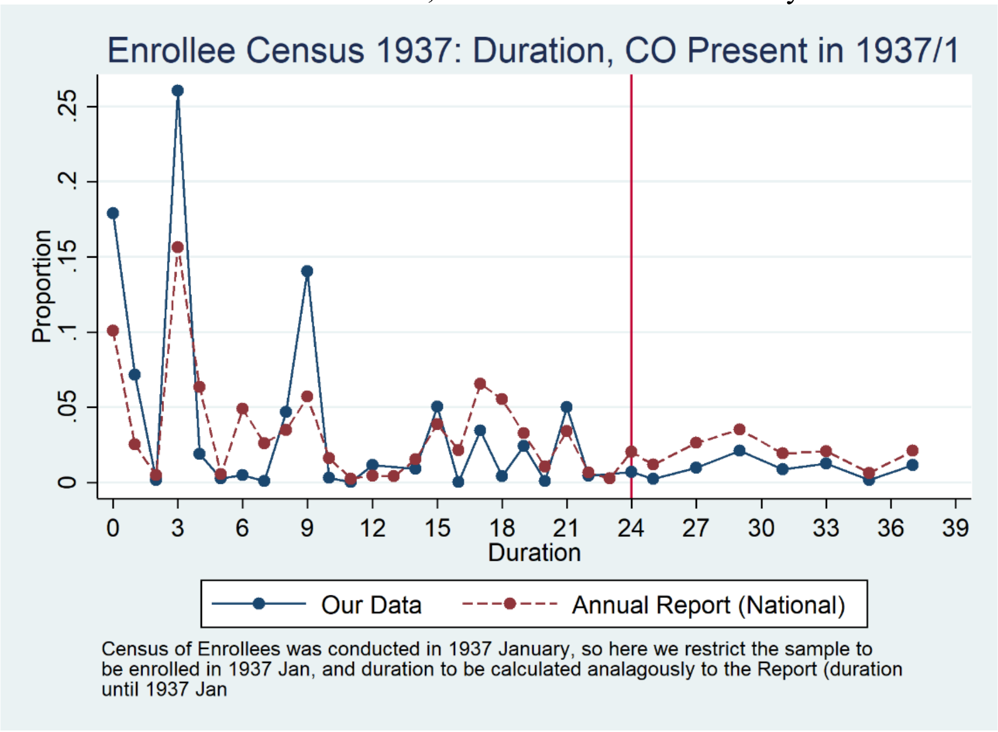

Panel D: Cumulative Distribution of Height, CO Present in 1939 January

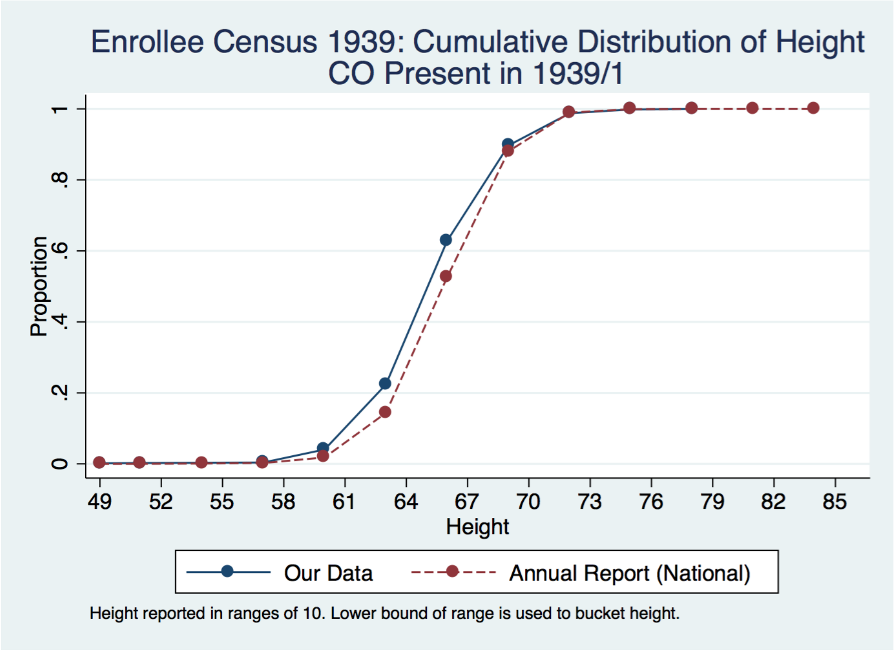

Panel E: Cumulative Distribution of Number of Dependents, CO Present in 1939 January

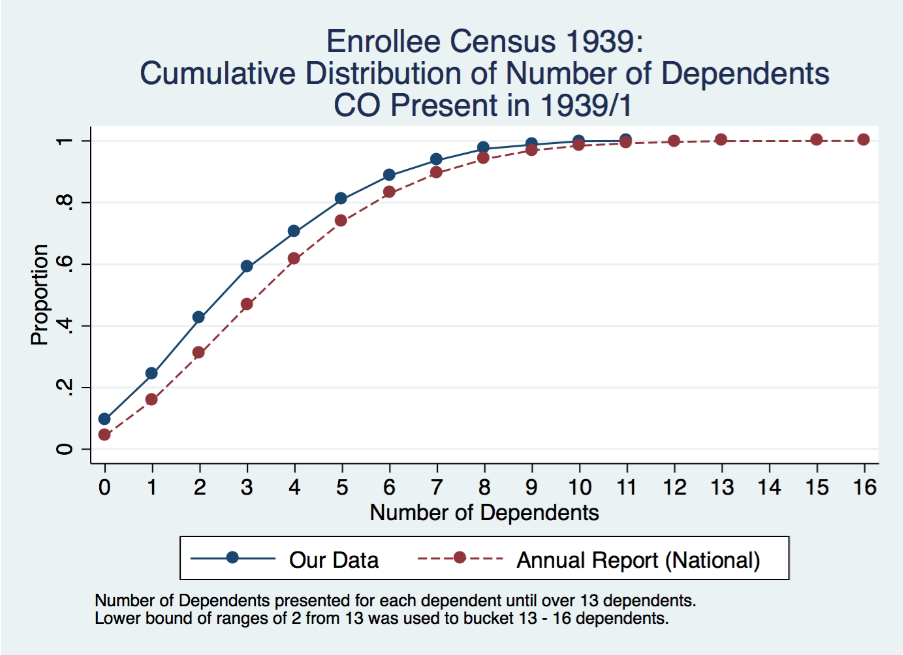

Panel F: Cumulative Distribution of School Graduate, CO Present in 1937 January

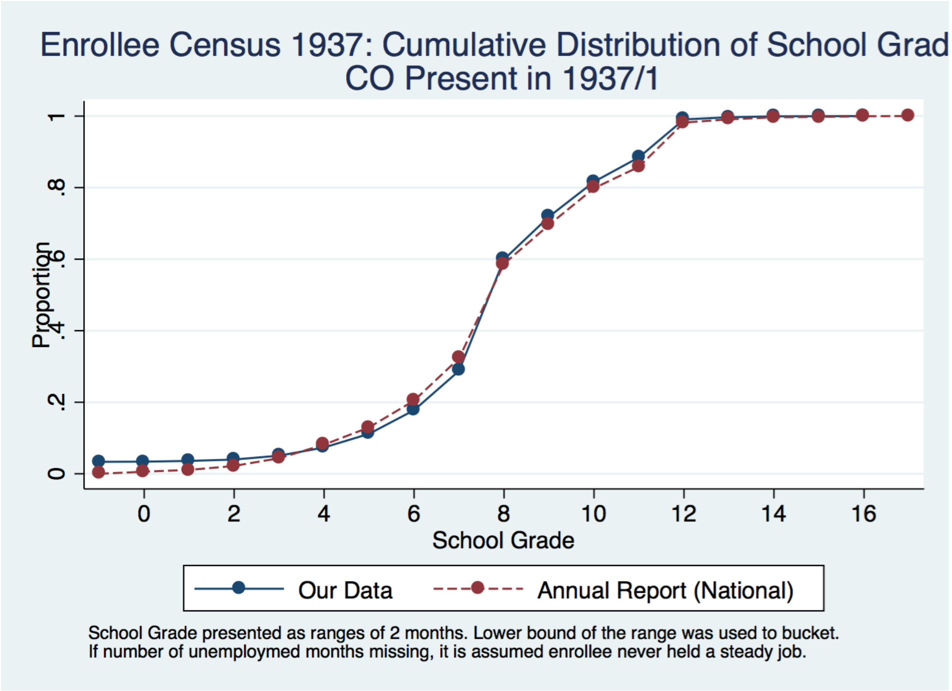

Figure A.IV

Cohort Eligibility and Participation in CCC

Panel A: Variation in Cohort Eligibility During the Years CCC Operated

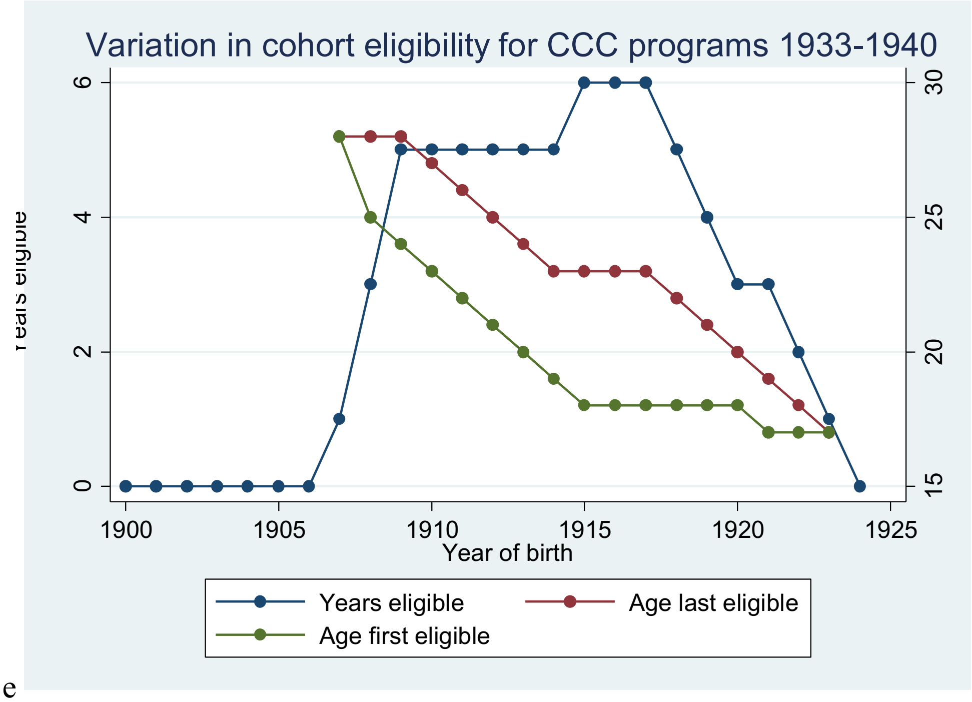

e

Panel B: Cohort participation in CO and NM CCC records

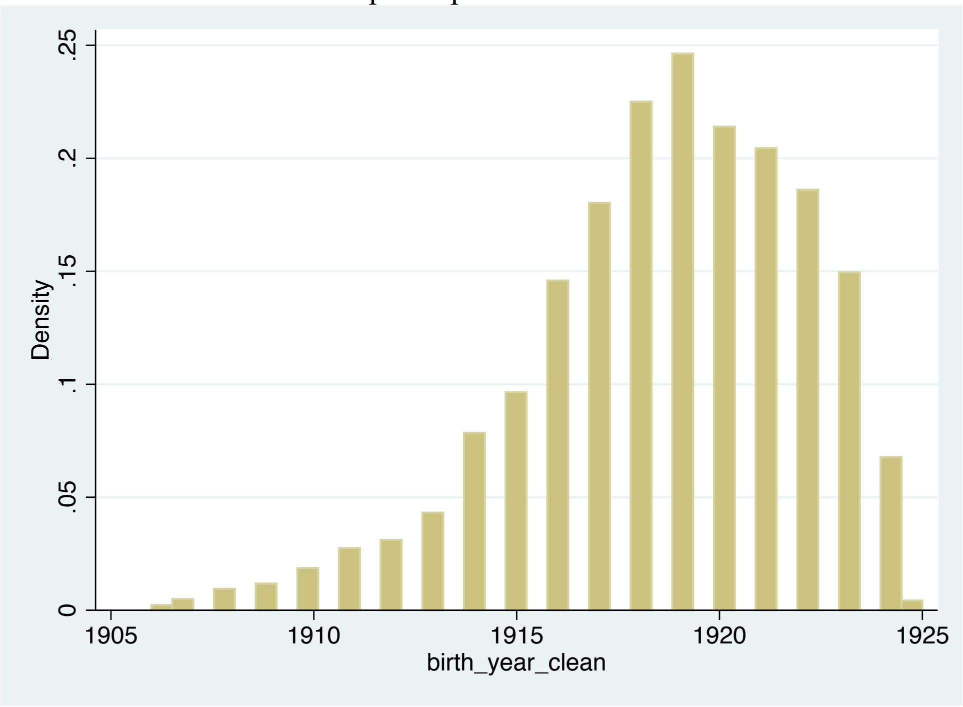

Figure A.V
Binscatter With Optimal Bins

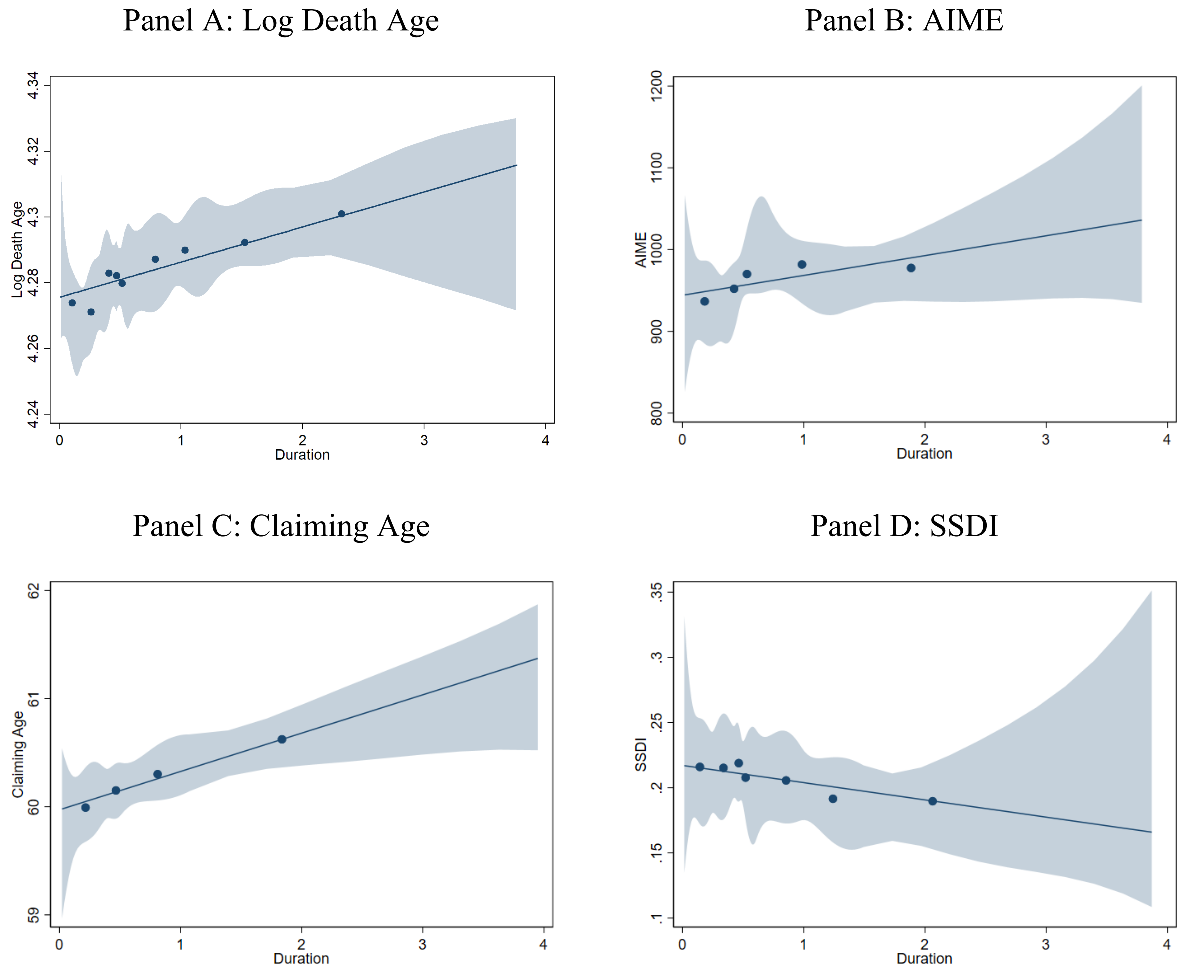

Notes: Authors computation based on death records (Panel A) and/or administrative program data matched to the Master Beneficiary Records (Panels B-D) and using the binscatter methodology of Cattaneo et al. 2023. It plots each variable controlling for birth year. We pick the polynomial and smoothness constraints for both confidence band and bin means at 1 and implement direct-plug-in data-driven choice of the optimal number of bins.

Figure A.VI

Distribution of Duration by Reason

Panel A: End of Term

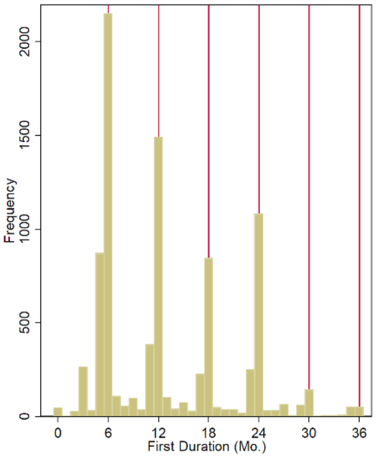

Panel B: Employment

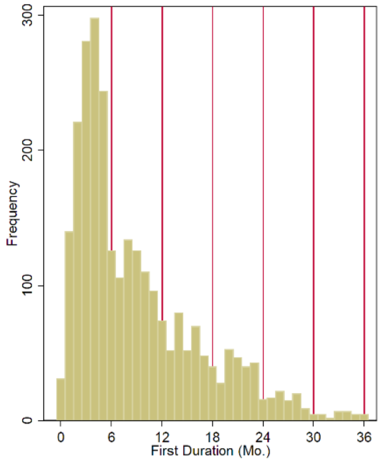

Panel C: Urgent/Proper Call

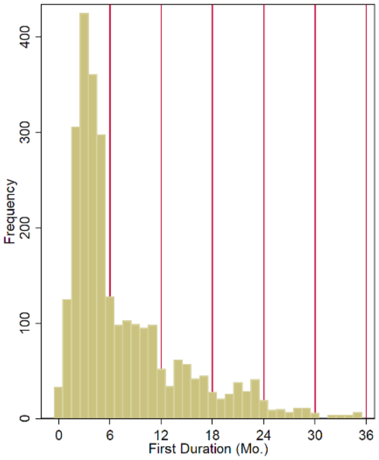

Panel D: Convenience of the Government

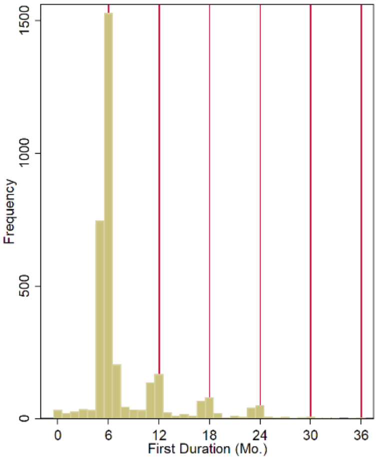

Panel E: Desertion

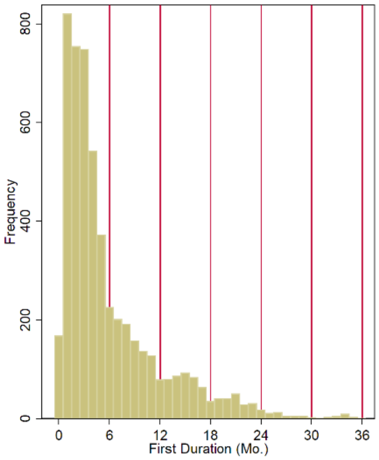

Notes: Histogram of duration in months by reason of discharge. We exclude duration longer than 3 years in this sample. Each red line represents increments of 6 months, which is the length of a completed term.

Figure A.VII
Example Colorado Enrollment Record

Form [illegible]C-S 210

10-759

CERTIFICATE OF SELECTION

For Enrollment in the

CIVILIAN CONSERVATION CORPS

Date 10-3-40

APPLICANT'S NAME Aragon (Last name) Aaron (First name) None (Middle name)

ADDRESS 511 West 4th St

POST OFFICE Walsenburg

STATE, COLORADO. COUNTY Huerfano

Application received by—

Huerfano County

Department of Public Welfare.

ADDRESS Court House

Walsenburg (City or Town) Colorado.

SECTION 1.

Age 20 Place and date of birth Gardner Colorado (City and State) July (Month) 4th (Day) 1920 (Year)

If not born in the United States, have you been naturalized? .... First papers .... (Date) Final papers .... (Place) (Date)

Height 71 in (Minimum: 60 in.) Weight 140 (Minimum: 107 lb.) Color of eyes brown Color of hair black

Applicant's marital status single Is your father living? yes (Yes or no) Mother living? yes (Yes or no)

How many brothers? 2 Sisters? 1 Occupation of principal wage earner of family? miner

How many members of your family reside in the same household with you? (excluding applicant) 5 (Number)

Do you live on a farm? no (Yes or no) If so, is the farm owned by your family? .... (Yes or no)

Do you live in a town or village of less than 2,500 persons, or in a rural area, and *not on a farm*? no (Yes or no)

Do you live in a town or city of 2,500 or more persons? yes (Yes or no) If so, give population 7,000

How long have you resided in this State? 20 (Years) This county? 20 (Years) Population of county 15,901

STATE OF COLORADO RECEIVED OCT 15 1940 DEPT. [illegible]

SECTION 2.

School last attended Hill School (Name of school) Located at Walsenburg Colo (City and State) Date of leaving 1936

Education: { *Circle highest grade completed* } Grammar or grade school, 1 2 3 4 5 6 7 8. High school, 1 2 3 4. College, 1 2 3 4

Special educational or vocational interests General laborer

SECTION 3.

Are you now unemployed? yes (Yes or no) How long unemployed? 48 (Months) Do you need employment? yes (Yes or no)

Have you ever had a paid regular job? no (Yes or no) If so, give date last job ended .... Social Security Account No. 523-16-0392

Registered with State Employment Service? no (Yes or no) Work best qualified for farm laborer

If previously employed, give consecutive statement of your work history in space below (*list latest job at top*):

Name and Address of Employer	Nature of Work Performed	Inclusive Dates of Employment From—	To—
1. None			
2.			
3.			
4.			
5.			

Total months of all paid regular employment to date ....

SECTION 4.

Applicant's reason(s) for desiring C. C. C. enrollment:

Unemployed

(This form to be completed on reverse side)

SECTION 5.

Previously enrolled in C. C. C.? no (Yes or no) C. C. C. serial number .... If so, list all previous service below:

Company Number	Length of Service		Date Enrolled	Date Discharged	Type of Discharge Hon., Adm., or Dishon
	Months	Days			
1.					
2.					
3.					

Total length of all previous service in Civilian Conservation Corps: Months .... Days ....

SECTION 6. **DESIGNATION OF ALLOTTEE**

(Required for all juniors having dependents. Juniors without dependents will use Section 7)

Allotment from monthly cash allowance desired by applicant to be made to dependent(s) as follows:

Name Aragon (Last name) Margaret Mrs (First name) None (Middle name) Relationship Mother

Address 511 West 4th, Walsenburg Colo Amount per month $22.00

Name .... (Last name) (First name) (Middle name) Relationship ....

Address .... Amount per month ....

In addition to allotment, applicant desires deposit in the amount of $............ per month.

SECTION 7. **AUTHORIZATION FOR DEPOSIT IN LIEU OF ALLOTMENT**

(Completion of this Section required in all cases in which Section 6 is not used)

I. From the Selecting Agency: It is hereby certified, pursuant to regulations issued under section 9 of the Act to establish the Civilian Conservation Corps effective July 1, 1937, that through verification of the status of the applicant named herein, proper assurance has been obtained that he does not have any dependent member or members of his family to whom an allotment can be made. In order to be selected and enrolled in the Corps he is therefore required to agree to make a monthly deposit of pay in the amount of $............ with the Chief of Finance, War Department, to be repaid normally upon completion of or release from enrollment.

Selecting Agent's signature (*ink*) ....

II. From the Applicant: In accordance with the aforementioned Act and regulations prescribed thereunder by the Director of the Corps, I hereby certify that I do not have any dependent member or members of my family to whom an allotment of pay can be made, and I agree to make a monthly deposit of pay with the Chief of Finance, War Department, in the amount specified above, to be repaid normally upon completion of or release from enrollment.

Applicant's signature (*ink*) ....

SECTION 8.

The statements contained in the foregoing Sections are true, to the best of my knowledge. I desire to be enrolled in the Civilian Conservation Corps for a period of 6 months unless earlier released in accord with law and established regulations. If I am accepted and enrolled, I agree to abide faithfully by the rules and regulations of the Corps and am willing to be assigned to any C. C. C. camp within the continental United States.

Applicant's signature (*ink*) [signature]

SECTION 9. **THE OFFICE OF THE DIRECTOR (Division of Selection) C. C. C.**

Certifies that the above-named applicant has been properly selected for enrollment as a Junior in the Civilian Conservation Corps. For completion of his enrollment, including physical examination, he has been directed to report to C. C. C. acceptance officers at Walsenburg Colorado on October 10th, 19 40 at 9:00 a. m./p. m.

COLORADO STATE DEPARTMENT OF PUBLIC WELFARE
EARL M. KOUNS, DIRECTOR
STATE CAPITOL ANNEX
DENVER, COLORADO

Routing of Copies:

To Army—white copy.
To State Department of Public Welfare—yellow copy.
To County Files—pink copy.

By Cynthia W. Haines (Ink signature of authorized selecting agent)

Director, Huerfano County DPW October 9th, 1940 (Official designation)

Figure A.VIII
Example New Mexico Discharge Record

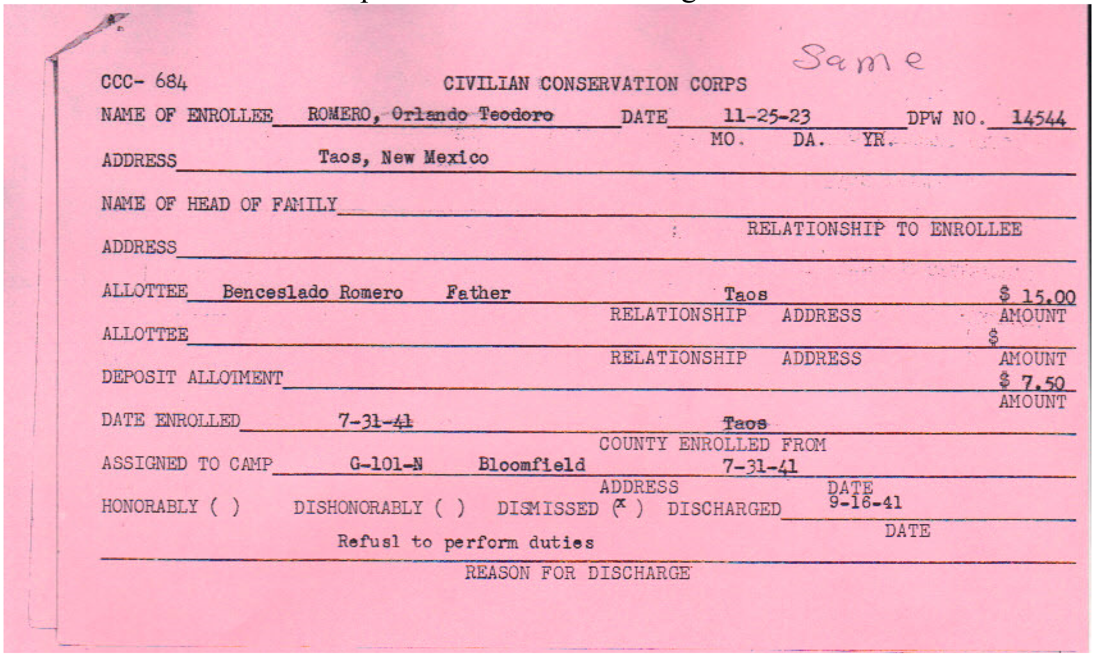

Same

CCC- 684 CIVILIAN CONSERVATION CORPS

NAME OF ENROLLEE ROMERO, Orlando Teodoro DATE 11-25-23 DPW NO. 14544

MO. DA. YR.

ADDRESS Taos, New Mexico

NAME OF HEAD OF FAMILY

RELATIONSHIP TO ENROLLEE

ADDRESS

ALLOTTEE Benceslado Romero Father Taos $ 15.00

RELATIONSHIP ADDRESS AMOUNT

ALLOTTEE $

RELATIONSHIP ADDRESS AMOUNT

DEPOSIT ALLOTMENT $ 7.50

AMOUNT

DATE ENROLLED 7-31-41 Taos

COUNTY ENROLLED FROM

ASSIGNED TO CAMP G-101-N Bloomfield 7-31-41

ADDRESS DATE

HONORABLY ( ) DISHONORABLY ( ) DISMISSED (x) DISCHARGED 9-16-41

DATE

Refusl to perform duties

REASON FOR DISCHARGE

H. Appendix Tables

Table A.I: Sample Selection

Sample Restriction	Itself	Sequential
All	26290	26290
Camp Exist	25165	25165
Enrollment Exist	24832	23943
Duration Exist	26050	23722
**Final analytic sample**	**23722**	**23722**
Death Age Exist	21457	19377
Death Age Restrict	24386	17639
Camp Controls Exist	24580	17237
Peer Controls Exist	24546	17086
**Final analytic sample for mortality**	**24546**	**17086**

Notes: The rows show many observations survive after dropping for each restriction. Itself column shows how many observations survive if we drop for just the restriction in the row. Sequential column shows the final observations that survive when we drop for each reason sequentially. Our working sample is 23,722, where we additionally lose observations to Death Age Exist for death age analysis, resulting in a sample of 17,086.

Table A.IIa: Summary Statistics From Enrollment Records

	Analytic Sample			Mortality Sample			Analytic Sample (matched to MBR)		
	N	mean	sd	N	mean	sd	N	mean	sd
**Characteristics in Enrollment Application**									
Birth year	23,722	1920	3.712	17,086	1920	3.590	12,455	1920	3.546
Age at enrollment	23,488	18.75	2.122	16,908	18.73	2.163	12,330	18.74	2.242
Enrollment year	23,722	1939	1.902	17,086	1939	1.883	12,455	1939	1.889
Reported age younger than DMF*	23,722	0.0888	0.284	17,086	0.114	0.317	12,455	0.130	0.336
Reported age older than DMF*	23,722	0.167	0.373	17,086	0.218	0.413	12,455	0.253	0.435
Age is 17 or 18	23,488	0.564	0.496	16,908	0.535	0.499	12,330	0.513	0.500
Not Eligible	23,722	0.0151	0.122	17,086	0.0137	0.116	12,455	0.0139	0.117
Allottee is father	23,722	0.334	0.472	17,086	0.333	0.471	12,455	0.330	0.470
Allottee is mother	23,722	0.466	0.499	17,086	0.475	0.499	12,455	0.475	0.499
Non-junior	23,722	0.00628	0.0790	17,086	0.00673	0.0818	12,455	0.00674	0.0818
Hispanic (imputed using hispanic index)	23,722	0.484	0.500	17,086	0.450	0.498	12,455	0.432	0.495
Additional information in CO records									
Highest grade completed	14,507	8.592	2.109	10,828	8.685	2.071	8,225	8.700	2.055
Household size excluding applicant	7,870	4.745	2.600	6,090	4.772	2.590	4,730	4.725	2.575
Live on farm?	8,101	0.248	0.432	6,259	0.255	0.436	4,846	0.252	0.434
Height (Inches)	8,141	67.80	3.089	6,272	67.90	3.080	4,860	67.92	3.053
Weight (100 pounds)	8,234	1.385	0.171	6,356	1.390	0.171	4,922	0.0139	0.00171
Body Mass Index	8,115	21.21	2.178	6,258	21.22	2.170	4,849	21.23	2.190
Underweight	8,115	0.0694	0.254	6,258	0.0684	0.252	4,849	0.0685	0.253
Overweight	8,115	0.0450	0.207	6,258	0.0455	0.209	4,849	0.0462	0.210
Father Living	7,943	0.799	0.401	6,146	0.805	0.396	4,765	0.806	0.396
Mother Living	8,006	0.850	0.357	6,196	0.857	0.350	4,808	0.855	0.352
Tenure in county (years)	5,432	12.66	6.483	4,184	12.69	6.504	3,353	12.59	6.522
Ever had a paid regular job?	8,841	0.375	0.484	6,801	0.384	0.486	5,256	0.394	0.489
Male White Unemployed / Male White Pop 1937	23,709	0.0885	0.0397	17,077	0.0866	0.0389	12,450	0.0850	0.0378
Male White Unemployed / Male White Pop 1940	23,709	0.0710	0.0308	17,077	0.0697	0.0300	12,450	0.0688	0.0291
**Service Characteristics**									
First allottee amount (dollars per month)	22,970	21.63	3.772	16,566	21.69	3.687	12,097	21.70	3.683
Duration of service (yrs)	23,722	0.821	0.706	17,086	0.836	0.707	12,455	0.816	0.701
Ever Rejected?	23,722	0.0194	0.138	17,086	0.0156	0.124	12,455	0.0199	0.140
=1 if disabled	23,722	0.00847	0.0917	17,086	0.00696	0.0832	12,455	0.00690	0.0828
Gap in service (more than 3 months)	23,722	0.160	0.366	17,086	0.173	0.378	12,455	0.180	0.384
Reason ended: End of term	23,722	0.379	0.485	17,086	0.379	0.485	12,455	0.372	0.483
Reason ended: Employment	23,722	0.116	0.320	17,086	0.124	0.330	12,455	0.125	0.331
Reason ended: Convenience of the government	23,722	0.145	0.352	17,086	0.150	0.358	12,455	0.154	0.361
Reason ended: Urgent and Proper Call	23,722	0.117	0.321	17,086	0.124	0.329	12,455	0.125	0.330
Reason ended: Deserted	23,722	0.222	0.416	17,086	0.207	0.405	12,455	0.205	0.404
Reason ended: Rejected upon examination	23,722	0.00915	0.0952	17,086	0.00304	0.0551	12,455	0.00690	0.0828
Reason ended: No Record	23,722	0.0128	0.112	17,086	0.0119	0.108	12,455	0.0120	0.109
Honorable Discharge	23,722	0.767	0.423	17,086	0.788	0.408	12,455	0.786	0.410
**Camp Characteristics**									
Distance from home to camp in miles (derived)	22,405	154.8	207.1	16,402	157.2	208.5	11,740	159.5	209.1
1st closest city distance form camp (miles)	23,480	26.68	22.50	17,086	26.61	22.29	12,322	26.40	22.06
2nd closest city distance form camp (miles)	23,480	49.86	22.49	17,086	49.21	22.37	12,322	48.71	22.17
Mean precipitation in camp 1933-1942	23,202	33.43	9.281	17,086	33.53	9.316	12,174	33.66	9.382
Mean min temp in camp 1933-1942	23,202	1.459	3.474	17,086	1.389	3.449	12,174	1.265	3.450
Mean max temp in camp 1933-1942	23,202	17.51	4.114	17,086	17.40	4.107	12,174	17.24	4.106
Camp Mean Hispanic (imputed using hispanic index)	23,722	0.482	0.313	17,086	0.463	0.309	12,455	0.430	0.329
Camp Type: Department of Grazing	23,671	0.135	0.341	17,086	0.133	0.339	12,455	0.131	0.337
Camp Type: Federal Reclamation Project	23,671	0.0553	0.229	17,086	0.0574	0.233	12,455	0.0560	0.230
Camp Type: Fish and Wildlife Service	23,671	0.0118	0.108	17,086	0.0115	0.106	12,455	0.0106	0.102
Camp Type: National Forest	23,671	0.295	0.456	17,086	0.292	0.455	12,455	0.292	0.454
Camp Type: National Monument	23,671	0.0191	0.137	17,086	0.0166	0.128	12,455	0.0188	0.136
Camp Type: National Park	23,671	0.105	0.307	17,086	0.109	0.312	12,455	0.108	0.310
Camp Type: Soil Conservation	23,671	0.307	0.461	17,086	0.315	0.464	12,455	0.306	0.461
Camp Type: State Park	23,671	0.0524	0.223	17,086	0.0475	0.213	12,455	0.0540	0.226
Camp Type: Other	23,671	0.0202	0.141	17,086	0.0189	0.136	12,455	0.0214	0.145

Notes: Basic sample includes records with duration (begin and end date of enrollment), camp id and enrollment county. The analytical sample for the mortality analysis only includes those not missing death age and death age more than 45. When multiple records were found for a single individual we use the information in the first enrollment record. *Reported age being younger (older) than DMF OR than the oldest (youngest) reported if the individual has multiple enrollment spells.

Table A.IIb: Summary Statistics From Death Certificate, 1940 and WWII Records

	Analytic Sample			Analytic Sample for mortality Analysis			Analytic Sample (MBR matched)		
	N	mean	sd	N	mean	sd	N	mean	sd
**Death Certificate Data**									
Age at death	19,377	69.82	16.84	17,086	73.62	12.03	12,348	74.76	9.245
=1 if missing age at death	23,722	0.183	0.387	17,086	0	0	12,455	0.00859	0.0923
Survive at 70	19,377	0.587	0.492	17,086	0.645	0.479	12,348	0.706	0.456
P(70), imputed to 0 if missing	23,722	0.479	0.500	17,086	0.645	0.479	12,455	0.700	0.458
Imputed Prob of Survival at 70 Using Age at Discharge	23,718	0.589	0.446	17,086	0.645	0.479	12,455	0.705	0.454
**1940 Census Data**									
Matched to 1940 Census	23,722	0.449	0.497	17,086	0.479	0.500	12,455	0.487	0.500
Panel a: those that served before 1940									
Matched to 1940 Census	9,890	0.433	0.496	7,025	0.473	0.499	5,151	0.483	0.500
Year of birth	4,216	1918	3.833	3,277	1918	3.693	2,451	1918	3.559
Age at last birthday (in years)	4,216	21.77	3.833	3,277	21.71	3.693	2,451	21.74	3.559
Hispanic	4,216	0.279	0.449	3,277	0.254	0.435	2,451	0.245	0.430
White	4,216	0.991	0.0933	3,277	0.992	0.0870	2,451	0.991	0.0922
In labor force	4,216	0.909	0.288	3,277	0.912	0.283	2,451	0.909	0.288
Working, conditional on labor force	3,832	0.712	0.453	2,990	0.718	0.450	2,228	0.711	0.453
Wage, conditional on working	2,982	405.2	361.0	2,326	404.1	339.6	1,764	410.8	360.7
Lives in CO	4,216	0.776	0.417	3,277	0.783	0.412	2,451	0.790	0.407
Lives in NM	4,216	0.166	0.372	3,277	0.156	0.363	2,451	0.144	0.351
Years of educ	4,158	8.770	2.477	3,230	8.857	2.431	2,415	8.873	2.420
Moved Residence Counties	4,214	0.299	0.458	3,275	0.294	0.456	2,450	0.296	0.457
Panel b: those that served after 1940									
Matched to 1940 Census	12,540	0.456	0.498	9,034	0.480	0.500	6,499	0.486	0.500
Year of birth	5,608	1922	3.138	4,258	1922	2.827	3,100	1922	2.763
Age at last birthday (in years)	5,608	17.91	3.138	4,258	17.91	2.827	3,100	17.91	2.763
Hispanic	5,608	0.443	0.497	4,258	0.419	0.493	3,100	0.394	0.489
White	5,608	0.988	0.107	4,258	0.992	0.0903	3,100	0.989	0.104
In labor force	5,608	0.633	0.482	4,258	0.641	0.480	3,100	0.647	0.478
Working, conditional on labor force	3,550	0.687	0.464	2,730	0.695	0.460	2,006	0.683	0.465
Wage, conditional on working	2,289	249.0	273.9	1,781	253.5	290.9	1,325	258.8	319.7
Lives in CO	5,608	0.533	0.499	4,258	0.554	0.497	3,100	0.596	0.491
Lives in NM	5,608	0.452	0.498	4,258	0.430	0.495	3,100	0.390	0.488
Years of educ	5,554	7.987	2.430	4,219	8.084	2.407	3,068	8.155	2.373
Moved Residence Counties	5,608	0.142	0.349	4,258	0.137	0.344	3,100	0.135	0.342
**WWII Records**									
Matched to WWII records	23,722	0.306	0.461	17,086	0.338	0.473	12,455	0.347	0.476
Birth year	7,263	1920	2.810	5,781	1920	2.800	4,321	1920	2.815
Enrollment year	7,262	1942	1.424	5,781	1942	1.439	4,321	1942	1.450
Years of education	7,263	9.395	1.787	5,781	9.401	1.787	4,321	9.399	1.766
Height in inches*	5,971	67.52	6.089	4,740	67.71	6.090	3,510	67.73	6.164
Weight in lbs**	5,641	138.6	26.19	4,470	138.7	25.49	3,327	139.4	27.17
BMI	5,466	21.55	4.500	4,330	21.48	3.797	3,214	21.55	4.399
Ever Married	7,256	0.215	0.411	5,774	0.220	0.414	4,316	0.224	0.417
Home State CO	7,232	0.591	0.492	5,756	0.602	0.490	4,300	0.617	0.486
Moved Residence Counties	7,215	0.303	0.460	5,742	0.289	0.453	4,290	0.303	0.460
Home State NM	7,232	0.319	0.466	5,756	0.309	0.462	4,300	0.289	0.453
Birthplace CO	7,215	0.444	0.497	5,740	0.448	0.497	4,295	0.462	0.499
Birthplace NM	7,215	0.322	0.467	5,740	0.312	0.463	4,295	0.292	0.455
Birthplace Rest of US	7,215	0.230	0.421	5,740	0.238	0.426	4,295	0.244	0.429

Notes: Basic sample includes records with duration (begin and end date of enrollment), camp id and enrollment county. The analytical sample for the mortality analysis only includes those not missing death age and death age more than 45. When multiple records were found for a single individual we use the information in the first enrollment record. * Dropped values below 40. ** Dropped values below 90 and over 350

Table A.III: Comparison of Counties of Enrollees vs Whole State

Year	1930				1940			
State	CO		NM		CO		NM	
Geography	State	CCC	State	CCC	State	CCC	State	CCC
Variables								
Share Urban	0.5	0.4	0.25	0.22	0.53	0.42	0.33	0.28
Share in Farm	0.27	0.33	0.37	0.38	0.22	0.28	0.32	0.35
Share Owns Home	0.5	0.5	0.59	0.64	0.47	0.48	0.61	0.65
Mean Rent	38.88	37.6	26.39	23.09	102.99	95.43	219.27	271.4
Mean Age	29.57	28.35	25.26	25.24	31.4	30.12	26.14	25.84
Share Male	0.51	0.52	0.52	0.52	0.51	0.51	0.51	0.51
Share White	0.98	0.99	0.92	0.95	0.99	0.99	0.93	0.96
Share Mexican	0.06	0.07	0.14	0.11	0.07	0.13	0.34	0.44
Share Ever Married	0.51	0.49	0.45	0.44	0.54	0.52	0.47	0.45
Share Students	0.24	0.25	0.25	0.25	0.21	0.23	0.25	0.26
Share Foreign-born	0.1	0.09	0.06	0.05	0.07	0.06	0.03	0.02
Mean Occscore	21.78	20.59	19.05	18.34	22.54	21.38	20.1	19.19
Share Employed	0.9	0.9	0.93	0.92	0.9	0.89	0.88	0.85
Mean Income					392.11	332.25	326.73	277.49
Mean Educ Years					7.75	7.25	5.86	5.45
Share Hisp Origin					0.08	0.13	0.34	0.44

Note: Columns "State" are the state average of variables in each row. Columns CCC is the weighted average of county characteristics, where the weights are the share of CCC enrollees in our data enrolling from each county.

Table A.IV: Determinants of CCC Service Duration

	(1)	(2)	(3)	(4)	(5)	(6)
VARIABLES	Indiv Controls only	Camp Controls only	Indiv+Camp	Add County-Quarter FE	CO Only	CO Non-missing Only
**Individual characteristics**						
Ever Rejected?	-0.201***		-0.020	-0.007	-0.009	0.060
	(0.033)		(0.034)	(0.031)	(0.034)	(0.038)
=1 if disabled	-0.446***		-0.464***	-0.328***	-0.363***	-0.237*
	(0.055)		(0.055)	(0.050)	(0.061)	(0.127)
Non-junior	0.834***		0.840***	0.509***	0.574***	0.005
	(0.122)		(0.119)	(0.097)	(0.127)	(0.235)
Reported Age Younger than DMF^	0.033*		0.026	0.003	0.003	-0.005
	(0.019)		(0.019)	(0.014)	(0.020)	(0.024)
Reported Age Older than DMF	0.081***		0.089***	-0.047***	-0.029*	-0.033
	(0.015)		(0.015)	(0.012)	(0.016)	(0.025)
Not Eligible	0.300**		0.265*	0.174**	0.186*	0.662***
	(0.139)		(0.141)	(0.077)	(0.106)	(0.134)
Age is 17 or 18	0.100***		0.103***	-0.037***	-0.045***	-0.020
	(0.014)		(0.014)	(0.011)	(0.014)	(0.021)
Allottee amount	0.058***		0.060***	-0.001	0.009	0.026***
	(0.004)		(0.005)	(0.004)	(0.006)	(0.009)
Allottee is father	0.045***		0.045***	0.001	0.001	-0.003
	(0.017)		(0.017)	(0.013)	(0.019)	(0.027)
Allottee is mother	0.045***		0.045***	0.017	0.030	0.012
	(0.017)		(0.016)	(0.014)	(0.019)	(0.027)
Gap in service	-0.201***		-0.156***	-0.158***	-0.126***	-0.113***
	(0.016)		(0.015)	(0.013)	(0.016)	(0.020)
Log distance from home to camp (miles)	-0.016***		-0.013**	-0.011**	-0.015***	-0.021**
	(0.005)		(0.005)	(0.005)	(0.006)	(0.008)
Hispanic (imputed using hispanic index)	0.078***		0.058***	0.026**	-0.014	0.007
	(0.014)		(0.014)	(0.013)	(0.017)	(0.019)
Highest grade completed (CO only)	0.024***		0.021***	0.019***	0.016***	0.007*
	(0.003)		(0.003)	(0.003)	(0.003)	(0.004)
Household size excluding applicant (CO only)	0.012***		0.013***	0.007***	0.008***	0.007***
	(0.003)		(0.003)	(0.002)	(0.002)	(0.003)
Live on farm? (CO only)	0.053***		0.053***	0.016	0.012	0.017
	(0.016)		(0.017)	(0.014)	(0.015)	(0.017)
Height (Inches) (CO only)	0.002		0.001	0.000	-0.000	-0.001
	(0.003)		(0.003)	(0.002)	(0.002)	(0.002)
Weight (100 pounds) (CO only)	-0.189***		-0.154***	-0.085*	-0.113**	-0.019
	(0.054)		(0.052)	(0.045)	(0.047)	(0.045)
Father Living (CO only)	-0.054***		-0.055***	-0.018	-0.015	-0.006
	(0.019)		(0.019)	(0.015)	(0.015)	(0.017)
Mother Living (CO only)	-0.088***		-0.095***	-0.051***	-0.056***	-0.032
	(0.021)		(0.021)	(0.016)	(0.017)	(0.024)
Tenure in county (years) (CO only)	-0.001		-0.001	-0.001	-0.001	-0.001
	(0.001)		(0.001)	(0.001)	(0.001)	(0.001)

**Camp characteristics**						
=1 if camp is in enrollment state		-0.094***	0.053	0.154***	0.165***	-0.027
		(0.034)	(0.051)	(0.058)	(0.059)	(0.066)
Mean precipitation in camp 1933-1942		-0.001	-0.001	-0.004***	0.001	0.001
		(0.001)	(0.001)	(0.001)	(0.002)	(0.003)
Mean min temp in camp 1933-1942		0.010	0.014**	0.030***	0.027***	0.012
		(0.006)	(0.006)	(0.008)	(0.008)	(0.010)
Mean max temp in camp 1933-1942		-0.018***	-0.021***	-0.034***	-0.022**	-0.006
		(0.006)	(0.006)	(0.007)	(0.009)	(0.011)
Camp Type: Department of Grazing		0.131***	0.123***	-0.075	0.117	-0.052
		(0.044)	(0.041)	(0.063)	(0.087)	(0.116)
Camp Type: Federal Reclamation Project		0.118**	0.099**	-0.055	0.147	0.031
		(0.047)	(0.045)	(0.070)	(0.096)	(0.120)
Camp Type: Fish and Wildlife Service		0.106**	0.024	-0.383***		
		(0.051)	(0.048)	(0.131)		
Camp Type: National Forest		0.008	-0.006	-0.106*	0.024	-0.091
		(0.043)	(0.041)	(0.060)	(0.078)	(0.109)
Camp Type: National Monument		0.145*	0.121	-0.303***	-0.265*	-0.166
		(0.088)	(0.084)	(0.090)	(0.147)	(0.179)
Camp Type: National Park		0.069	0.060	-0.117*	-0.012	-0.165
		(0.044)	(0.042)	(0.063)	(0.079)	(0.101)
Camp Type: Soil Conservation		0.121***	0.100***	-0.075	0.092	-0.070
		(0.040)	(0.038)	(0.059)	(0.080)	(0.108)
Camp Type: State Park		-0.031	-0.041	-0.119*	-0.078	-0.176
		(0.054)	(0.050)	(0.069)	(0.090)	(0.147)
Log distance to closest city (miles)		-0.007*	-0.007**	0.011**	0.000	0.022**
		(0.004)	(0.004)	(0.005)	(0.007)	(0.008)
Log distance to 2nd closest city (miles)		0.028	0.035*	-0.017	-0.044*	0.012
		(0.019)	(0.019)	(0.022)	(0.025)	(0.037)
Peer Char: Hispanic at enrollment		0.386***	0.239***	0.249***	0.015	0.051
		(0.044)	(0.047)	(0.070)	(0.071)	(0.098)
Peer Char: Age at enrollment		-0.200***	-0.235***	-0.319***	-0.313***	0.052
		(0.021)	(0.023)	(0.034)	(0.035)	(0.041)
Peer Char: Reported Age Younger than DMF		0.483***	0.381**	-0.607***	-0.579**	0.478*
		(0.170)	(0.169)	(0.211)	(0.254)	(0.262)
Peer Char: Reported Age Older than DMF		-0.276**	-0.452***	-1.025***	-0.814***	0.397
		(0.127)	(0.137)	(0.200)	(0.236)	(0.318)
Peer Char: Not Eligible (First enrollment)		1.861***	1.587***	1.349***	-0.295	1.949*
		(0.256)	(0.273)	(0.389)	(0.452)	(1.041)
Peer Char: Allottee amount		0.083***	0.030***	-0.255***	-0.360***	-0.305***
		(0.005)	(0.007)	(0.017)	(0.024)	(0.018)
Peer Char: Allottee: Father		-0.083	-0.120	0.019	-0.040	0.088
		(0.126)	(0.122)	(0.149)	(0.177)	(0.198)
Peer Char: Allottee: Mother		-0.163	-0.117	-0.032	-0.078	-0.221
		(0.126)	(0.128)	(0.133)	(0.147)	(0.202)
Peer Char: Gap in service		-0.931***	-0.692***	-0.652***	-0.156	-1.462***
		(0.098)	(0.099)	(0.133)	(0.140)	(0.191)
Constant	-1.457***	3.342***	2.800***	12.992***	14.686***	6.747***
	(0.458)	(0.518)	(0.569)	(0.868)	(0.991)	(0.807)
Observations	17,639	17,086	17,086	17,086	10,944	3,013
R-squared	0.181	0.160	0.222	0.574	0.482	0.465
Mean Dep	0.83	0.84	0.84	0.84	0.76	0.67
FE	BD	BD	BD	BD,CYQ	BD,CYQ	BD,CYQ
Sample	All	All	All	All	CO	CO
Reason	N	N	N	N	N	N
Number of County-Quarter Groups				1,789	1,231	477

Notes: Standard errors clustered at the level of county-by-year-quarter of enlistment in parentheses, *** p<0.01, ** p<0.05, * p<0.1. Only death age >= 45 are included in regression. Variables imputed if missing and missing dummies included. County Unemployment is from ICPSR compilation of County statistics from 1937 Census of Unemployment and 1940 Decenniel Census. Those values are given to enrollment years 1937, 1938 for 1937 Census and 1939-1942 for 1940 Census. ^ =1 if reported age in CCC documents is smaller than in the DMF, or maximum of all reported age for enrollee.

Table A.V: Full Regressions of Log Death Age on Duration

VARIABLES	(1)<br>No Controls	(2)<br>Add Birth, County-qtr Dummies	(3)<br>Add Indiv Controls	(4)<br>Add Camp Chars	(5)<br>Add Peer Chars	(6)<br>Add Camp FE	(7)<br>CO only
Duration of service (yrs)	0.013***	0.013***	0.011***	0.011***	0.013***	0.013***	0.013***
	(0.002)	(0.002)	(0.002)	(0.002)	(0.003)	(0.003)	(0.003)
Ever Rejected?			-0.031***	-0.031***	-0.031***	-0.030***	-0.030***
			(0.011)	(0.011)	(0.011)	(0.011)	(0.011)
=1 if disabled			-0.006	-0.006	-0.006	-0.004	-0.003
			(0.016)	(0.016)	(0.016)	(0.016)	(0.021)
Non-junior			0.002	0.004	0.003	-0.000	-0.036
			(0.018)	(0.019)	(0.019)	(0.019)	(0.025)
Reported age younger than DMF^			-0.019***	-0.019***	-0.019***	-0.019***	-0.010*
			(0.005)	(0.005)	(0.005)	(0.005)	(0.006)
Reported age older than DMF			-0.022***	-0.022***	-0.022***	-0.022***	-0.018***
			(0.004)	(0.004)	(0.004)	(0.004)	(0.005)
Not Eligible			0.010	0.011	0.010	0.011	0.014
			(0.017)	(0.017)	(0.017)	(0.017)	(0.022)
Age is 17 or 18			0.007*	0.007*	0.007*	0.007*	0.004
			(0.004)	(0.004)	(0.004)	(0.004)	(0.005)
First allottee amount (dollars per month)			0.000	0.000	0.000	0.000	0.000
			(0.001)	(0.001)	(0.001)	(0.001)	(0.001)
Allottee is father			0.008*	0.008*	0.008*	0.008	0.003
			(0.005)	(0.005)	(0.005)	(0.005)	(0.007)
Allottee is mother			0.001	0.001	0.001	0.001	-0.000
			(0.005)	(0.005)	(0.005)	(0.005)	(0.006)
Gap in service (more than 3 months)			0.001	0.001	0.001	0.001	-0.003
			(0.004)	(0.004)	(0.004)	(0.005)	(0.005)
Log distance from home to camp			0.001	0.002	0.002	0.002	0.002
			(0.001)	(0.001)	(0.001)	(0.001)	(0.002)
Hispanic (imputed using hispanic index)			0.018***	0.018***	0.018***	0.019***	0.018***
			(0.004)	(0.004)	(0.004)	(0.004)	(0.006)
Highest grade completed (CO only)			0.004***	0.004***	0.004***	0.005***	0.005***
			(0.001)	(0.001)	(0.001)	(0.001)	(0.001)
Household size excluding applicant (CO only			0.003***	0.003***	0.003***	0.003***	0.003***
			(0.001)	(0.001)	(0.001)	(0.001)	(0.001)
Live on farm? (CO only)			0.011*	0.011*	0.011*	0.011*	0.011**
			(0.006)	(0.006)	(0.006)	(0.006)	(0.006)
Height (Inches) (CO only)			0.001	0.001	0.001	0.001	0.001
			(0.001)	(0.001)	(0.001)	(0.001)	(0.001)
Weight (100 pounds) (CO only)			-0.042**	-0.041**	-0.041**	-0.041**	-0.042**
			(0.018)	(0.018)	(0.018)	(0.018)	(0.018)
Father Living (CO only)			0.000	0.001	0.000	-0.000	-0.000
			(0.006)	(0.006)	(0.006)	(0.006)	(0.006)
Mother Living (CO only)			0.008	0.008	0.008	0.008	0.007
			(0.007)	(0.007)	(0.007)	(0.007)	(0.007)
Tenure in county (years) (CO only)			-0.001	-0.001	-0.001	-0.001	-0.001
			(0.000)	(0.000)	(0.000)	(0.000)	(0.000)
=1 if camp is in enrollment state				-0.015	-0.017		
				(0.012)	(0.012)		
Mean precipitation in camp 1933-1942				0.000	0.000		
				(0.000)	(0.000)		
Mean min temp in camp 1933-1942				-0.002	-0.002		
				(0.001)	(0.002)		
Mean max temp in camp 1933-1942				0.000	0.001		
				(0.001)	(0.001)		

	(1)	(2)	(3)	(4)	(5)	(6)	(7)
Camp Type: Department of Grazing				-0.020	-0.019		
				(0.024)	(0.024)		
Camp Type: Federal Reclamation Project				-0.017	-0.019		
				(0.025)	(0.026)		
Camp Type: Fish and Wildlife Service				-0.012	-0.013		
				(0.032)	(0.033)		
Camp Type: National Forest				-0.015	-0.013		
				(0.024)	(0.025)		
Camp Type: National Monument				-0.006	-0.001		
				(0.028)	(0.028)		
Camp Type: National Park				-0.021	-0.017		
				(0.024)	(0.025)		
Camp Type: Soil Conservation				-0.010	-0.007		
				(0.024)	(0.024)		
Camp Type: State Park				-0.013	-0.012		
				(0.024)	(0.025)		
Log distance to closest city				-0.002**	-0.002**		
				(0.001)	(0.001)		
Log distance to 2nd closest city				0.003	0.005		
				(0.006)	(0.006)		
Peer Char: Hispanic at enrollment					0.002	-0.024	-0.009
					(0.014)	(0.021)	(0.023)
Peer Char: Age at enrollment					0.011**	0.014**	0.012
					(0.005)	(0.006)	(0.007)
Peer Char: Reported Age Younger than DMF					0.006	-0.031	-0.057
					(0.043)	(0.057)	(0.066)
Peer Char: Reported Age Older than DMF					-0.017	-0.007	-0.056
					(0.029)	(0.037)	(0.040)
Peer Char: Not Eligible (First enrollment)					-0.029	-0.070	-0.190*
					(0.051)	(0.077)	(0.098)
Peer Char: Allottee amount					0.002	-0.000	0.004
					(0.002)	(0.003)	(0.004)
Peer Char: Allottee: Father					-0.050*	-0.079**	-0.077*
					(0.030)	(0.038)	(0.044)
Peer Char: Allottee: Mother					-0.004	0.003	0.020
					(0.025)	(0.031)	(0.036)
Peer Char: Gap in service					-0.025	-0.026	0.010
					(0.026)	(0.033)	(0.034)
Constant	4.274***	4.391***	4.308***	4.294***	4.063***	4.363***	4.306***
	(0.002)	(0.137)	(0.159)	(0.168)	(0.206)	(0.162)	(0.182)
Observations	17,086	17,086	17,086	17,086	17,086	17,086	10,944
R-squared	0.003	0.117	0.126	0.127	0.128	0.138	0.147
Mean Dep	73.62	73.62	73.62	73.62	73.62	73.62	73.30
FE	None	BD,CYQ	BD,CYQ	BD,CYQ	BD,CYQ	BD,CYQ,Camp	BD,CYQ,Camp
Sample	All	All	All	All	All	All	CO
Number of County-Quarter Groups		1,789	1,789	1,789	1,789	1,789	1,231

Notes: Standard errors clustered at the level of county-by-year-quarter of enlistment in parentheses, *** p<0.01, ** p<0.05, * p<0.1. Sample is restricted only to those that died after age >= 45 and have non-missing peer and camp characteristics. Column (1) includes only duration of service as regressor. Column (2) adds Birth and County-Year-Quarter of Enrollment fixed effects. Column (3) adds individual controls. Column (4) adds camp characteristics, such as distance from nearest city and average temperature. Column (5) adds peer characteristics, where peers are defined as other enrollees serving in the same camp at the same time. Column (6) adds camp fixed effects and removes camp characteristics. Column (7) runs the regression specification in Column (6) for only enrollees from our Colorado Records.

Table A.VI: Robustness to Clustering
Effect of Service Duration on Longevity and Lifetime Earnings

	(1)	(2)	(3)	(4)	(5)	(6)	(7)
Depdent variable	No Controls	Add Birth, County-qtr Dummies	Add Indiv Controls	Add Camp Chars	Add Peer Chars	Add Camp FE	CO Only
**Panel A: Longevity for the full sample (log death age)**							
Duration of service (yrs)	0.013***	0.013***	0.011***	0.011***	0.013***	0.013***	0.013***
Standard Errors Clusterted at...							
County-by-Year-Quarter	(0.002)	(0.002)	(0.002)	(0.002)	(0.003)	(0.003)	(0.003)
County	(0.001)	(0.002)	(0.002)	(0.002)	(0.003)	(0.003)	(0.003)
Year-Quarter	(0.002)	(0.002)	(0.002)	(0.002)	(0.002)	(0.003)	(0.003)
No Clustering	(0.002)	(0.003)	(0.003)	(0.003)	(0.003)	(0.003)	(0.004)
**Panel B: AIME (MBR sample claimed 1979 and later)**							
Duration of service (yrs)	-0.083	67.178***	62.791***	62.450***	56.717***	50.134***	48.707***
Standard Errors Clusterted at...							
County-by-Year-Quarter	(10.181)	(12.186)	(12.501)	(12.616)	(13.723)	(14.690)	(17.236)
County	(10.754)	(13.065)	(13.515)	(13.861)	(13.960)	(14.454)	(17.699)
Year-Quarter	(12.329)	(14.323)	(14.750)	(15.258)	(14.968)	(16.138)	(18.180)
No Clustering	(9.563)	(12.562)	(12.840)	(12.889)	(14.378)	(15.555)	(18.481)
**Panel C: Retirement age**							
Duration of service (yrs)	0.506***	0.507***	0.452***	0.462***	0.427***	0.401***	0.554***
Standard Errors Clusterted at...							
County-by-Year-Quarter	(0.065)	(0.086)	(0.089)	(0.089)	(0.097)	(0.107)	(0.124)
County	(0.066)	(0.088)	(0.080)	(0.079)	(0.090)	(0.110)	(0.121)
Year-Quarter	(0.086)	(0.090)	(0.096)	(0.096)	(0.098)	(0.097)	(0.107)
No Clustering	(0.069)	(0.093)	(0.094)	(0.095)	(0.105)	(0.114)	(0.127)
**Panel D: SSDI (excluding unknowns)**							
Duration of service (yrs)	-0.016**	-0.022***	-0.020**	-0.021**	-0.017*	-0.021**	-0.031***
Standard Errors Clusterted at...							
County-by-Year-Quarter	(0.006)	(0.008)	(0.009)	(0.009)	(0.009)	(0.010)	(0.012)
County	(0.007)	(0.009)	(0.008)	(0.009)	(0.010)	(0.011)	(0.013)
Year-Quarter	(0.008)	(0.010)	(0.010)	(0.010)	(0.008)	(0.008)	(0.010)
No Clustering	(0.006)	(0.008)	(0.009)	(0.009)	(0.010)	(0.010)	(0.012)

Notes: Standard errors clustered at the level noted, *** p<0.01, ** p<0.05, * p<0.1. Sample is restricted only to those that died after age >= 45 and have non-missing peer and camp characteristics. Column (1) includes only duration of service as regressor. Column (2) adds Birth and County-Year-Quarter of Enrollment fixed effects. Column (3) adds individual controls. Column (4) adds camp characteristics, such as distance from nearest city and average temperature. Column (5) adds peer characteristics, where peers are defined as other enrollees serving in the same camp at the same time. Column (6) adds camp fixed effects and removes camp characteristics. Column (7) runs the regression specification in Column (6) for only enrollees from our Colorado Records. For complete list of controls, refer to text or Appendix Table V.

Table A.VII: Robustness to Various Cohort FE

Effect of Service Duration on Longevity and Lifetime Earnings

	(1)	(2)	(3)	(4)	(5)	(6)	(7)
Depdent variable	No Controls	Add FE	Add Indiv Controls	Add Camp Chars	Add Peer Chars	Add Camp FE	CO Only
**Panel A: Longevity for the full sample (log death age)**							
County-Year-Quarter and Cohort FE	0.013***	0.013***	0.011***	0.011***	0.013***	0.013***	0.013***
	(0.002)	(0.002)	(0.002)	(0.002)	(0.003)	(0.003)	(0.003)
Replace Cohort with Cohort-State FE	0.013***	0.013***	0.012***	0.012***	0.014***	0.013***	0.013***
	(0.002)	(0.002)	(0.002)	(0.002)	(0.003)	(0.003)	(0.003)
Replace Cohort with Cohort-County FE	0.013***	0.013***	0.011***	0.012***	0.014***	0.012***	0.013***
	(0.002)	(0.002)	(0.002)	(0.002)	(0.003)	(0.003)	(0.004)
**Panel B: AIME (MBR sample claimed 1979 and later)**							
County-Year-Quarter and Cohort FE	-0.083	67.048***	62.791***	62.450***	56.717***	50.134***	48.707***
	(10.181)	(12.186)	(12.501)	(12.616)	(13.723)	(14.690)	(17.236)
Replace Cohort with Cohort-State FE	-0.083	67.119***	63.330***	62.940***	57.639***	51.717***	48.707***
	(10.181)	(12.162)	(12.455)	(12.565)	(13.627)	(14.584)	(17.236)
Replace Cohort with Cohort-County FE	-0.083	71.924***	67.701***	67.174***	61.790***	54.425***	50.783***
	(10.181)	(12.538)	(12.933)	(13.020)	(14.176)	(15.073)	(17.896)
**Panel C: Retirement age**							
County-Year-Quarter and Cohort FE	0.506***	0.509***	0.452***	0.462***	0.427***	0.401***	0.554***
	(0.065)	(0.086)	(0.089)	(0.089)	(0.097)	(0.107)	(0.124)
Replace Cohort with Cohort-State FE	0.506***	0.509***	0.455***	0.465***	0.436***	0.415***	0.554***
	(0.065)	(0.086)	(0.089)	(0.090)	(0.097)	(0.108)	(0.124)
Replace Cohort with Cohort-County FE	0.506***	0.568***	0.521***	0.533***	0.506***	0.486***	0.619***
	(0.065)	(0.095)	(0.099)	(0.099)	(0.108)	(0.120)	(0.139)
**Panel D: SSDI (excluding unknowns)**							
County-Year-Quarter and Cohort FE	-0.016**	-0.022***	-0.020**	-0.021**	-0.017*	-0.021**	-0.031**
	(0.006)	(0.008)	(0.009)	(0.009)	(0.009)	(0.010)	(0.012)
Replace Cohort with Cohort-State FE	-0.016**	-0.021**	-0.020**	-0.021**	-0.017*	-0.021**	-0.031**
	(0.006)	(0.008)	(0.009)	(0.009)	(0.009)	(0.010)	(0.012)
Replace Cohort with Cohort-County FE	-0.016**	-0.023***	-0.021**	-0.023**	-0.020**	-0.024**	-0.033**
	(0.006)	(0.009)	(0.009)	(0.009)	(0.010)	(0.011)	(0.013)

Notes: Standard errors clustered at the level of fixed effects in parentheses, *** p<0.01, ** p<0.05, * p<0.1. Sample is restricted only to those that died after age >= 45 and have non-missing peer and camp characteristics. Column (1) includes only duration of service as regressor. Column (2) adds County-Year-Quarter of Enrollment fixed effects and the specified Cohort fixed effects (Birth Year, Birth Year-State, or Birth Year-County). Column (3) adds individual controls. Column (4) adds camp characteristics, such as distance from nearest city and average temperature. Column (5) adds peer characteristics, where peers are defined as other enrollees serving in the same camp at the same time. Column (6) adds camp fixed effects and removes camp characteristics. Column (7) runs the regression specification in Column (6) for only enrollees from our Colorado Records. For complete list of controls, refer to text or Appendix Table V.

Table A.VIII

Full Table for IV Estimates on Longevity and Lifetime Earnings

	(1)	(2)	(3)	(4)	(5)	(6)	(7)
Depdent variable	No Controls	Add Birth, County-qtr Dummies	Add Indiv Controls	Add Camp Chars	Add Peer Chars	Add Camp Fixed Effects	CO Only
**Panel A: Longevity for the full sample (log death age)**							
IV	0.019**	0.009	0.010	0.009	0.013	0.031	0.042
	(0.009)	(0.032)	(0.035)	(0.034)	(0.038)	(0.047)	(0.041)
OLS	0.012***	0.012***	0.011***	0.012***	0.014***	0.014***	0.013**
	(0.002)	(0.004)	(0.004)	(0.004)	(0.004)	(0.005)	(0.006)
FS	-0.449***	-0.193***	-0.181***	-0.186***	-0.165***	-0.142***	-0.211***
	(0.022)	(0.025)	(0.024)	(0.024)	(0.022)	(0.024)	(0.031)
F-stat	411.16	59.64	56.50	58.76	55.11	35.55	46.63
Observations	9,049	9,049	9,049	9,049	9,049	9,049	5,168
**Panel B: Average Indexed Monthly Earnings (MBR sample claimed 1979 and later)**							
IV	-175.634***	201.871	224.187	252.088*	287.937*	282.871	183.846
	(42.754)	(127.811)	(141.749)	(141.033)	(169.308)	(189.737)	(170.123)
OLS	-23.132*	59.259***	50.469***	49.597***	42.574**	41.000*	56.154**
	(13.245)	(17.306)	(18.390)	(18.675)	(20.402)	(21.920)	(27.096)
FS	-0.453***	-0.211***	-0.192***	-0.200***	-0.167***	-0.154***	-0.235***
	(0.023)	(0.028)	(0.026)	(0.027)	(0.024)	(0.026)	(0.036)
F-stat	381.03	58.27	54.01	56.75	47.42	34.28	41.76
Observations	5,529	5,529	5,529	5,529	5,529	5,529	3,143
**Panel C: Retirement age**							
IV	1.058***	0.571	0.582	0.767	1.189	1.040	3.035*
	(0.342)	(1.322)	(1.433)	(1.371)	(1.574)	(1.957)	(1.566)
OLS	0.467***	0.553***	0.505***	0.520***	0.511***	0.524***	0.663***
	(0.089)	(0.123)	(0.126)	(0.128)	(0.143)	(0.165)	(0.192)
FS	-0.455***	-0.193***	-0.181***	-0.188***	-0.162***	-0.139***	-0.221***
	(0.023)	(0.028)	(0.027)	(0.027)	(0.025)	(0.028)	(0.037)
F-stat	385.65	48.33	46.59	47.93	42.08	25.16	35.74
Observations	6,169	6,169	6,169	6,169	6,169	6,169	3,650
**Panel D: SSDI (excluding unknowns)**							
IV	0.007	0.021	0.029	0.009	0.018	0.045	-0.240**
	(0.027)	(0.092)	(0.104)	(0.098)	(0.114)	(0.135)	(0.116)
OLS	-0.015*	-0.030**	-0.031**	-0.032**	-0.023*	-0.028*	-0.038**
	(0.009)	(0.012)	(0.012)	(0.012)	(0.013)	(0.015)	(0.019)
FS	-0.453***	-0.210***	-0.191***	-0.199***	-0.170***	-0.154***	-0.234***
	(0.023)	(0.027)	(0.026)	(0.026)	(0.024)	(0.026)	(0.036)
F-stat	383.40	58.46	53.68	56.38	48.67	33.87	41.35
Observations	5,474	5,474	5,474	5,474	5,474	5,474	3,121

Notes: Standard errors clustered at the level of county-by-year-quarter of enlistment in parentheses, *** p<0.01, ** p<0.05, * p<0.1. Sample is restricted only to those that died after age >= 45, have non-missing peer and camp characteristics, and those who were dismissed after end of term or for the convenience of the government. Our instrumental variable (IV) is whether the enrollee was dismissed for convenience of the government. We present the 2-stage least squares (2SLS) intsrumental variable regression's coefficient on duration, OLS regression coefficient on duration, first stage coefficient on our instrument from regression of duration on the instrument, and F-statistic on the instrument from the first stage. Column (1) includes only duration of service as regressor. Column (2) adds Birth and County-Year-Quarter of Enrollment fixed effects. Column (3) adds individual controls. Column (4) adds camp characteristics, such as distance from nearest city and average temperature. Column (5) adds peer characteristics, where peers are defined as other enrollees serving in the same camp at the same time. Column (6) adds camp fixed effects and removes camp characteristics. Column (7) runs the regression specification in Column (6) for only enrollees from our Colorado Records. For complete list of controls, refer to text or Appendix Table V.

Table A.IX

Effect of Service Duration on Missing Data and Sample Selection

	(1)	(2)	(3)	(4)	(5)	(6)	(7)
VARIABLES	No Controls	Add Birth, County-qtr Dummies	Add Indiv Controls	Add Camp Chars	Add Peer Chars	Add Camp FE	CO Only
**Panel A: Does duration predict whether longevity is missing?**							
Duration of service (yrs)	0.001	-0.017***	-0.020***	-0.020***	-0.017***	-0.015***	-0.008
	(0.005)	(0.005)	(0.005)	(0.005)	(0.005)	(0.005)	(0.006)
Observations	22,964	22,964	22,964	22,964	22,964	22,964	14,116
R-squared	0.000	0.111	0.196	0.197	0.198	0.206	0.200
Mean Dep	0.18	0.18	0.18	0.18	0.18	0.18	0.15
**Panel B: Does duration predict being in the MBR sample?**							
Duration of service (yrs)	-0.006	0.004***	0.010*	0.011*	0.009	0.005	0.002
	(0.005)	(0.001)	(0.006)	(0.006)	(0.007)	(0.007)	(0.009)
Observations	22,980	22,980	22,980	22,980	22,980	22,980	14,116
R-squared	0.000	0.102	0.205	0.206	0.206	0.212	0.187
Mean Dep	0.53	0.53	0.53	0.53	0.53	0.53	0.57
**Panel C: Is the effect of duration on longevity for the MBR sample the same as in the full sample?**							
Duration of service (yrs)	0.013***	0.010***	0.009***	0.009***	0.012***	0.011***	0.014***
	(0.002)	(0.002)	(0.002)	(0.002)	(0.003)	(0.003)	(0.003)
Observations	11,953	11,953	11,953	11,953	11,953	11,953	7,913
R-squared	0.005	0.157	0.169	0.169	0.170	0.185	0.190
Mean Dep	74.81	74.81	74.81	74.81	74.81	74.81	74.78

Notes: Standard errors clustered at the level of county-by-year-quarter of enlistment in parentheses, *** p<0.01, ** p<0.05, * p<0.1. See Notes on Table III for specifications in each column. Panel A explores the outcome of = 1 if death age is missing, = 0 otherwise. Panel B explores the outcome of = 1 if in the MBR sample, = 0 otherwise. Panel C explores the outcome log death age (same as Table III Panel A), but only for the sample of individuals found in the MBR sample.

Table A.X: Effect of Service Duration on Survival Rates by Age - Imputing Missing Longevity

	(1)	(2)	(3)	(4)	(5)	(6)
**Panel A: Survival to age 70**	Mean Dep	0.65				
Duration of service (yrs)	0.030***	0.032***	0.028***	0.035***	0.030***	0.022***
	(0.005)	(0.006)	(0.006)	(0.007)	(0.008)	(0.008)
Observations	17,086					
**Panel B: Survival to age 70 missing imputed**	Mean Dep	0.64				
Duration of service (yrs)	0.022***	0.026***	0.023***	0.028***	0.023***	0.016**
	(0.004)	(0.005)	(0.005)	(0.006)	(0.006)	(0.007)
Observations	21,269					
**Panel C: Survival to age 70 missing imputed to 0**	Mean Dep	0.52				
Duration of service (yrs)	0.024***	0.037***	0.037***	0.040***	0.034***	0.020***
	(0.005)	(0.006)	(0.006)	(0.007)	(0.007)	(0.008)
Observations	21,269					
County-Quarter FE	N	Y	Y	Y	Y	Y
Controls	N	N	Y	Y	Y	Y
Peer + Camp Controls	N	N	N	Y	Y	Y
Camp FE	N	N	N	N	Y	Y
Type of Dismissal	N	N	N	N	N	Y

Notes: Standard errors clustered at the level of county-by-year-quarter of enlistment in parentheses, *** p<0.01, ** p<0.05, * p<0.1. Sample only includes death ages >= 45 and have non-missing peer and camp characteristics. Panel B imputes survival probability using the age at discharge, birth year, and life tables from SSA. Panel C imputes 0 for missing survival probability.

Table A.XI: The Effect of Service Duration for Machine-Matched Sample

VARIABLES	(1)<br>No Controls	(2)<br>Add Birth, County-qtr Dummies	(3)<br>Add Indiv Controls	(4)<br>Add Camp Chars	(5)<br>Add Peer Chars	(6)<br>Add Camp FE	(7)<br>CO Only
**Panel A: Longevity from CCC for the machined-matched sample**							
Duration of service (yrs)	0.013***	0.011***	0.010***	0.010***	0.012***	0.012***	0.019***
	(0.002)	(0.003)	(0.003)	(0.003)	(0.004)	(0.004)	(0.005)
Observations	8,833	8,833	8,833	8,833	8,833	8,833	5,904
R-squared	0.003	0.186	0.192	0.194	0.195	0.212	0.220
Mean Dep	72.64	72.64	72.64	72.64	72.64	72.64	72.41
**Panel B: Longevity from DMF for the matchine-matched sample**							
Duration of service (yrs)	0.013***	0.011***	0.010***	0.010***	0.012***	0.012***	0.019***
	(0.002)	(0.003)	(0.003)	(0.003)	(0.004)	(0.004)	(0.005)
Observations	9,175	9,175	9,175	9,175	9,175	9,175	6,071
R-squared	0.003	0.181	0.186	0.188	0.189	0.205	0.214
Mean Dep	72.65	72.65	72.65	72.65	72.65	72.65	72.42
**Panel C: Does duration predict whether they are machine-matched to DMF?**							
Duration of service (yrs)	0.015***	0.024***	0.026***	0.026***	0.024***	0.022***	0.020**
	(0.005)	(0.006)	(0.006)	(0.006)	(0.007)	(0.007)	(0.009)
Observations	22,964	22,964	22,964	22,964	22,964	22,964	14,116
R-squared	0.000	0.110	0.153	0.153	0.154	0.161	0.165
Mean Dep	0.41	0.41	0.41	0.41	0.41	0.41	0.44

Notes: Standard errors clustered at the level of county-by-year-quarter of enlistment in parentheses, *** p<0.01, ** p<0.05, * p<0.1. In Panel A, we use death age calculated from CCC birth year and death age from hand-matched sources. In Panel B we use death age calculated from DMF birth date and death date from the machine match. Sample is restricted only to those that died after age >= 45 and have non-missing peer and camp characteristics for Panels A and B.

Table A.XII: Effect of Service Duration on SSA Outcomes

VARIABLES	(1)<br>No Controls	(2)<br>Add Birth, County-qtr Dummies	(3)<br>Add Indiv Controls	(4)<br>Add Camp Chars	(5)<br>Add Peer Chars	(6)<br>Add Camp FE	(7)<br>CO Only
**Panel A: What is the effect of duration on PIA in the MBR sample? (Claimed 1979 and later)**							
Duration of service (yrs)	-1.675	21.706***	19.893***	19.717***	18.979***	17.083***	15.459***
	(2.869)	(3.743)	(3.827)	(3.841)	(4.284)	(4.636)	(5.414)
Observations	10,241	10,241	10,241	10,241	10,241	10,241	6,525
R-squared	0.000	0.200	0.215	0.216	0.218	0.233	0.254
Mean Dep	437.70	437.70	437.70	437.70	437.70	437.70	449.34
Mean Implied AIME	904.62	904.62	904.62	904.62	904.62	904.62	940.99
Implied AIME Increase	-5.23	67.83	62.17	61.62	59.31	53.38	48.31
**Panel B: What is the effect of duration on PIA in the MBR sample? (Claimed earlier than 1979)**							
Duration of service (yrs)	13.075***	12.552**	12.692**	10.713*	8.819	8.792	8.088
	(3.857)	(6.107)	(6.313)	(6.481)	(7.394)	(10.585)	(11.020)
Observations	1,562	1,562	1,562	1,562	1,562	1,562	1,284
R-squared	0.007	0.456	0.503	0.507	0.511	0.557	0.526
Mean Dep	314.02	314.02	314.02	314.02	314.02	314.02	317.41
**Panel C: What is the effect of duration on SSDI claiming in the MBR sample? (excluding unknowns)**							
Duration of service (yrs)	-0.016**	-0.022***	-0.020**	-0.021**	-0.017*	-0.021**	-0.031***
	(0.006)	(0.008)	(0.009)	(0.009)	(0.010)	(0.010)	(0.012)
Observations	10145	10145	10145	10145	10145	10145	6480
R-squared	0.001	0.154	0.161	0.163	0.164	0.181	0.205
Mean Dep	0.21	0.21	0.21	0.21	0.21	0.21	0.20
**Panel D: What is the effect of duration on SSDI claiming in the MBR sample? (unknowns grouped with those who claimed)**							
Duration of service (yrs)	-0.019***	-0.022***	-0.022**	-0.023***	-0.020**	-0.022**	-0.030**
	(0.006)	(0.008)	(0.009)	(0.009)	(0.010)	(0.011)	(0.012)
Observations	10373	10373	10373	10373	10373	10373	6613
R-squared	0.001	0.154	0.161	0.163	0.164	0.179	0.201
Mean Dep	0.22	0.22	0.22	0.22	0.22	0.22	0.22
**Panel E: What is the effect of duration on SSDI claiming in the MBR sample? (unknowns grouped with those who did NOT claim)**							
Duration of service (yrs)	-0.015**	-0.020**	-0.018**	-0.019**	-0.014	-0.019*	-0.030**
	(0.006)	(0.008)	(0.008)	(0.008)	(0.009)	(0.010)	(0.012)
Observations	10373	10373	10373	10373	10373	10373	6613
R-squared	0.001	0.151	0.157	0.160	0.161	0.178	0.202
Mean Dep	0.20	0.20	0.20	0.20	0.20	0.20	0.20

Notes: Standard errors clustered at the level of county-by-year-quarter of enlistment in parentheses, *** p<0.01, ** p<0.05, * p<0.1. Sample is restricted only to those that died after age >= 45 and have non-missing peer and camp characteristics. Column (1) includes only duration of service as regressor. Column (2) adds Birth and County-Year-Quarter of Enrollment fixed effects. Column (3) adds individual controls. Column (4) adds camp characteristics, such as distance from nearest city and average temperature. Column (5) adds peer characteristics, where peers are defined as other enrollees serving in the same camp at the same time. Column (6) adds camp fixed effects and removes camp characteristics. Column (7) runs the regression specification in Column (6) for only enrollees from our Colorado Records. For complete list of controls, refer to text or Appendix Table V.

Appendix Table XIII: Heterogeneity in OLS effects

	(1)	(2)	(3)	(4)	(5)	(6)	(7)	(8)	(9)
Sample	CO	NM	Age <= 18	Age > 18	Allottee Mother	Allottee Father	Allottee Other	Urate above median	Urate below median
**Panel A: Log Death Age**									
Duration of service (yrs)	0.013***	0.014**	0.014***	0.013***	0.018***	0.009	0.011	0.017***	0.013
	(0.003)	(0.005)	(0.005)	(0.004)	(0.004)	(0.006)	(0.009)	(0.004)	(0.009)
Observations	11,148	6,243	8,042	9,349	8,253	5,801	3,337	8,238	2,742
Mean Death Age	73.29	74.18	72.95	74.18	73.36	74.23	73.14	73.65	73.54
**Panel B: AIME**									
Duration of service (yrs)	52.954***	46.739*	91.362***	9.960	35.534	49.074*	84.455	53.264**	124.672***
	(17.205)	(28.001)	(22.828)	(21.695)	(22.280)	(27.363)	(53.510)	(21.215)	(47.604)
Observations	6,734	3,779	5,660	4,853	5,126	3,562	1,825	5,316	1,674
Mean AIME	1012.37	881.01	1028.30	891.50	980.51	948.40	954.73	964.99	961.95
**Panel C: Retirement or SSDI claiming age**									
Duration of service (yrs)	0.549***	0.007	0.520***	0.336**	0.463***	0.427**	0.306	0.440**	0.458
	(0.119)	(0.229)	(0.187)	(0.138)	(0.165)	(0.212)	(0.298)	(0.180)	(0.338)
Observations	8,006	4,006	5,602	6,410	5,717	3,970	2,325	5,723	1,891
Mean Age	60.45	59.95	59.93	60.59	60.19	60.38	60.33	60.28	60.25
**Panel D: SSDI (excluding unknowns)**									
Duration of service (yrs)	-0.033**	-0.012	-0.057***	-0.019	-0.049***	-0.005	-0.010	-0.039*	-0.063*
	(0.014)	(0.025)	(0.020)	(0.016)	(0.018)	(0.022)	(0.034)	(0.021)	(0.033)
Observations	8,160	4,104	5,745	6,519	5,835	4,058	2,371	5,845	1,919
Mean SSDI	0.25	0.28	0.28	0.25	0.26	0.26	0.26	0.26	0.24

	(10)	(11)	(12)	(13)	(14)	(15)	(16)	(17)	(18)
Sample	Hispanic	Not Hispanic	BMI < 18.5 (CO)	BMI 18.5-25 (CO)	BMI >= 25 (CO)	Phase 2 (1935-1937)	Phase 3 (1937-1940)	Phase 4 (1940-1942)	Random-ized
**Panel A: Log Death Age**									
Duration of service (yrs)	0.018***	0.009**	0.008	0.013**	0.098	0.022***	0.022***	0.015	0.020***
	(0.005)	(0.004)	(0.071)	(0.007)	(0.137)	(0.006)	(0.005)	(0.009)	(0.005)
Observations	7,864	9,527	433	5,627	290	3,852	7,256	6,049	5,170
Mean Death Age	74.29	73.05	72.27	73.20	71.50	73.72	73.69	73.46	73.44
**Panel B: AIME**									
Duration of service (yrs)	51.333**	65.612***		74.345**		21.357	67.962***	116.843**	59.236**
	(23.224)	(21.241)		(30.643)		(34.184)	(25.374)	(45.510)	(26.387)
Observations	4,758	5,755		4,023		1,739	4,633	4,106	3,123
Mean AIME	878.44	1036.85		1043.45		941.61	958.81	982.84	950.43
**Panel C: Retirement or SSDI claiming age**									
Duration of service (yrs)	0.389**	0.519***		0.840***		0.523***	0.572***	0.457	0.477**
	(0.184)	(0.136)		(0.243)		(0.195)	(0.200)	(0.397)	(0.189)
Observations	5,171	6,841		4,115		2,692	5,067	4,101	3,538
Mean Age	60.22	60.33		60.24		60.88	60.30	59.83	60.17
**Panel D: SSDI (excluding unknowns)**									
Duration of service (yrs)	-0.033	-0.028*		-0.061*		-0.021	-0.050**	-0.002	-0.006
	(0.021)	(0.016)		(0.031)		(0.027)	(0.024)	(0.044)	(0.025)
Observations	5,318	6,946		4,215		2,723	5,157	4,231	3,626
Mean SSDI	0.29	0.24		0.26		0.23	0.25	0.30	0.27

Notes: Standard errors clustered at the level of county-by-year-quarter of enlistment in parentheses, *** p<0.01, ** p<0.05, * p<0.1. Sample is restricted only to those that died after age >= 45, have non-missing peer and camp characteristics and restrictions described by column headings. The specification uses the most restrictive specification with Camp FE, which was the specification used in Table III, Column 6.

Appendix Table XIV: Effect of Service Duration on Labor Market Outcomes Observed in the 1940 Census

	(1)	(2)	(3)	(4)	(5)	(6)	(7)
Regression of Outcome on Duration	No Controls	Add Birth, County-qtr Dummies	Add Indiv Controls	Add Camp Chars	Add Peer Chars	Add Camp FE	CO Only
Census							
**Found in Census Records**	Mean Dep	0.43					
Duration of service (yrs)	-0.015**	0.009	0.007	0.009	0.006	0.012	0.011
	(0.007)	(0.010)	(0.010)	(0.010)	(0.011)	(0.012)	(0.013)
Observations	9,518	9,518	9,518	9,518	9,518	9,518	7,553
R-squared	0.001	0.137	0.152	0.154	0.155	0.166	0.154
**In Labor Force**	Mean Dep	0.91					
Duration of service (yrs)	0.014**	0.013*	0.013*	0.015**	0.016*	0.018*	0.018*
	(0.006)	(0.007)	(0.007)	(0.007)	(0.009)	(0.010)	(0.011)
Observations	4,052	4,052	4,052	4,052	4,052	4,052	3,374
R-squared	0.001	0.272	0.279	0.280	0.280	0.305	0.286
**Working in Census Week | Labor Force**	Mean Dep	0.71					
Duration of service (yrs)	0.006	-0.004	-0.005	-0.004	-0.010	-0.016	-0.012
	(0.011)	(0.014)	(0.014)	(0.014)	(0.019)	(0.022)	(0.023)
Observations	3,684	3,684	3,684	3,684	3,684	3,684	3,067
R-squared	0.000	0.265	0.279	0.283	0.286	0.310	0.295
**Weeks Worked in 1939^**	Mean Dep	27.88					
Duration of service (yrs)	0.705	-0.663	-0.861	-0.892	-0.858	0.316	0.285
	(0.743)	(1.049)	(1.051)	(1.029)	(1.082)	(1.194)	(1.209)
Observations	2,361	2,361	2,361	2,361	2,361	2,361	2,209
R-squared	0.000	0.314	0.345	0.351	0.354	0.383	0.361
**Total Annual Wage in 1939^**	Mean Dep	383.71					
Duration of service (yrs)	16.486	-13.381	-19.703	-20.758	-21.943	-14.977	-15.095
	(15.960)	(23.212)	(23.926)	(23.513)	(25.559)	(26.394)	(26.650)
Observations	2,149	2,149	2,149	2,149	2,149	2,149	2,012
R-squared	0.001	0.317	0.352	0.356	0.358	0.391	0.375
**Ln Total Annual Wage | Working^**	Mean Dep	471.25					
Duration of service (yrs)	0.046	-0.038	-0.047	-0.042	-0.051	-0.015	-0.013
	(0.039)	(0.052)	(0.052)	(0.052)	(0.058)	(0.062)	(0.062)
Observations	1,750	1,750	1,750	1,750	1,750	1,750	1,650
R-squared	0.001	0.396	0.447	0.452	0.454	0.487	0.456

Notes: Standard errors clustered at the level of county-by-year-quarter of enlistment in parentheses, *** p<0.01, ** p<0.05, * p<0.1. Sample are those whose first term in CCC is before 1940 and are not enrolled in 1940. The 1940 Census was taken on April 1, 1940. ^ Sample are those whose first term in CCC is before 1939 and are not enrolled in 1939. Census asks labor force and work status on the week before the Census enumeration, while wage information and weeks worked is asked for the year before the Census 1939.

Appendix Table XV: Effect of Service Duration on WWII Service, Health and Education Observed in WWII Enlistment and 1940 Census

	(1)	(2)	(3)	(4)	(5)	(6)	(7)
Regression of Outcome on Duration	No Controls	Add Birth, County-qtr Dummies	Add Indiv Controls	Add Camp Chars	Add Peer Chars	Add Camp FE	CO Only
WWII							
**Found in WWII Records**	Mean Dep	0.31					
Duration of service (yrs)	0.018***	0.036***	0.035***	0.035***	0.038***	0.038***	0.042***
	(0.005)	(0.006)	(0.006)	(0.006)	(0.007)	(0.007)	(0.009)
Observations	22,964	22,964	22,964	22,964	22,964	22,964	14,116
**Enlistment Year**	Mean Dep	1942.24					
Duration of service (yrs)	-0.181***	0.976***	0.975***	0.976***	0.966***	0.962***	0.964***
	(0.025)	(0.008)	(0.008)	(0.008)	(0.009)	(0.010)	(0.011)
Observations	7,018	7,018	7,018	7,018	7,018	7,018	4,785
**Height**	Mean Dep	67.55					
Duration of service (yrs)	-0.022	1.098***	1.098***	1.097***	1.162***	1.143***	1.208***
	(0.103)	(0.190)	(0.191)	(0.190)	(0.209)	(0.221)	(0.276)
Observations	5,770	5,770	5,770	5,770	5,770	5,770	3,816
**Height, for Age 20 or Older**							
Duration of service (yrs)	-0.066	0.509	0.567*	0.610*	0.620	0.822	1.320*
	(0.196)	(0.357)	(0.343)	(0.341)	(0.388)	(0.504)	(0.683)
Observations	1,414	1,414	1,414	1,414	1,414	1,414	857
**Height, for Age < 20**							
Duration of service (yrs)	-0.004	1.238***	1.248***	1.238***	1.356***	1.478***	1.529***
	(0.118)	(0.248)	(0.251)	(0.249)	(0.273)	(0.284)	(0.347)
Observations	4,356	4,356	4,356	4,356	4,356	4,356	2,959
**BMI**	Mean Dep	21.53					
Duration of service (yrs)	-0.134**	0.789***	0.829***	0.823***	0.874***	1.017***	1.155***
	(0.064)	(0.191)	(0.191)	(0.190)	(0.195)	(0.204)	(0.264)
Observations	5,287	5,287	5,287	5,287	5,287	5,287	3,454
Combined WWII Census							
**Education**	Mean Dep	9.23					
Duration of service (yrs)	-0.072**	0.299***	0.185***	0.186***	0.188***	0.169***	0.115***
	(0.035)	(0.041)	(0.036)	(0.036)	(0.038)	(0.040)	(0.043)
Observations	9,586	9,586	9,586	9,586	9,586	9,586	6,907

Notes: Standard errors clustered at the level of county-by-year-quarter of enlistment in parentheses,, *** p<0.01, ** p<0.05, * p<0.1. Sample are those found in WWII records. WWII: additionally includes the age at enlistment dummies. Combined: additionally includes age at observation dummies, where if observed in Census, the age is 1940 - birth year.

Appendix Table XVI: Effect of Service Duration on Geographic Mobility Over the Lifetime

	(1)	(2)	(3)	(4)	(5)	(6)	(7)
Regression of Outcome on Duration	No Controls	Add Birth, County-qtr Dummies	Add Indiv Controls	Add Camp Chars	Add Peer Chars	Add Camp FE	CO Only
Panel A: Short-term geographic mobility (Combined WW2 and Census)							
**Moved to a Different State**	Mean Dep	0.09					
Duration of service (yrs)	-0.014***	0.023***	0.024***	0.023***	0.028***	0.026***	0.033***
	(0.004)	(0.006)	(0.006)	(0.006)	(0.007)	(0.007)	(0.009)
Observations	9,568	9,568	9,568	9,568	9,568	9,568	6,891
**Moved to a Different County**	Mean Dep	0.33					
Duration of service (yrs)	0.006	0.053***	0.054***	0.053***	0.062***	0.057***	0.067***
	(0.007)	(0.009)	(0.009)	(0.009)	(0.011)	(0.011)	(0.012)
Observations	9,568	9,568	9,568	9,568	9,568	9,568	6,891
**New County Has Higher Yearly Wage Than Sending County**				Mean Dep	0.65		
Duration of service (yrs)	-0.003	0.035**	0.031*	0.030*	0.028	0.045**	0.037*
	(0.013)	(0.016)	(0.016)	(0.016)	(0.017)	(0.020)	(0.021)
Observations	3,165	3,165	3,165	3,165	3,165	3,165	2,565
**New County Has Above Median Mortality Rate (1950-1968)**				Mean Dep	0.38		
Duration of service (yrs)	-0.056***	-0.047***	-0.049***	-0.051***	-0.049**	-0.043*	-0.040
	(0.011)	(0.017)	(0.018)	(0.018)	(0.019)	(0.023)	(0.025)
Observations	3,175	3,175	3,175	3,175	3,175	3,175	2,565
Panel B: Long-term geographic mobility							
**Died in a Different State**	Mean Dep	0.5					
Duration of service (yrs)	-0.016*	-0.020*	-0.025**	-0.026**	-0.027**	-0.029**	-0.027*
	(0.008)	(0.012)	(0.012)	(0.012)	(0.013)	(0.015)	(0.015)
Observations	7,235	7,235	7,235	7,235	7,235	7,235	4,784
**Died in a Different County**	Mean Dep	0.79					
Duration of service (yrs)	0.003	-0.002	-0.005	-0.004	0.002	0.003	0.001
	(0.007)	(0.010)	(0.010)	(0.010)	(0.011)	(0.012)	(0.012)
Observations	7,079	7,079	7,079	7,079	7,079	7,079	4,677
**New County Has Above Median Mortality Rate (1950-1968)**				Mean Dep	0.25		
Duration of service (yrs)	-0.030***	0.004	0.006	0.005	0.003	0.006	0.009
	(0.008)	(0.012)	(0.012)	(0.012)	(0.013)	(0.015)	(0.016)
Observations	5,313	5,313	5,313	5,313	5,313	5,313	3,678

Notes: We assume that the person lived in the county of application when definining wheter a person moved. Standard errors clustered at the level of county-by-year-quarter of enlistment in parentheses, *** p<0.01, ** p<0.05, * p<0.1. Sample are those found in WWII records. WWII: additionally includes the age at enlistment dummies. Combined: additionally includes age at observation dummies, where if observed in Census, the age is 1940 - birth year.

Appendix Table XVII: Placebo Tests for CO Only

	(1)	(2)	(3)	(4)	(5)	(6)
Regression of Outcome on Duration	No Controls	Add Birth, County-qtr Dummies	Add Indiv Controls	Add Camp Chars	Add Peer Chars	Add Camp FE
**Education**	Mean Dep	8.72				
Duration of service (yrs)	0.192***	0.230***	0.234***	0.238***	0.216***	0.181***
	(0.041)	(0.048)	(0.047)	(0.047)	(0.056)	(0.056)
N	6,823	6,823	6,823	6,823	6,823	6,823
**Height**	Mean Dep	67.94				
Duration of service (yrs)	-0.029	-0.213	-0.065	-0.055	-0.158	-0.200
	(0.125)	(0.171)	(0.146)	(0.150)	(0.179)	(0.187)
N	2,319	2,319	2,319	2,319	2,319	2,319
**Weight (100 pounds)**	Mean Dep	1.40				
Duration of service (yrs)	-0.012*	-0.016	-0.008	-0.008	-0.005	-0.002
	(0.007)	(0.011)	(0.008)	(0.008)	(0.010)	(0.010)
N	2,067	2,067	2,067	2,067	2,067	2,067
**Ever Had a Paid Job**	Mean Dep	0.45				
Duration	-0.007	-0.018	-0.048	-0.061	-0.065	-0.048
	(0.032)	(0.051)	(0.048)	(0.047)	(0.049)	(0.059)
Observations	1,104	1,104	1,104	1,104	1,104	1,104

Notes: Standard errors clustered at the level of county-by-year-quarter of enlistment in parentheses, *** p<0.01, ** p<0.05, * p<0.1. Dependent variables are pre-program characteristics of individuals. Each column's specification corresponds to column specifications in Table III. Regressions do not include imputed values.

Appendix Table XVIII

Characteristics of Eligible Job Corps Applicants and Comparison to CCC

Characteristic	Job Corps Data		CCC
	All Applicants	Males only	Males Only
**Baseline Characteristics**			
Duration for treated (years)	0.483	0.487	0.819
Duration (in years, only positive durations)	0.67	0.652	0.819
Male	0.6	1	1
Age at application	18.8	18.728	18.75
White, non-Hispanic	0.3	0.304	NA
Black, non-Hispanic	0.5	0.451	NA
Hispanic	0.2	0.169	0.484
Other	0.1	0.076	NA
Years of education	10.2	10.042	8.581
High school diploma or more (including GED)	0.2	0.19	0.12
Ever arrested	0.3	0.332	NA
Had a job in the past year	0.6	0.662	NA
Ever had job	0.8	0.808	0.375
Average earnings in the past year (dollars)	2974.9	3255.739	NA
**Mean for outcomes**			
Years of school	11.145	11.07	9.403
Employment (in week of the survey)^	0.606	0.631	0.71
Weeks worked in previous year	30.62	32.17	27.88
Total ann. earnings in prev. yr	10538.31	11947.78	382.43
Total ann. earnings in prev. yr (weeks worked > 0)	12990.85	14471.77	466.69
Moved^^	0.198	0.207	0.34
Self-reported health status in 12 months^^^	1.786	1.733	NA
Self-reported health status in 48 months^^^	1.809	1.757	NA
Self-reported health excellent or good (12-month)*	0.838	0.855	NA
Self-reported health excellent or good (48-month)*	0.828	0.842	NA
Reason ended: End of term	0.31	0.302	0.378
Reason ended: Employment	0.042	0.038	0.116
Reason ended: Convenience of the government	0.001	0	0.145
Reason ended: Urgent and Proper Call	0.09	0.056	0.116
Reason ended: Deserted	0.331	0.373	0.223
Reason ended: Rejected upon examination	0	0	0.0101
Reason ended: No Record	0.228	0.232	0.0127
Observations: Baseline	14327	8646	NA
Observations: Outcomes	11313	6528	NA

Source: Jobs Corps Baseline data. ^employment is not conditional on labor force participation. ^^for Job Corps it is defined as living more than 20 miles away from baseline residence. For CCC it is defined as living in a different county than the county of residence at the time of enrollment. For Job Corps, employment is defined as having a job during the 208th week after the baseline survey (four years). ^^^Self-reported health status with 1 = excellent health, 2 = good, 3 = fair, and 4 = poor health. *Constructed variable that is equal to 1 if self-reported health status is 1 or 2 (excellent health or good health).

Appendix Table XIX

Relative Characteristics of Eligible Job Corps Applicants and Comparison to CCC

	Job Corps Data		CCC
Characteristic	Males Only	Reweighted	Males Only
**Relative Characteristics**			
School Grade	-0.814	-0.480	-0.481
Hispanic Imputed	0.970	1.135	1.135
Unemployed	2.005	1.829	1.831
Farm	0.118	-0.115	-0.119
Household Size	0.592	0.007	0.007

Source: Jobs Corps Baseline data. Reweighted means use weights generated from entropy balance method by Hainmueller (2012). Relative characteristics are generated by standardizing each variable by the mean and standard deviation in the 1940 Census (only Colorado and New Mexico) for the CCC sample and 1990 Census for the JC Sample by using males ages 16 to 24.

Appendix Table XX: Balance Test of Baseline Characteristics for Job Corps Applicants

Characteristic	Full sample				Males only			
	Treatment	Control	Difference		Treatment	Control	Difference	
Male	0.591	0.599	-0.008	(0.009)				
Age	18.861	18.826	0.035	(0.038)	18.735	18.717	0.018	(0.047)
White - Non-Hispanic	0.274	0.265	0.009	(0.008)	0.309	0.295	0.014	(0.01)
Black - Non-Hispanic	0.476	0.478	-0.002	(0.009)	0.45	0.452	-0.002	(0.011)
Hispanic	0.174	0.181	-0.007	(0.007)	0.163	0.178	-0.015*	(0.008)
Non-English Native Language	0.141	0.143	-0.001	(0.006)	0.14	0.144	-0.004	(0.008)
Has Child	0.181	0.179	0.002	(0.007)	0.106	0.108	-0.002	(0.007)
Childhood Household Head - Mother	0.483	0.49	-0.007	(0.009)	0.45	0.467	-0.016	(0.011)
Highest Grade Completed - Mother	11.516	11.539	-0.022	(0.051)	11.678	11.658	0.02	(0.062)
Highest Grade Completed - Father	11.471	11.578	-0.107	(0.064)	11.605	11.608	-0.003	(0.079)
Never on Welfare During Childhood	0.47	0.459	0.012	(0.009)	0.489	0.485	0.004	(0.012)
Highest Grade Completed	10.069	10.081	-0.012	(0.027)	9.953	9.969	-0.016	(0.032)
High School Degree	0.178	0.182	-0.004	(0.007)	0.139	0.142	-0.003	(0.008)
GED	0.047	0.055	-0.008*	(0.004)	0.05	0.052	-0.001	(0.005)
Ever Worked	0.8	0.788	0.011	(0.007)	0.812	0.801	0.011	(0.009)
Worked in Past Year	0.649	0.64	0.009	(0.008)	0.666	0.655	0.012	(0.01)
Currently has Job	0.215	0.208	0.007	(0.007)	0.221	0.204	0.017*	(0.009)
Months Worked in Past Year	6.055	6.127	-0.072	(0.092)	6.028	6.067	-0.039	(0.113)
Earnings in Past Year (if employed during past year)	3019.38	2903.82	115.556	(103.731)	3319.1	3156.06	163.035	(137.756)
Typical Hours Worked (if employed during past year)	35.635	35.344	0.291	(0.348)	36.922	36.73	0.192	(0.44)
Typical Wage (if employed during past year)	5.062	5.078	-0.017	(0.033)	5.167	5.194	-0.027	(0.042)
Received AFDC	0.316	0.316	-0.001	(0.009)	0.244	0.242	0.002	(0.01)
Received Food Stamps	0.437	0.446	-0.009	(0.009)	0.37	0.378	-0.008	(0.011)
Received Any Welfare	0.578	0.585	-0.007	(0.009)	0.511	0.518	-0.007	(0.012)
Ever Used Drugs	0.386	0.376	0.01	(0.009)	0.43	0.423	0.007	(0.011)
Ever Arrested	0.264	0.266	-0.001	(0.008)	0.337	0.326	0.011	(0.01)
Non-residential Job Corps Participant	0.137	0.141	-0.004	(0.006)	0.067	0.072	-0.005	(0.005)
Obs	8813	5514	14327		5036	3610	8646	

Notes: Robust standard errors in parentheses, *** p<0.01, ** p<0.05, * p<0.1. Data source is baseline data for Job Corps program from Schochet, Burghardt, and McConnell (2008). If employed during past year is measured as the individual worked for at least 2 weeks in the previous year.

Appendix Table XXI: Control Function Approach 1 (Assuming Treatment Effect is Same)

	(1)	(2)	(3)	(4)	(5)	(6)	(7)	(8)
		Log Death Age		*AIME*		*Retirement Age*		*SSDI*
Dependent Variable:	*Log Death Age*	*(Reweighted)*	*AIME*	*(Reweighted)*	*Retirement Age*	*(Reweighted)*	*SSDI*	*(Reweighted)*
**Panel A: Using Education Only**								
OLS	0.013***	0.013***	47.882**	47.882**	0.394***	0.394***	-0.020	-0.020
	(0.004)	(0.004)	(21.416)	(21.416)	(0.141)	(0.141)	(0.014)	(0.014)
Ctrl Common	0.013***	0.013***	52.363**	50.230**	0.418***	0.407***	-0.022	-0.021
	(0.004)	(0.004)	(21.640)	(21.512)	(0.142)	(0.141)	(0.014)	(0.014)
Ctrl All	0.013***	0.013***	52.109**	50.136**	0.417***	0.406***	-0.022	-0.021
	(0.004)	(0.004)	(21.625)	(21.510)	(0.142)	(0.141)	(0.014)	(0.014)
N	7,722	7,722	4,613	4,613	5,446	5,446	4,575	4,575
**Panel B: Using Moved Only**								
OLS	0.013***	0.013***	48.286**	48.286**	0.391***	0.391***	-0.018	-0.018
	(0.004)	(0.004)	(21.354)	(21.354)	(0.142)	(0.142)	(0.014)	(0.014)
Ctrl Common	0.013***	0.013***	48.421**	48.783**	0.391***	0.393***	-0.018	-0.018
	(0.004)	(0.004)	(21.364)	(21.431)	(0.142)	(0.143)	(0.014)	(0.014)
Ctrl All	0.013***	0.013***	48.303**	48.720**	0.391***	0.392***	-0.018	-0.018
	(0.004)	(0.004)	(21.353)	(21.410)	(0.142)	(0.143)	(0.014)	(0.014)
N	7,703	7,703	4,600	4,600	5,432	5,432	4,562	4,562
**Panel C: Using Others Only**								
OLS	0.024*	0.024*	-17.604	-17.604	0.589*	0.589*	-0.112**	-0.112**
	(0.012)	(0.012)	(75.655)	(75.655)	(0.329)	(0.329)	(0.051)	(0.051)
Ctrl Common	0.025**	0.025*	-8.394	-5.364	0.666**	0.714**	-0.112**	-0.113**
	(0.012)	(0.013)	(77.832)	(77.643)	(0.329)	(0.331)	(0.052)	(0.052)
Ctrl All	0.025**	0.025*	-8.041	-5.940	0.664**	0.715**	-0.111**	-0.113**
	(0.012)	(0.013)	(77.952)	(77.703)	(0.328)	(0.332)	(0.052)	(0.052)
N	1,382	1,382	621	621	1,010	1,010	621	621
**Panel D: All Control Functions**								
OLS	0.025**	0.025**	-18.237	-18.237	0.606*	0.606*	-0.112**	-0.112**
	(0.012)	(0.012)	(76.171)	(76.171)	(0.327)	(0.327)	(0.050)	(0.050)
Ctrl Common	0.025**	0.024**	-18.255	-9.318	0.655**	0.715**	-0.109**	-0.110**
	(0.012)	(0.012)	(77.637)	(77.396)	(0.333)	(0.337)	(0.053)	(0.054)
Ctrl All	0.025**	0.024**	-17.625	-9.388	0.661**	0.720**	-0.108**	-0.110**
	(0.012)	(0.012)	(77.739)	(77.388)	(0.333)	(0.338)	(0.053)	(0.053)
N	1,362	1,362	611	611	995	995	611	611

Notes: Standard errors clustered at the level of county-by-year-quarter of enlistment in parentheses, *** p<0.01, ** p<0.05, * p<0.1. These specifications use control functions calculated under the assumption that treatment effect between CCC and JC are the same. Panel A-Panel D includes different sets of control functions for the unweighted and the reweighted sample, where the weights are calculated using Hainmueller (2012) with relative disadvantages as inputs. In Panel A, we only include control function generated using education as the short-run outcome. In Panel B, the short-run outcome is short-run mobility from the 1940 Census and WWII rolls. Panel C uses whether working, weeks worked, log wage as short-run outcomes. Panel D includes all control functions in Panel A to Panel C simultaenously. Three results for each of these samples are presented. OLS row presents the OLS estimate without including the control functions on the sample of observations where each control function can be calculated. Ctrl Common row presents the results with control functions using only common covariates between JC and CCC (enrollment age, age less than 18 indicator, highest grade level, hispanic status, whether helpd a previous job, whether graduated high school, household size, from rural hosehold, whether father is living, whether mother is living). Ctrl All row presents the results with control functions using common covariates as well as other variables included in the full specifications corresponding to Table III Column 6.

Appendix Table XXII: Control Function Approach 2 (Assuming Selection Bias is Same)

	(1)	(2)	(3)	(4)	(5)	(6)	(7)	(8)
Dependent Variable:	*Log Death Age*	*Log Death Age (Reweighted)*	*AIME*	*AIME (Reweighted)*	*Retirement Age*	*Retirement Age (Reweighted)*	*SSDI*	*SSDI (Reweighted)*
**Panel A: Using Education Only**								
OLS	0.013***	0.013***	47.882**	47.882**	0.394***	0.394***	-0.020	-0.020
	(0.004)	(0.004)	(21.416)	(21.416)	(0.141)	(0.141)	(0.014)	(0.014)
Ctrl Common	0.013***	0.013***	46.809**	45.816**	0.388***	0.382***	-0.019	-0.019
	(0.004)	(0.004)	(21.391)	(21.377)	(0.142)	(0.142)	(0.014)	(0.014)
Ctrl All	0.013***	0.013***	48.295**	47.856**	0.396***	0.394***	-0.020	-0.020
	(0.004)	(0.004)	(21.434)	(21.419)	(0.141)	(0.141)	(0.014)	(0.014)
N	7,722	7,722	4,613	4,613	5,446	5,446	4,575	4,575
**Panel B: Using Moved Only**								
OLS	0.013***	0.013***	48.286**	48.286**	0.391***	0.391***	-0.018	-0.018
	(0.004)	(0.004)	(21.354)	(21.354)	(0.142)	(0.142)	(0.014)	(0.014)
Ctrl Common	0.013***	0.013***	48.517**	48.535**	0.392***	0.392***	-0.018	-0.018
	(0.004)	(0.004)	(21.382)	(21.381)	(0.142)	(0.142)	(0.014)	(0.014)
Ctrl All	0.013***	0.013***	48.423**	48.519**	0.395***	0.397***	-0.018	-0.018
	(0.004)	(0.004)	(21.404)	(21.441)	(0.142)	(0.143)	(0.014)	(0.014)
N	7,703	7,703	4,600	4,600	5,432	5,432	4,562	4,562
**Panel C: Using Others Only**								
OLS	0.024*	0.024*	-17.604	-17.604	0.589*	0.589*	-0.112**	-0.112**
	(0.012)	(0.012)	(75.655)	(75.655)	(0.329)	(0.329)	(0.051)	(0.051)
Ctrl Common	0.024*	0.023*	-9.744	-6.240	0.635**	0.666**	-0.107**	-0.105**
	(0.012)	(0.013)	(77.793)	(78.023)	(0.322)	(0.325)	(0.052)	(0.052)
Ctrl All	0.023*	0.022*	-15.035	-10.350	0.616*	0.655**	-0.108**	-0.108**
	(0.012)	(0.013)	(77.093)	(77.224)	(0.324)	(0.324)	(0.051)	(0.051)
N	1,382	1,382	621	621	1,010	1,010	621	621
**Panel D: All Control Functions**								
OLS	0.025**	0.025**	-18.237	-18.237	0.606*	0.606*	-0.112**	-0.112**
	(0.012)	(0.012)	(76.171)	(76.171)	(0.327)	(0.327)	(0.050)	(0.050)
Ctrl Common	0.025**	0.024*	-0.582	4.096	0.724**	0.767**	-0.106**	-0.104*
	(0.012)	(0.013)	(77.298)	(77.908)	(0.334)	(0.341)	(0.052)	(0.053)
Ctrl All	0.024*	0.023*	-15.336	-11.048	0.683**	0.722**	-0.107**	-0.106**
	(0.012)	(0.012)	(77.140)	(77.440)	(0.330)	(0.332)	(0.052)	(0.052)
N	1,362	1,362	611	611	995	995	611	611

Notes: Standard errors clustered at the level of county-by-year-quarter of enlistment in parentheses, *** p<0.01, ** p<0.05, * p<0.1. These specifications use control functions calculated under the assumption that treatment effect between CCC and JC are different but selection bias is the same. Panel A-Panel B uses different includes different sets of control functions for the unweighted and the reweighted sample, where the weights are calculated using Hainmueller (2012) with relative disadvantages as inputs. In Panel A, we only include control function generated using education as the short-run outcome. In Panel B, the short-run outcome is short-run mobility from the 1940 Census and WWII rolls. Panel C uses whether working, weeks worked, log wage as short-run outcomes. Panel D includes all control functions in Panel A to Panel C simultaenously. Three results for each of these samples are presented. OLS row presents the OLS estimate without including the control functions on the sample of observations where each control function can be calculated. Ctrl Common row presents the results with control functions using only common covariates between JC and CCC (enrollment age, age less than 18 indicator, highest grade level, hispanic status, whether helpd a previous job, whether graduated high school, household size, from rural hosehold, whether father is living, whether mother is living). Ctrl All row presents the results with control functions using common covariates as well as other variables included in the full specifications corresponding to Table III Column 6.

Appendix Table XXIII: Control Function Approach 1 (Assuming Treatment Effect is Same) with Interaction Terms

	(1)	(2)	(3)	(4)	(5)	(6)	(7)	(8)
Dependent Variable:	*Log Death Age*	*Log Death Age (Reweighted)*	*AIME*	*AIME (Reweighted)*	*Retirement Age*	*Retirement Age (Reweighted)*	*SSDI*	*SSDI (Reweighted)*
**Panel A: Using Education Only**								
OLS	0.013***	0.013***	47.882**	47.882**	0.394***	0.394***	-0.020	-0.020
	(0.004)	(0.004)	(21.416)	(21.416)	(0.141)	(0.141)	(0.014)	(0.014)
Ctrl Common	0.012	0.011*	57.054	51.523	0.470	0.442*	-0.036	-0.031
	(0.008)	(0.007)	(47.023)	(40.026)	(0.305)	(0.252)	(0.033)	(0.028)
ATE	0.013	0.013	52.079	50.189	0.416	0.406	-0.021	-0.021
Ctrl All	0.012	0.011	56.525	51.133	0.463	0.431	-0.035	-0.030
	(0.009)	(0.007)	(47.785)	(41.761)	(0.310)	(0.264)	(0.034)	(0.029)
ATE	0.013	0.013	51.866	50.110	0.415	0.406	-0.021	-0.021
N	7,722	7,722	4,613	4,613	5,446	5,446	4,575	4,575
**Panel B: Using Moved Only**								
OLS	0.013***	0.013***	48.286**	48.286**	0.391***	0.391***	-0.018	-0.018
	(0.004)	(0.004)	(21.354)	(21.354)	(0.142)	(0.142)	(0.014)	(0.014)
Ctrl Common	0.013***	0.013***	40.402*	42.409*	0.372**	0.381**	-0.020	-0.019
	(0.005)	(0.005)	(23.731)	(22.682)	(0.177)	(0.166)	(0.017)	(0.016)
ATE	0.013	0.013	47.791	47.236	0.389	0.389	-0.018	-0.018
Ctrl All	0.013***	0.013***	42.105*	42.034*	0.376**	0.383**	-0.020	-0.020
	(0.005)	(0.005)	(22.662)	(22.835)	(0.165)	(0.168)	(0.016)	(0.016)
ATE	0.013	0.013	47.970	47.511	0.389	0.390	-0.018	-0.018
N	7,703	7,703	4,600	4,600	5,432	5,432	4,562	4,562
**Panel C: Using Others Only**								
OLS	0.024*	0.024*	-17.604	-17.604	0.589*	0.589*	-0.112**	-0.112**
	(0.012)	(0.012)	(75.655)	(75.655)	(0.329)	(0.329)	(0.051)	(0.051)
Ctrl Common	0.036	0.007	72.517	19.778	2.260	1.959	-0.478	-0.574*
	(0.075)	(0.070)	(361.893)	(334.165)	(2.413)	(2.325)	(0.298)	(0.294)
ATE	0.021	0.021	-62.458	-70.389	0.505	0.514	-0.130	-0.130
Ctrl All	0.041	0.016	110.876	59.084	2.448	2.279	-0.458	-0.545*
	(0.075)	(0.072)	(355.196)	(343.685)	(2.422)	(2.419)	(0.300)	(0.302)
ATE	0.021	0.020	-58.998	-70.724	0.493	0.500	-0.130	-0.132
N	1,382	1,382	621	621	1,010	1,010	621	621
**Panel D: All Control Functions**								
OLS	0.025**	0.025**	-18.237	-18.237	0.606*	0.606*	-0.112**	-0.112**
	(0.012)	(0.012)	(76.171)	(76.171)	(0.327)	(0.327)	(0.050)	(0.050)
Ctrl Common	0.026	0.002	219.307	203.536	1.538	1.385	-0.538*	-0.630**
	(0.079)	(0.074)	(452.373)	(419.208)	(2.458)	(2.363)	(0.284)	(0.279)
ATE	0.023	0.021	-65.979	-66.181	0.495	0.475	-0.126	-0.126
Ctrl All	0.031	0.010	272.817	268.885	1.737	1.670	-0.539*	-0.635**
	(0.080)	(0.076)	(441.682)	(425.157)	(2.459)	(2.449)	(0.285)	(0.282)
ATE	0.022	0.021	-62.999	-65.859	0.492	0.472	-0.127	-0.128
N	1,362	1,362	611	611	995	995	611	611

Notes: Standard errors clustered at the level of county-by-year-quarter of enlistment in parentheses, *** p<0.01, ** p<0.05, * p<0.1. These specifications use control functions calculated under the assumption that treatment effect between CCC and JC are the same. Panel A-Panel B uses different includes different sets of control functions for the unweighted and the reweighted sample, where the weights are calculated using Hainmueller (2012) with relative disadvantages as inputs. In this table, we allow for heterogeneous treament effects and include control functions interacted with duration. We report both the coefficient on duration itself and the Average Treatment Effects (ATE) of duration from this specification. In Panel A, we only include control function generated using education as the short-run outcome. In Panel B, the short-run outcome is short-run mobility from the 1940 Census and WWII rolls. Panel C uses whether working, weeks worked, log wage as short-run outcomes. Panel D includes all control functions in Panel A to Panel C simultaenously. Three results for each of these samples are presented. OLS row presents the OLS estimate without including the control functions on the sample of observations where each control function can be calculated. Ctrl Common row presents the results with control functions using only common covariates between JC and CCC (enrollment age, age less than 18 indicator, highest grade level, hispanic status, whether helpd a previous job, whether graduated high school, household size, from rural hosehold, whether father is living, whether mother is living). Ctrl All row presents the results with control functions using common covariates as well as other variables included in the full specifications corresponding to Table III Column 6.

Appendix Table XXIV: Control Function Approach 2 (Assuming Selection Bias is Same) with Interaction Terms

	(1)	(2)	(3)	(4)	(5)	(6)	(7)	(8)
Dependent Variable:	*Log Death Age*	*Log Death Age (Reweighted)*	*AIME*	*AIME (Reweighted)*	*Retirement Age*	*Retirement Age (Reweighted)*	*SSDI*	*SSDI (Reweighted)*
**Panel A: Using Education Only**								
OLS	0.013***	0.013***	47.882**	47.882**	0.394***	0.394***	-0.020	-0.020
	(0.004)	(0.004)	(21.416)	(21.416)	(0.141)	(0.141)	(0.014)	(0.014)
Ctrl Common	0.010	0.010	45.435	43.403	0.601	0.576	-0.054	-0.049
	(0.010)	(0.010)	(60.458)	(56.559)	(0.382)	(0.352)	(0.043)	(0.040)
ATE	0.013	0.012	46.809	45.786	0.390	0.386	-0.019	-0.019
Ctrl All	0.013***	0.013***	48.564**	47.660**	0.410***	0.384***	-0.021	-0.019
	(0.004)	(0.004)	(21.454)	(21.633)	(0.141)	(0.143)	(0.014)	(0.014)
ATE	0.013	0.013	48.205	47.823	0.393	0.393	-0.020	-0.020
N	7,722	7,722	4,613	4,613	5,446	5,446	4,575	4,575
**Panel B: Using Moved Only**								
OLS	0.013***	0.013***	48.286**	48.286**	0.391***	0.391***	-0.018	-0.018
	(0.004)	(0.004)	(21.354)	(21.354)	(0.142)	(0.142)	(0.014)	(0.014)
Ctrl Common	0.013**	0.013**	39.702	40.164*	0.381**	0.385**	-0.021	-0.021
	(0.005)	(0.005)	(24.921)	(24.190)	(0.187)	(0.181)	(0.018)	(0.017)
ATE	0.013	0.013	47.734	47.797	0.390	0.391	-0.018	-0.018
Ctrl All	0.014***	0.014***	43.045*	44.523**	0.368**	0.381**	-0.015	-0.016
	(0.005)	(0.004)	(23.121)	(22.117)	(0.167)	(0.156)	(0.016)	(0.015)
ATE	0.013	0.013	47.812	47.590	0.391	0.393	-0.018	-0.017
N	7,703	7,703	4,600	4,600	5,432	5,432	4,562	4,562
**Panel C: Using Others Only**								
OLS	0.024*	0.024*	-17.604	-17.604	0.589*	0.589*	-0.112**	-0.112**
	(0.012)	(0.012)	(75.655)	(75.655)	(0.329)	(0.329)	(0.051)	(0.051)
Ctrl Common	0.043	0.037	-107.522	-93.499	2.433	2.591	-0.441	-0.540
	(0.090)	(0.092)	(400.875)	(389.297)	(2.910)	(3.045)	(0.358)	(0.376)
ATE	0.022	0.021	-49.609	-54.658	0.499	0.496	-0.120	-0.111
Ctrl All	0.018	0.013	-75.601	-19.308	0.023	0.397	-0.063	-0.114**
	(0.016)	(0.013)	(89.050)	(79.373)	(0.468)	(0.381)	(0.078)	(0.056)
ATE	0.021	0.019	-25.270	-32.452	0.469	0.437	-0.107	-0.103
N	1,382	1,382	621	621	1,010	1,010	621	621
**Panel D: All Control Functions**								
OLS	0.025**	0.025**	-18.237	-18.237	0.606*	0.606*	-0.112**	-0.112**
	(0.012)	(0.012)	(76.171)	(76.171)	(0.327)	(0.327)	(0.050)	(0.050)
Ctrl Common	0.036	0.030	-48.246	-10.489	1.511	1.733	-0.268	-0.396
	(0.095)	(0.097)	(537.842)	(526.594)	(3.018)	(3.126)	(0.368)	(0.375)
ATE	0.023	0.022	-38.178	-43.353	0.530	0.527	-0.114	-0.105
Ctrl All	0.020	0.015	-75.279	-15.789	-0.032	0.435	-0.037	-0.102*
	(0.020)	(0.015)	(95.401)	(81.581)	(0.521)	(0.410)	(0.084)	(0.059)
ATE	0.022	0.020	-25.170	-33.124	0.509	0.472	-0.102	-0.095
N	1,362	1,362	611	611	995	995	611	611

Notes: Standard errors clustered at the level of county-by-year-quarter of enlistment in parentheses, *** p<0.01, ** p<0.05, * p<0.1.These specifications use control functions calculated under the assumption that treatment effect between CCC and JC are different but selection bias is the same. Panel A-Panel B uses different includes different sets of control functions for the unweighted and the reweighted sample, where the weights are calculated using Hainmueller (2012) with relative disadvantages as inputs.In this table, we allow for heterogeneous treament effects and include control functions interacted with duration. We report both the coefficient on duration itself and the Average Treatment Effects (ATE) of duration from this specification. In Panel A, we only include control function generated using education as the short-run outcome. In Panel B, the short-run outcome is short-run mobility from the 1940 Census and WWII rolls. Panel C uses whether working, weeks worked, log wage as short-run outcomes. Panel D includes all control functions in Panel A to Panel C simultaenously. Three results for each of these samples are presented. OLS row presents the OLS estimate without including the control functions on the sample of observations where each control function can be calculated. Ctrl Common row presents the results with control functions using only common covariates between JC and CCC (enrollment age, age less than 18 indicator, highest grade level, hispanic status, whether helpd a previous job, whether graduated high school, household size, from rural hosehold, whether father is living, whether mother is living). Ctrl All row presents the results with control functions using common covariates as well as other variables included in the full specifications corresponding to Table III Column 6.
